
JOURNAL INFORMATION
==============================
NLM Title Abbreviation: J Child Orthop
Journal ID: J Child Orthop
NLM Journal ID: 101313582

Journal of Children's Orthopaedics

ISSN: 1863-2521
EISSN: 1863-2548
Publisher: SAGE Publications

ARTICLE INFORMATION
==============================
PMCID: PMC4353813
PMID: 25716893
DOI: 10.1007/s11832-015-0640-5
Article ID: 640
Article version: 1
Subjects: Abstracts

EPOS 34th Annual Meeting

Electronic publication date: 2015 Feb 26
Print publication date: 2015 Apr
Volume: 9
Issue: Suppl 1
First page: 67
Last page: 119
Copyright: © The Author(s) 2015
License: This article is distributed under the terms of the Creative Commons Attribution License which permits any use, distribution, and reproduction in any medium, provided the original author(s) and the source are credited.
License URL: https://creativecommons.org/licenses/by/4.0/

issue-copyright-statement: © The Author(s) 2015

==============================

## EP1

### MRI evaluation of resection margins in bone tumour surgery

Simon Vandergugten[1], Pierre-Louis Docquier[2]

[1]UCL Saint-Luc, Bruxelles, Belgium
[2]Saint-Luc university Hospital, Brussels, Belgium

**LEVEL-II/Tumours**

*Materials and Methods*: In 12 patients operated on for bone sarcoma resection, a postoperative magnetic resonance imaging of the resection specimens was obtained in order to assess the surgical margins. Margins were classified according to MRI in R0, R1, R2 by three independent observers: a radiologist and two orthopaedic surgeons. Final margin evaluation (R0, R1, R2) was assessed by a confirmed pathologist.
*Results*: Agreement for margin evaluation between the pathologist and the radiologist was perfect ($k = 1$). Agreement between the pathologist and an experienced orthopaedic surgeon was very good ($k = 0.87$) while it was fair between the pathologist and a junior orthopaedic surgeon ($k = 0.25$).
*Discussion*: MRI should be considered as a tool to give quick information about the adequacy of margins and to help the pathologist to focus on doubtful areas and to spare time in specimen analysis. But it may not replace the pathological evaluation that gives additional information about tumor necrosis.
*Conclusion*: This study shows that MRI extemporaneous analysis of a resection specimen may be efficient in bone tumor oncologic surgery, if made by an experienced radiologist with perfect agreement with the pathologist.

## EP2

### Are 6 weeks of spica cast immobilization adequate for achieving hip stability following closed reduction of unstable DDH?

Eyal Mercado[1], Daniel Weigl[1], Taly Becker[1], Elhanan Bar-On[1]

[1]Schneider Medical Center, Petach Tikva, Israel

**LEVEL-II/DDH**

*Purpose*: To examine the safety and efficacy of a protocol of 6 weeks of casting followed by abduction bracing of unstable DDH compared to the traditional 12 weeks casting period.
*Methods*: The study group included 20 patients comprising all patients treated by closed reduction and spica casting due to DDH in our institution between July 2010 and July 2012. All patients underwent initial treatment by examination under anesthesia, adductor tenotomy if needed, arthrography, closed reduction and application of a spica cast in the human position. Six weeks later the patients were reexamined under anesthesia and a repeat arthrogram was performed. If the examination and arthrography revealed a stable and congruent reduction the cast was discontinued and the patients were fitted with an abduction brace. Otherwise a new spica cast was applied for an additional 6 weeks. A cohort of 20 patients treated in 2008–2009 by the traditional protocol of two 6 weeks casting periods served as our control group. Patients were followed for a minimum of 1 year (mean 19 months).
*Results*: Patient demographics and DDH characteristics were similar in both groups. Mean age at admission was approximately 20 weeks. 20 patients were treated with 22 affected hips. All hips were initially unstable. At the 6 weeks examination, 17 hips (77.2 %) were stable and congruent, and were converted to a brace. The remaining 5 hips were either unstable or had an abnormal arthrogram and were recasted for an additional 6 weeks. At final follow up all 17 hips in the 6 weeks casting group remained stable and well contained with no radiographic signs of AVN, and none of them required additional surgery. One patient in the study group who required recasting and three patients in the control group underwent additional surgery ($P < 0.01$). After removal of the cast the patients in the study group required an abduction device for a mean of 9.7 weeks compare to 6.2 in the control group ($P < 0.05$)
*Conclusions*: In patients treated by closed reduction and casting for unstable DDH, a 6 weeks casting period is adequate and safe providing the hip is stable and congruent at 6 weeks. The patients will require a longer post-operative period in an abduction device
*Significance*: Shortening the period of spica casting can reduce cast complications, patient discomfort and parent inconvenience

## EP3

### The prevalence of bifid iliopsoas tendon on MRI scans in children

Thomas Crompton[1], Claire Lloyd[1], Michail Kokkinakis[1], Fabian Norman-Taylor[1]

[1]The Evelina London, London, UK

**LEVEL-IV/Hip/Lower extremity**

*Objective*: Variation in anatomy of the iliopsoas tendon is important information for orthopaedic surgeons operating around the hip. The aim of this study is to identify the prevalence of bifid iliopsoas tendons in children on magnetic resonance imaging (MRI).
*Methods*: MRI hip and pelvis images of 50 sequential children, aged 7–15 years were retrieved from our radiology database at the Evelina London Children's Hospital from 2007 to 2013. Included were 37 children with imaging of both hips and 13 children with imaging of one hip only. Therefore, our study was based on a total of 87 hips.
*Results*: At least 1 bifid tendon was noted in 13 children (26 %). 5 children out of a total of 37 (14 %) with both hips adequately imaged had bilateral bifid tendons. From all 87 hips adequately imaged there were 18 hips (21 %) identified with 2 discreet distal tendons of iliopsoas.
*Conclusions*: Bifid iliopsoas tendon is noted anecdotally by surgeons but is only reported in scattered case reports and a few anatomical studies until very recently. Our finding is that a bifid iliopsoas tendon with two distinct tendinous components at the level of the hip joint is quite common. This has clinical significance in particular in children's orthopaedic surgery when an adequate illiopsoas release is important.

## EP4

### Staged soft tissue, bony and Ilizarov procedures for correction of leg and foot deformities in tibial hemimelia

Amin Abdelrazek Ahmed[1]

[1]Alexandria University, Alexandria, Egypt

**LEVEL-IV/Limb reconstructions**

*Background*: The incidence of tibial hemimelia is very rare being about 1 per million in the reported literature.
The deformity varies according to the type of the deficiency, the involved leg is short and the foot is held in equinovarus. In type II, partial development of the tibia eventually occurs, and a relatively functional knee joint is present, the fibula is usually normal in size, but the head is proximally dislocated
*Materials and Methods*: The material of this study included eight patients with tibial hemimelia (Jones type II). Five were boys and three girls. The average age of the patients at the time of the first operation was 2.3 years (1–4 years) and the average follow up period was 31 (14–60 months). Right leg was the affected side in three while five patients had left sided tibial hemimelia.
Operative steps:
Soft tissue release and centralization of the fibula in the ankle was done followed by tibiofibular fusion between the proximal tibia and the fibula (side to side) at the age of 3.5–4 years in all patients and to be followed by Ilizarov distraction at the age of 5 years to pull the fibula down and then continue lengthening to equalize limb length inequality.
*Results*: At the end of follow up period (31 months) good results were achieved in the eight cases with good range of knee movement and plantigrade foot, all patients could walk independently and without pain. All cases showed full satisfaction to the patients and their parents. Bone consolidation of the distraction site was achieved in all cases with 4.5–6.5 cm gained tibial length. There was no limb length inequality in six cases with residual shortening in two cases (2 and 2.5 cm) which did not affect the final satisfactory results.
*Conclusion*: Early Soft tissue correction, tibiofibular fusion followed by Ilizarov distraction gave satisfactory results in cases of tibial hemimelia (Jones type 2) which were difficult to be treated by the conventional methods

## EP5

### Physeal bar formation after paediatric medial malleolus fractures

Anthony Riccio[1], Matthew Abbott[2], Robert Wimberly[1], Philip Wilson[3]

[1]Texas Scottish Rite Hospital, Dallas, USA
[2]University of Michigan, Ann Arbor, USA
[3]Texas Texas Scottish Rite Hosp, Dallas, USA

**LEVEL-III/Trauma/Lower extremity**

*Introduction*: Paediatric medial malleolus fractures are commonly Salter–Harris (SH) III or IV fractures of the distal tibia and are associated with a risk of physeal bar formation and subsequent growth disturbance. The reported incidence of physeal bar formation varies from 6 to 50 %. Previous studies have demonstrated SH classification, fracture displacement, adequacy of reduction, and time to treatment as predictive factors of bar formation. The purpose of this study was to determine the incidence of physeal bar formation following paediatric medial malleolus fracture and evaluate the patient and fracture characteristics predictive of physeal bar formation.
*Methods*: We retrospectively reviewed 79 consecutive paediatric patients from 2007 to 2013 that sustained either an isolated medial malleolar or a bimalleolar ankle fracture. 42 of 79 patients had >3 months of radiographic follow-up and comprised our study population. Medical records were reviewed to determine age, sex, weight, mechanism of injury, method of treatment, and need for further surgery. Radiographs were reviewed to assess the initial fracture displacement, adequacy of fracture reduction, SH type, presence of an associated fibula fracture, percentage of the physeal disruption from the fracture, and physeal bar formation. Mann–Whitney U and Chi squared tests were used to compare variables between those injuries that developed a physeal bar and those that did not.
*Results*: 22 of 42 patients (52.4 %) developed a physeal bar. The average time to diagnosis of physeal bar was 4.9 months (1.6–11.8 months). 27 % (6/22) of bars were diagnosed at >6 months from injury. 4 of 22 physeal bars (18.2 %) required further surgery. The adequacy of reduction was predictive of physeal bar formation with the patients that developed a bar having a mean reduction to 1.2 mm and the non-bar patients being reduced to a mean of 0.8 mm ($p = 0.03$). All other studied variables were not predictive including SH type and amount of initial fracture displacement ($p > 0.05$).
*Conclusion*: The adequacy of reduction was the only predictive factor for physeal bar formation following a paediatric medial malleolus fracture. However, obtaining fracture reduction of <2 mm of displacement still may result in a high rate of bar formation. Given the high incidence of physeal bar formation and frequent diagnosis beyond 6 months from injury, we recommend radiographs on all pediatric medial malleolar fracture patients at 1 year following injury.

## EP6

## Lengthening in congenital radial club hand type IV

Ahmad Allam[1]

[1]Banha University, Banha, Egypt

**LEVEL-I/Limb reconstructions**

*Background*: The predominant characteristics of radial club hand are defects of the radius, carpal bones, and radial rays, particularly the thumb. A severely bowed ulna may contribute further to the clubbed appearance. The primary objective of lengthening is to improve the appearance (and function if possible) of the upper limb.
*Patients and Methods*: Eight patients with Heikel's type IV congenital radial club hand (with previous wrist centralization) were operated upon. The patients' average age was 6 years. Average ulnar shortening was 12.5 cm (representing 47 % of contralateral forearm length) with mean angular deformity of 40°. A one stage procedure including acute correction of the deformity via an ulnar distal closing wedge osteotomy and the application of a mono-plane lengthening device was done. Lengthening was started—from the osteotomy site—10 days later.
*Results*: Ulnae were lengthened 6–9 cm (average 7.5 cm); representing 30–62 %of their original length (average 54 %) and an average of 83 % of contralateral forearm length; with a healing index of 36 days (range 28–43 days).The angular deformity was completely corrected in all patients; with late mild recurrence of the deformity in one patient. No early or late major complications were encountered during a follow up period of 4.5 years.
*Conclusion*: This one stage procedure of acute correction of the deformity and lengthening of the ulna using a mono-plane lengthening device is a simple technique with satisfactory results and few reported complications.

## EP7

## The efficacy and utility of cast wedging in adolescent tibial shaft fractures

Janay McKie[1], Robert Wimberly[2], Dana Hensley[3], Jennifer Nance[4], Anthony Riccio[2]

[1]Shreveport Shriner's Hospital, Shreveport, USA
[2]Texas Scottish Rite Hospital, Dallas, USA
[3]Tripler Army Medical Center, Honolulu, USA
[4]Childrens Medical Center, Dallas, USA

**LEVEL-III/Trauma/Lower extremity**

*Purpose*: To determine the efficacy of cast wedging in the management of adolescent tibial shaft fractures that displace following an initial closed reduction and casting.
*Methods*: An IRB approved retrospective review of all adolescents presenting to a single level 1 paediatric trauma center with a closed tibial or tibial and fibular shaft fracture from 2003 to 2013 was performed. Patients aged 10–16 years with displaced fractures managed by reduction and long leg casting were included for analysis. Demographic data was recorded. Radiographs were reviewed to determine fracture location and pattern. Angulation was measured at presentation, following initial reduction, following any further intervention, and at osseous union. Final coronal and sagittal angulation was compared between patients treated with cast wedging and those managed successfully with a single cast application.
*Results*: 156 patients sustained closed displaced tibial or tibial and fibular fractures during the study period that were treated with reduction and application of a long leg cast. 106 patients required no further intervention. 50 patients had a second intervention of which, 40 were managed with wedging of the cast. There were no significant differences in the demographics, fracture location, or fracture pattern between the single cast group and the wedging group. Patients in wedged group were more likely to have a fibular fracture at the same level as the tibia fracture. At radiographic union, there was a significant difference in coronal angulation between the single cast group (3.7°) and the wedging group (3.0°) ($p = 0.0272$). No significant difference was identified in sagittal angulation (3.1° vs. 3.5°) ($p = 0.2642$). Wedging of the cast for a coronal plane deformity resulted in a significant decrease in coronal angulation (6.6°–3.1°) ($p = 0.0002$) at final follow-up. Wedging of the cast for a sagittal plane deformity resulted in a significant decrease in sagittal angulation (7.0°–4.0°) ($p = 0.0131$) as well as coronal angulation (4.9°–2.8°) ($p = 0.0044$) at final follow-up.
*Conclusions*: Approximately one-third of displaced tibial shaft fractures in adolescents will require an intervention following initial closed reduction and casting. Wedging of the cast is an effective means of changing both sagittal and coronal plane angulation and can result in improved coronal alignment and equivalent sagittal alignment to fractures treated in a single cast.
*Significance*: Cast wedging is an effective technique to improve angulation in adolescent tibial shaft fractures that have lost alignment following closed reduction and casting.

## EP8

## Pelvic height increase and scoliosis following salter osteotomy and pemberton aacetabuloplasty in developmental dysplasia of the hip

Cheng-Wei Wang[1], Kuan-Wen Wu[2], Ting-Ming Wang[2], Shier-Chieg Huang[3], Ken N. Kuo[4]

[1]New Taipei City Hospital, New Taipei City, Taiwan
[2]National Taiwan University H, Taipei City, Taiwan
[3]China Medical University H, Taichung City, Taiwan
[4]Taipei Medical Unversity, Taipei City, Taiwan

**LEVEL-II/DDH**

*Purpose*: Salter osteotomy and Pemberton acetabuloplasty are common procedures for the deficient acetabulum in developmental dysplasia of the hip. However, the transiliac lengthening effect of these two procedures and its effect on pelvic imbalance or lumbar curvature remains unanswered. The purpose of the study is to review our long-term results regarding pelvic imbalance and lumbar curvature after these two surgeries.
*Methods*: Forty-two patients who underwent either a Salter or Pemberton procedure at one institution between January 1981 and December 2000 and were available for follow-up during the first and second decade were evaluated retrospectively. We measured the pelvic height increase, iliac crest tilt and sacral tilt at the first and second decade follow-up. At the second decade follow up, the Cobb angle for the lumbar spine was measured and the patient completed the SF-36 and Harris hip score questionnaires.
*Results*: At the first decade follow-up, Salter osteotomy lengthened the pelvis more than Pemberton acetabuloplasty did as judged by pelvic height increase [Salter osteotomy, 10.1 % (95 % CI 7.86, 12.33); Pemberton acetabuloplasty, 4.3 % (95 % CI 3.22, 5.40), $p < 0.001$]. But the difference in pelvic height increase became non significant between the two groups at the second decade follow-up [Salter osteotomy, 4.4 % (95 % CI 2.73, 5.98); Pemberton acetabuloplasty, 3.1 % (95 % CI 2.30, 3.94), $p = 0.249$]. As for lumbar Cobb angle, there were no significant differences between the two groups [Salter osteotomy, 3.1 degrees (95 % CI −1.37, 7.56); Pemberton acetabuloplasty, 3.3° (95 % CI −0.07, 6.67), $p = 0.906$]. The correlation between radiographic parameters for pelvic imbalance (pelvic height increase, iliac crest tilt and sacral tilt) and lumbar Cobb angle was not strong (0.31, 0.43, 0.59). 41 of the 42 patients (97.6 %) had some degree of coronal lumbar curve, and 30 of the 41 patients (73.1 %) had compensatory curves. The SF-36 and Harris hip scores were good and showed no differences between the two groups.
*Conclusions*: Our study suggests that both Salter and Pemberton surgery cause pelvic height increase, iliac crest tilt and sacral tilt. Salter osteotomy tends to increase the pelvic height more. After longer follow-up, the hemipelvic lengthening effect may diminish. Though the degree of pelvic imbalance decreased with time, we found a high prevalence of lumbar coronal curve and most of the curve was compensatory to pelvic imbalance. The degree of coronal lumbar curve was not significantly different between the two groups.

## EP9

### An anatomical study of the greater trochanter starting point for intramedullary nailing in the skeletally immature

Paul Park[1], Doug Weinberg[1], Kathleen Farhang[1], Raymond Liu[1]

[1]Case Western Reserve University, Cleveland, USA

**LEVEL-II/Basic science**

*Purpose*: Trochanteric entry femoral nails have been increasing in popularity in the pediatric population for stabilization in fractures and osteotomies. Characterization of greater trochanter entry position in the sagittal plane in the pediatric population has not yet been well studied.
*Methods*: Ninety cadaveric femora ages 8–20 years were studied in an apparent neck-shaft angle (ANSA) position, with distal condyles flat on the surface; and a true neck-shaft angle (TNSA) position, with internal rotation to neutralize femoral anteversion. A marker was placed at the apex of the greater trochanter. Anterior and lateral offset were measured in lateral and anteroposterior photographs, respectively, as the perpendicular distance from the marker to the center of the intramedullary canal. The effect of rotational position (ANSA versus TNSA) of the proximal femur was compared using intraclass correlation coefficient (ICC) for anterior and lateral offset. Correlation between age and anterior and lateral offset was evaluated with linear regression analysis.
*Results*: Mean age was 14.7 ± 3.6 years. Mean anterior displacement of the trochanteric apex relative to the intramedullary canal was 4.9 ± 3.0 and 4.6 ± 3.4 mm in the ANSA and TNSA positions, respectively. Mean lateral displacement was 10.1 ± 4.4 and 9.3 ± 4.1 in the ANSA and TNSA positions, respectively. The intraclass coefficient (ICC) for anterior offset in the ANSA versus TNSA position was 0.704 (95 % CI 0.559–0.807), and for lateral offset was 0.900 (95 % CI 0.813–0.944). Change was minimal for anterior offset in the ANSA and TNSA positions versus age ($r2 = 0.022$; $r2 = 0.001$). There was a mild increase in lateral offset in the ANSA and TNSA positions with increasing age ($r2 = 0.333$, $p < 0.001$; $r2 = 0.203$, $p < 0.001$).
*Conclusion*: Neither anterior nor lateral offset are significantly affected by rotational positioning of the proximal femur. The apex of the greater trochanter is consistently anterior to the center of the intramedullary canal across all ages. Lateral offset increases with age.
*Significance*: This study demonstrates that the greater trochanteric apex is located approximately 5 mm anterior to the center of the intramedullary canal in juveniles and adolescents. This value remains relatively constant throughout the age range generally treated with trochanteric entry nails. Standard nail designs have accounted for the lateral position of the trochanteric apex in the coronal plane, but not for the anterior position of the apex in the sagittal plane. We recommend inserting the guidewire 5 mm posterior to the apex of the trochanter and confirming coronal and sagittal position with fluoroscopy.

## EP10

### The role of ultrasound investigation: sacral dimples and other stigmata of spinal dysraphism in infants

Jung yun Bae[1]

[1]Pusan National University, Yangsan City, Republic of Korea

**LEVEL-IV/Spine**

*Study design*: A retrospective study.
*Objectives*: To evaluate association of minor cutaneous stigmas with occult spinal dysraphism (OSD), as well as the use of, ultrasound as a screening method for OSD.
*Summary of Literature Review*: OSD can be associated with various cutaneous markers.Ultrasound of the spine is an effective, non-invasive screening method.
*Materials and Methods*: Over a 5-year period (2009–2013) a total of 180 infant lumbarspines were scanned, for various clinical indications. Ninety-seven patients had abnormalities detected on postnatal clinical examination; most of these had various cutaneous markers, eighty-three had other congenital abnormalities. Type of skin stigmata and/or congenital anomalies and lumbar ultrasound results were reviewed for all patients.

*Results*: In normal infants, three of 97 had abnormalities. One of three had OSD. Eighteen of 83 infants with congenital anomalies had abnormalities, eleven of 18 had OSD. Infants with congenital anomalies were six times more likely to have OSD than normal infants (OR 5.98, 95 % CI 1.927–18.612, $p = 0.001$) and there was no significant correlation between the presence of minor skin lesions and the presence of dysraphism.
*Conclusions*: The so-called minor skin lesions were not markers of OSD in normal infant. However, in 1 patients, ultrasound was decisive for the surgical decision. From the statistical point of view, there is no indication for complementary examinations in patients with minor skin stigmas. However, because of the feasibility, simplicity, and low cost of the spinal ultrasound, the examination is justified in the benefits of early diagnosis.

## EP11

## Kinematic and kinetic evolution of the spine during growth

Sébastien Pesenti[1], Vincent Pomero[2], Benjamin Blondel[3], Emilie Peltier[1], Elke Viehweger[1], Jean-Luc Jouve[1]

[1]Timone Enfants, Marseille, France
[2]Timone Enfants, Marseille, France
[3]Timone, Marseille, France

**LEVEL-III/Spine**

*Introduction*: With the acquisition of bipedalism, many anatomical and postural changes occur during childhood. Among these changes, the adaptation of the spine seems to be fundamental to maintain the erect position in the young child. The aim of our study was to describe the evolution of spine balance during the acquisition of the gait during childhood using gait analysis tools.
*Materials and Methods*: Thirty-six healthy children aged from 3 to 16 years old were included in this study and performed a gait analysis (9 m-walk). Different kinematic parameters were recorded and analyzed such as Thoracic Angle (TA), Lumbar Angle (LA) and Sagittal Vertical Axis (SVA). The kinetic parameters were the net reaction moments (in Nm/kg) at the thoracolumbar and at the lombasacral joint.
*Results*: TA and LA curves were not statistically correlated to the age (respectively, $p = 0.32$ and $p = 0.41$). SVA increased significantly with the age ($p < 0.001$). The flexion–extension moments at the lumbosacral joint were statistically correlated to the age ($p = 0.003$), meaning that mechanical constraints in sagittal plane at the lumbosacral joint increase with age. The torsion moments at thoracolumbar and lumbosacral joints were statistically correlated to the age ($p = 0.0002$ and $p = 0.0006$), meaning that mechanical constraints in transversal plane at thoracolumbar and lumbosacral joints decrease with age.
*Discussion*: Only few authors have studied dynamic evolution of the spine according to the age in gait analysis. These results show that the acquisition of spinal curvature is a morphological characteristic appearing very early in childhood, before the age of 3. The kinetic analysis showed that with age, a decrease of torsional constraint occurs while an increase of sagittal constraint is observed. These changes in spine biomechanics are due to the major role of the trunk in the acquisition of bipedalism, allowing stabilization despite the immaturity of the lower limbs. Secondarily, these constraints will change to be applied in the sagittal plane.

## EP12

## Surgical treatment of congenital and obligatory dislocation of the patella

Ronen Sever[1], Shlomo Wientroub[1], Michael Fishkin[1], Moshe Yaniv[1]

[1]Dana Children's Hospital, Tel Aviv, Israel

**LEVEL-IV/Hip—Lower extremity**

*Introduction*: Patellar dislocation is classified into: fixed, obligatory, recurrent and acutely traumatic. Left untreated, it leads to multiple deformities and contractures, resulting in significant disability. There is no relevant evidence that the surgical options suggested can solve the problem. In 1976, Stanisavljevic et al. described a surgical technique based on their suggested pathogenesis of deficient internal rotation of the quadriceps myotome during embryonic life but its effectiveness and validity were lately questioned by Camanthias et al. (2014)
*Patients and Methods*: We reviewed the records of 12 patients (15 knees) who underwent this procedure for fixed or obligatory dislocation of patella. Our groups was 9 females, 3 males, mean age was 7.8 years (3.3–12.7); all patients had an open physis. Seven patients were classified as fixed type with a mean age of 9 years (3.4–12.7) and 5 as the obligatory type with a mean age of 7.1 years (3.3–12.6). Outcome measures included: pain, stability, range of motion (ROM), Pedi-IKDC knee function score and the PODCI global function score. 6 patients were diagnosed with Down syndrome, 3 were idiopathic dislocators, 2 had Larsen syndrome and one Nail Patella syndrome.The operative technique included extensive subperiosteal quadriceps realignment and soft tissue medial plication followed by distal realignment through a patellar tendon splitting. Post-operatively, the leg was immobilized for 3 weeks followed by ROM and muscle control exercises.
*Results*: Mean follow up was 24 months (6–66 months). For all patients this was the first operative procedure for the treatment of their patellar instability. All patients, but one, had stable patella with no recurrence of dislocation or pain, post-operative knee extension was improved significantly; flexion remained unchanged. Average post-operative Pedi-IKDC score was $86.25 \pm 6.92$ (78–98), with a significantly higher score among the idiopathic dislocation group. Post-operative PODCI scores were significantly higher ($p < 0.001$) among the idiopathic group as well. One patient had recurrent dislocation following a fall a year after the operation and required a second operation.
*Discussion*: Our results are significantly better than Camanthias' et al. who performed this surgery on significantly older population with wider inclusion criteria. Younger age and higher probability of remodeling of the trochlear groove, as well as the use of this operation as a first line treatment and not as a salvage procedure, are key factors in our superior results.
*Conclusion*: Stanisavljevic' procedure is an efficient first technique for fixed and obligatory patellar dislocation. Early referral for operative treatment is a key factor for successful results.

## EP13

### Outcome of displaced fractures of the distal metaphyseal–diaphyseal junction of the humerus in children treated by elastic stable intramedullary nails

Lorenza Marengo[1], Federico Canavese[2], Mattia Cravino[1], Vincenzo De Rosa[3], Marie Rousset[4], Antoine Samba[2], Mounira Mansour[2], Antonio Andreacchio[1]

[1]R M Children's Hospital, Turin, Italy
[2]University Hospital Estaing, Clermont Ferrand, France
[3]Bellinzona and Valli Hospital, Bellinzona, Switzerland
[4]University Hospital Estaing, Clermon Ferrand, France

**LEVEL-IV/Upper extremity only**

*Purpose*: In skeletally immature patients, fractures occurring at the distal humeral metaphyseal-diaphyseal junction, just proximal to the olecranon fossa, are rare. Surgical management of such injuries traditionally consisted of closed or open reduction and internal fixation with Kirschner wires. In recent years, elastic stable intramedullary nailing (ESIN) has been suggested as the treatment of choice for these fractures, resulting in stable reduction and good rotational control. The main objective of this study is to evaluate the clinical and radiographic outcomes of displaced fractures of displaced the distal humeral metaphyseal-diaphyseal junction fractures in children treated by ESIN.
*Methods*: From January 2011 to December 2012, 14 children with fractures of the distal humeral metaphyseal–diaphyseal junction were surgically treated by ESIN in three different institutions (Turin, Claremont Ferrand and Bellinzona). Only patients with humeral fractures of the distal metaphyseal-diaphyseal junction according to Fayssoux's definition were included. All patients were treated according to the surgical technique described by Métaizeau. One year after the index surgery, patients were asked to answer the short version of the Disabilities of the Arm, Shoulder and Hand outcome questionnaire (Quick DASH®).
*Results*: Fourteen patients met the inclusion criteria. The average patient age at the time of injury was 9.7 years (range 3.6–13.7). Seven fractures were classified as transverse, six oblique and one comminuted. Each of the 14 fractures reduced by closed manipulation and stabilized using two titanium elastic nails. The mean follow-up was 28.1 months (range 20–38). Radiologically, no secondary displacement, nail migration, loss of fixation, consolidation delay, nonunion, or re-fracture was noted. All patients returned to their previous daily and sport activities without discomfort or difficulty and they were free of pain at their last follow-up visits. The mean Quick DASH® score was 0.81 (range 0–6.8).
*Discussion*: According to our results, the treatment with ESIN of displaced distal humeral metaphyseal–diaphyseal fractures results in good clinical, radiological and functional outcomes. This is the first report about ESIN of this kind of fracture. This method provides good rotational control, but cannot completely prevent flexion or extension of the distal fragment. Residual transverse plane malalignment of the distal fragment in flexion or extension will not cause any major functional limitation, and it will eventually remodel during growth.
*Conclusions*: We recommend surgery for displaced fractures of the distal humeral metaphyseal–diaphyseal junction. ESIN leads to stable reduction, good rotational control and fast mobilization.

## EP14

### Prevalence of vitamin D deficiency in Korean children presenting with nonspecific lower extremity pain

Sun Young Joo[1]

[1]Incheon St. Mary's Hospital, Incheon, Republic of Korea

**LEVEL-III/Other/varia**

*Purpose*: Although interest in the role played by vitamin D in bone health is increasing, little is known about the role of this vitamin in musculoskeletal pain in children. This study aimed to assess the prevalence of vitamin D deficiency in children presenting with nonspecific lower extremity pains.
*Methods*: From 2011 to 2012, 183 children underwent evaluation for nonspecific lower extremity pains. Patients with a valid cause for these pains, such as fractures or transient synovitis, were excluded, as were those with underlying medical conditions such as cerebral palsy and metabolic disease. Finally, 140 patients met the inclusion criteria. Levels of serum 25-hydroxy vitamin D [25-(OH)D], the ideal indicator of vitamin D status, were determined in these children.
*Results*: There were 87 boys (62.1 %) and 53 girls (37.9 %). The mean age at the presentation was 5.2 years (range 2–15). The serum 25-(OH)D levels were <10 ng/mL in 5.7 % patients, <20 ng/mL in 51.4 % patients, <30 ng/mL in 37.9 % patients, and >30 ng/mL in only 5.0 % patients. Most patients visited the hospital in winter (41.4 %) (summer, 12.9 %), and the serum 25-(OH)D levels were the lowest in winter (17.2 ± 5.5 ng/mL).
*Conclusion*: This study found a high prevalence of vitamin D deficiency/insufficiency in Korean children with nonspecific lower extremity pains, possibly indicating an etiological role of vitamin D deficiency in these pains. More attention should be directed toward vitamin D and its role in optimization of bone health.

## EP15

### Transphyseal sliding flexible intramedullary nailing: experimental study

Dmitry Popkov[1], Natalia Kononovich[2], Roman Shutov[1], Dominique Barbier[3], Pierre Lascombes[4], Pierre Journeau[3]

[1]Ilizarov Center, Kurgan, Russian Federation
[2]Ilizarov Center, Kurgan, Russian Federation
[3]CHU Nancy, Nancy, France
[4]HUG Genève, Genève, Switzerland

**LEVEL-II/Trauma/Upper extremity**

*Purpose*: Transphyseal sliding flexible intramedullary nailing (TSFIN) is applied in children affected with diseases which reduce bone density, such as osteogenesis imperfecta. The aim of this experimental study was to evaluate the longitudinal growth of the tibia in conditions of SFIN in the intact tibia (series I), in modeling of a transverse fracture (series II) and also in combination of TSFIN and subperiosteal positioning of implants (series III).

*Methods*: The study comprised three series of six puppies each: the animals were aged 6 months. In series I, TSFIN of an intact tibia was performed: series II, TSFIN associated with a transverse tibial fracture and in series III. TSFIN along with subperiosteal insertion of the NiTi net with elastic nails integrated in it while transverse osteotomy of the tibial bones. The animals were observed until the end of natural growth.
*Results*: SFIN induced a reduction in growth of the operated tibia. On average, relative loss of residual growth of operated tibia (for the period from intervention to the close of physis) was 17 % in series I, 14.9 % in series II and 18.3 % in series III. Eccentric insertion of transphyseal nails caused angulation with growth. Consolidation of bone fragments in series II and III occurred in the same time frame. Periosteal and endosteal reactions contributed to thickening of the cortex.
*Conclusion*: TSFIN slows down longitudinal bone growth. In order to prevent angulation, telescopic systems should be positioned close to the center of the physes. A subperiosteal scaffold does not impair consolidation of bone fragments and can be associated with intramedullary transphyseal osteosynthesis.

## EP16

### Aberrant subtalar joint morphology is associated with flexible flatfeet in children

Alpesh Kothari[1], Julie Stebbins[2], Amy Zavatsky[1], Tim Theologis[1]

[1]Oxford University, Oxford, UK
[2]Oxford Gait Laboratory, Oxford, UK

**LEVEL-II/Foot and ankle**

*Introduction*: Flatfeet are commonly believed to be the affliction of the overweight and/or flexible child. The role of bony morphology has largely been ignored in the literature. There is much variability in the shape and number of articular facets of the subtalar joint in humans and this could affect foot posture. The aim of this study was to investigate the role of subtalar joint morphology in determining foot posture. We hypothesised that children with increased joint laxity and an absent anterior subtaloid articulation were more likely to have a flat foot posture and associated symptoms and limitations.
*Methods*: 84 children (aged 8–15) were classified as having flatfeet (FF) or neutral feet based on clinical examination. The arch height index (AHI)1 was used as a continuous measure of foot posture. Subjects underwent a sagittal T1-weighted MRI of their feet. Scans were assessed for the presence or absence of the anterior subtaloid articular facet of the calcaneum2. Body mass index (BMI) was calculated, flexibility assessed using the lower limb assessment score (LLAS), and the Oxford Ankle Foot Questionnaire for Children (OxAFQ_C) was collected. Stepwise multiple linear regression, and Pearson's correlation were undertaken to assess the effect of these factors on AHI. Regression was adjusted for paired foot observations ($\alpha = 0.05$).
*Results*: The mean age of subjects was 11.91 ± 2.23 years. Male to female ratio was 45:39. Median LLAS was 4 (IQR 2-6). Mean AHI was 0.30 ± 0.3. Mean BMI was 18.62 ± 3.31 kg/m$^2$. Regression analysis demonstrated that increased joint laxity (LLAS) ($p = 0.001$) and an absent anterior articulation of the subtalar joint ($p < 0.000$) were significant predictors of lower AHI (flatter foot). BMI was not. Overall model fit was R2 = 0.3. AHI significantly correlated with OxAFQ_C physical scores (r = 0.34, $p = <0.001$).
*Conclusion*: This is the first study confirming a relationship between subtalar morphology and foot posture. An absent anterior articulation of the subtalar joint places support of the talar head solely on the plantar ligaments. These can stretch, allowing plantar-medial deviation of the talar head with reduction of the medial longitudinal arch seen in FF. This may be exacerbated by increased flexibility observed in this study. Reduced AHI is associated with worse quality of life scores, indicating the presence of symptoms and physical limitations. Altered subtaloid joint morphology should be considered as a potential cause of more severe, symptomatic flat feet. This finding may affect management decisions.
1. Williams (2001) Clin Biomech 16:341–347
2. Shahabpour (1997) Surg Radiol Anat 33(7):623–630

## EP17

### EOS movement artifact after spinal instrumentation: does it affect radiographic measures?

Anne-Laure Simon[1], Emmanuelle Ferrero[2], Brice Ilharreborde[2], Noelle Larson[3]

[1]Robert Debré Hospital, Paris, France
[2]Robert Debre Hospital, Paris, France
[3]Mayo Clinic, Rochester, USA

**LEVEL-IV/Spine**

*Aim*: The EOS® imaging system is an accurate and reliable low dose device, with many advantages for spinal deformity radiography. However, patients must remain motionless for the ten to 15 s during image acquisition. Perturbations are frequently noted on the acquired bi-planar images. The aim of the study was (1) to determine the incidence of the EOS® motion artifact and (2) to assess if motion during EOS imaging acquisition affects common coronal and sagittal plane measurements.
*Methods*: A retrospective study was conducted between January 30, 2013 and June 6, 2014. Inclusion criteria were: (1) previous spinal instrumentation, (2) EOS motion artifact on the antero-posterior and/or the lateral view EOS identified by two orthopedic surgeons on the spinal rods (3) bi-planar conventional radiographs available for comparison. L1–S1 lumbar lordosis, T1 and T4 kyphotic angle, pelvic incidence, distal and proximal Cobb angles and sagittal and coronal T1 spino-pelvic inclination were measured. Means comparison *t* test, Pearson bivariate correlation and intraclass correlation coefficients (ICCs) were used for intraobserver reproducibility and interrater reliability.
*Results*: Both EOS and conventional radiographs were performed for 198 patients with spinal instrumentation. The motion artifact was identified in 16.2 % (n = 32: 16 idiopathic scoliosis, 2 congenital scoliosis, 13 syndromic scoliosis and 1 post-tumoral kyphosis; mean age 15.85 ± 0.5 years) of the cases. Data showed no differences in any of the coronal or sagittal plane measurements between EOS and x-rays. Intraobserver reliability and interrater reproducibility was high, range [0.81–0.98], $p < 0.0001$ except for coronal T1—spino pelvic inclination.
*Conclusions*: EOS radiographs with motion artifact can still be used to produce reliable measurements of the sagittal and coronal angles. Some EOS films with the motion artifact may suggest loss of fixation or bending of the rods. However, after becoming familiar with the appearance of the motion artifact, most repeat radiographs can be avoided.
*Significance*: Motion artifact during full-spine EOS acquisition is frequent but does not affect conventional 2D spinal measurements.

## EP18

### Shoulder joint remodeling after triangular tilt surgery in patients with obstetrical brachial plexus palsy

Firas Suleiman[1]

[1]King Hussein Medical Center, Amman, Jordan

**LEVEL-III/Upper extremity only**

*Purpose*: To study the changes on the shoulder joint by computerized tomography in patients with Obstetrical Brachial Plexus Palsy (OBPP) who underwent triangular tilt surgery.
*Methods*: A retrospective study between 2010 and 2013, on 50 patients with OBPP who underwent triangular tilt surgery, with a follow-up between 1 and 4 years. An axial CT scan was done on all patients before and after surgery to study the changes in the glenoid and the humeral head.
*Results*: All patients who underwent surgery had limitation of external rotation of the shoulder and a positive trumpet sign and the CT scans showed posterior shoulder dislocation, post operative CT scan showed reduced shoulder joints with clinical improvements concerning external rotation although 30 % were still unable to reach their abdomen.
*Conclusion*: Triangular tilt surgery reduces the dislocated shoulder joint and remodeling improves the glenoid dysplasia. Shoulder external rotation increased in all patients. Less than 30 % of the patients may need an internal rotation humeral osteotomy.

## EP19

### Arthroscopic posterior cruciate ligament reconstruction in children: surgical technique and preliminary results

Franck Accadbled[1], Camille Thévenin Lemoine[1], Jérôme Sales de Gauzy[1]

[1]Hôpital des Enfants, Toulouse, France

**LEVEL-IV/Limb reconstructions**

*Purpose*: Posterior cruciate ligament (PCL) tears are rare in the pediatric population. They usually consist of a bony avulsion from the tibia. Intrasubstance ligament tears are exceptional and may cause posterior or postero-lateral instability of the knee. We present an original all arthroscopic reconstruction technique.
*Methods*: Three boys (aged 8, 9 and 11 yo) were operated on between 2009 and 2013 by a single operator. All patients remained symptomatic despite initial immobilization followed by physiotherapy. All arthroscopic PCL reconstruction using a single bundle four strand hamstring autograft was performed an average 6 months after the injury. Posterior tibial shift was fully reducible in all three patients. The femoral tunnel was drilled through the epiphysis in an ouside-inside fashion under fluoroscopic control. The tibial tunnel was transphyseal. The graft was secured using two absorbable interference screws. Postoperative course consisted of long leg cast immobilization for 6 weeks then hinged knee brace for 3 months.
*Results*: At 2 years average follow up (min 12 months), the patients were asymptomatic and resumed sports at the same level as before the injury. On examination, posterior drawer and reverse pivot shift tests were negative. There was no sign of growth disturbance. Stress lateral radiograph showed an average 2 mm differential compared to the unaffected knee. MRI scan confirmed complete integration of the graft and ruled out any meniscal tear or growth disturbance.
*Conclusion/Significance*: PCL injury is extremely rare in children. This original all arthroscopic technique seems appropriate in symptomatic patients with a reducible tibial posterior shift.

## EP20

### The split anterior tibialis tendon transfer procedure for the equinovarus deformity in children with cerebral palsy: predictive factors and outcomes

Noppachart Limpaphayom[1], Phatcharapa Osateerakun[2]

[1]Chulalongkorn University, Bangkok, Thailand
[2]Chulalongkorn University, Bangkok, Thailand

**LEVEL-IV/Foot and ankle**

*Purposes*: The equinovarus deformity causes an unstable posture during gait in children with cerebral palsy (CCP). An overactivity of the anterior tibialis muscle is considered a major contributing factor. Aims of this study were to evaluate predictive factors and outcomes of the split anterior tibialis tendon transfer (SPLATT) procedure for treatment of the spastic equinovarus deformity in CCP.
*Methods*: 45 ambulatory CCP with 68 spastic equinovarus feet positive for the flexor withdrawal reflex test aged 8.1 $\pm$ 2.5 (range 4–15) years were reviewed. All feet underwent a soft tissue release procedure at the ankle joint to correct the equinovarus deformity combined with a transfer of the lateral half of the anterior tibialis tendon to the cuboid using a tendon pull-through technique and were followed for at least 12 months after surgery. The functional outcome was rated using the criteria described by Kling et al. Pre-treatment gross motor functional classification system (GMFCS) levels were compared with those at the most recent evaluations. Factors associated with outcomes and success rate were assessed.
*Results*: The average follow-up time was 5.5 $\pm$ 3.3 years (range 1.1–16), Kling's criteria were classified as excellent in 48 (70 %) and good in 10 (15 %) feet. 10 feet (15 %) were rated as poor owing to overcorrection in three feet and recurrent of the equinovarus deformity in seven feet. However, 43 CCP (96 %) demonstrated an improvement of the GMFCS level, $p < 0.001$ by the Wilcoxon signed rank test. Pre-treatment GMFCS level 3–4 was the only factor predicted poor outcomes with the odds ratio (95 % CI) of 4.92 (0.96–25.2), $p = 0.03$ using the logistic regression analysis. The 10-year success rate of the SPLATT procedure between CCP with GMFCS level 1–2 and level 3–4 were not different with the mean $\pm$ SD (95 % CI) of 0.85 $\pm$ 0.1 (0.5–0.96) vs. 0.6 $\pm$ 0.1 (0.3–0.8), $p = 0.07$, respectively.
*Conclusion*: The SPLATT procedure is simple and effective in maintaining foot position. When combined with correction of the equinovarus deformity, the procedure provides a balanced function of the foot and thus improves ambulation ability in CCP. Pre-treatment GMFCS level 3–4 predicted unfavorable outcomes and should be addressed during pre-operative parental counselling. The procedure should be integrated into the surgical plan for CCP who present with the spastic equinovarus deformity with a positive flexor withdrawal reflex test.

## EP21

### An assessment of the change in growth trajectory over a 50-year period using the multiplier method

James J Aird MBBS, MD, FRCS (Orth)[1], Caroline L Cheesman BSc MBChB MRCS (Eng)[2], Fergal P Monsell MB BCh, MSc, PhD, FRC[2]

[1]Sick Kids, Toronto, Canada
[2]Bristol Roy Hospital Children, Bristol, UK

**LEVEL-II/Limb reconstructions**

*Background*: Paley et al. has developed the multiplier method for predicting leg length. It is a tool that is used clinically to predict leg length discrepancy. The method is also a way of comparing different populations, to identify differences in growth trajectory. This has been done by identifying the differing multipliers for girls and boys. However it has not been used to identify trends in populations separated by time.

Tanner showed that in the first half of the twentieth century girls went from an average age of menarche of 15 in 1900, to 13 in 1970, how this has affected growth trajectory over the last 50 years has not been studied.
*Purpose*: The multiplier method is based on data collected in the 1950's by Anderson and Green, we aim to assess weather there has been a change in growth trajectory between this historical cohort and a contemporary European based cohort.
*Methods*: The Avon Longitudinal Study of Parents and Children of the 90s (ALSPAC) is a longitudinal cohort study of children recruited antenatally, and followed prospectively. Assessment of sub ischial leg length was made on clinical measurements. There were up to 8 measurements per patient, 2,311 girls with a final age at assessment of >15 years and 123 males with a final age of assessment of >16 years were assessed. We used the multiplier method to assess the predicted final leg length and compared this with the actual leg length.
*Result*: There was no difference between the multiplier in girls, in the contemporary cohort and the historical cohort. The average age of menarche in the contemporary group was 12.7. In boys the data is limited, due to later growth and it is not possible to draw comparisons from this group.
*Conclusion*: This data provides good evidence that there has not been a significant change in growth trajectory in girls over the last 50 years.
*Significance*: Implications on the confidence with which we can use the multiplier method in this population.

## EP22

### Does posterior transposition of the origins of the anatomic hip rotators reduce in toeing gait in cerebral palsy?

Mauro Cesar Morais Filho[1], Daniella Lins Neves[1], Francesco Blumetti[1], Cátia Kawamura[1], Marcelo Fujino[1], José Augusto Lopes[1], Leonardo Cortez[1], Simone Bittencourt[1], Fernando Farcetta Júnior[1], Carlos Alberto Santos[1]

[1]AACD, Sao Paulo, Brazil

**LEVEL-IV/Cerebral Palsy**

*Purpose*: The increase of hip internal rotation is the most frequent cause of in toeing gait in cerebral palsy (CP). Femur external rotation osteotomy is a well stablished procedure used for the correction of this problem; however, the recurrence is related in the literature, mainly at young patients. The aim of this study is to evaluate the results of posterior transposition of the origins of the anatomic hip rotators (Majestro–Frost procedure) as an option for treatment of internal rotation of the hip in cerebral palsy.
*Methods*: Retrospective study was performed with consecutive 28 spastic diplegic and hemiplegic CP patients (38 lower limbs), Gross Motor Function Classification System (GMFCS) from I to III who had undergone a correction of internal rotation of the hip through Majestro–Frost procedure (MFP) from October 2008 to December 2012, and with complete documentation at gait laboratory. Clinical and kinematic parameters were analyzed before and after intervention, and a statistical was applied.
*Results*: The majority of patients were spastic diplegic (92.1 %) and the mean age at time of surgery was 117.8 months (9.8 years). Bilateral MFP was done at 10 patients (35.7 %), while 18 (64.3 %) participants received the procedure just at one side. GMFCS level II was more prevalent (63.2 %) and the mean follow-up time was 24.6 months (2.5 years). Regarding the data of physical examination, we observed a reduction of internal hip rotation from 77.5° to 72.9° ($p = 0.011$) and an increase of external hip rotation from 25.2° to 31.9° ($p = 0.001$). At kinematics, there was a reduction of internal hip rotation from 20° to 4.4° ($p < 0.001$) and internal foot progression angle from 15.4° to 1.1° ($p < 0.001$). There was also significant improvement of GDI from 51.2 to 60.9 ($p < 0.001$). The pelvic obliquity at coronal plane was higher at patients with unilateral MFP ($p = 0.023$).
*Conclusion*: In the studied group, MFP was effective in order to reduce internal hip rotation and in toeing during gait in CP patients, however unilateral procedures was related with post-operative pelvic obliquity at coronal plane.
*Significance*: Majestro–Frost procedure can be an option for the treatment of excessive hip internal rotation when the indication for femoral osteotomy is not clear, dynamic problems for instance, or at young patients with high probability of recurrence during bone growth.

## EP23

### Gait improvement surgery in ambulatory children with cerebral palsy: a 5-year follow-up study of 34 children

Terje Terjesen[1], Bjørn Lofterød[2], Ingrid Skaaret[3]

[1]Department of Orthopaedics, Oslo, Norway
[2]Department of Child Neurology, Oslo, Norway
[3]Oslo Movement Laboratory, Oslo, Norway

**LEVEL-III/Cerebral Palsy**

*Background and Purpose*: Previous studies on surgery based on instrumented 3-dimensional gait analysis (GA) in children with cerebral palsy (CP) have shown improved gait function 1 year postoperatively. The aims of this study were to assess the outcome after 5 years, to evaluate the parents' satisfaction with the surgery, and to find the need for additional surgery.
*Patients and Methods*: 34 ambulatory children with spastic diplegia had GA as a preoperative evaluation. Based on this GA, the children underwent a total of 195 orthopaedic procedures on their lower limbs at a mean age of 11.6 (6–19) years. A mean of 5.7 (1–11) procedures per child were performed. Outcome measures were evaluation of gait quality using the Gait Profile Score (GPS) and selected individual kinematics, functional level using the Functional Mobility Scale (FMS), and the degree of parental satisfaction.
*Results*: The mean GPS improved from 20.7° (SD 5.0) preoperatively to 15.4° (SD 4.0) 5 years postoperatively ($p < 0.001$). There was no significant change in GPS between 1 and 5 years. The individual kinematic parameters at the ankle, knee and hip improved significantly. Gait function evaluated by FMS improved significantly, with less need of assistive devices at 5, 50, and 500 m. The mean parental satisfaction, on a scale from 0 to 10, was 7.7 (2–10) points. There was a need for additional surgical procedures in 14 children; this was more frequent in those who had index operation at an early age.
*Interpretation*: The main finding was that orthopaedic surgery based on preoperative GA effected a marked improvement in gait function, that was stable over a 5-year period. Nevertheless, additional orthopaedic procedures were necessary in almost half the children and further follow-up with GA longer than 1 year postoperatively is recommended in children with risk factors for such surgery.

## EP24

### A review of percutaneous Achilles tenotomy in the Ponseti method of treatment of clubfoot

Peter Cundy[1]

[1]Women's and Children's Hospital, Adelaide, Australia

**LEVEL-II/Club-foot**

*Aim*: Percutaneous Achilles tenotomy is required to correct the equinus deformity in approximately 85 % of cases of clubfoot undergoing Ponseti treatment. The purpose of this study was to determine the proportion of initial percutaneous Achilles tenotomies in a South Australian population, compare demographic and clinical factors between patients who did and did not undergo tenotomy and report cases of repeat or delayed tenotomy.
*Methods*: A review was conducted of all patients entered in the Women's and Children's Hospital, Adelaide, prospective Clubfoot Database who underwent Ponseti treatment of idiopathic clubfoot between the years 2005–2013. Physiotherapists graded all patients at each appointment using the Pirani scoring system. A late tenotomy was defined as being performed once abduction bracing had commenced.
*Results*: There were 196 patients (286 feet) with idiopathic clubfoot treated with the Ponseti method between the years of 2005 and 2013. 83.2 % underwent a percutaneous Achilles tenotomy as part of their initial Ponseti management.
Significant associations were found between the initial tenotomy and the number of casts required prior to casting or tenotomy ($p = 0.023$), as well as the hindfoot ($p < 0.0001$) and forefoot scores ($p < 0.0001$) at the commencement of treatment. The median Pirani score at commencement of casting of those undergoing percutaneous tenotomy was higher than those who did not require tenotomy in both the left foot ($p = 0.006$) and the right foot ($p < 0.0001$). 97 % of the feet with a Pirani score >5 at the commencement of casting went on to require initial percutaneous Achilles tenotomy ($p < 0.0001$). The median Pirani score at the end of primary casting prior to any tenotomy was 1 compared with a score of 0 for those who did not undergo tenotomy in both left ($p < 0.0001$) and right ($p < 0.0001$) feet.

Demographics were not significantly associated with initial percutaneous tenotomy. Thirteen patients underwent repeat tenotomy and six patients required late percutaneous tenotomies.
*Conclusions*: An initial Pirani score of >5 strongly predicts the need for tenotomy. Late and repeat tenotomies were rarely performed.

## EP25

### Outcomes of calcaneo-stop procedure in the treatment of juvenile flatfoot in young athletes

Vito Pavone[1], Gianluca Testa[1], Maria Riccioli[1], Giuseppe Caff[1], Giuseppe Sessa[1]

[1]A.O.U.P. Vittorio Emanuele, Catania, Italy

**LEVEL-IV/Foot and ankle**

*Purpose*: The juvenile idiopathic flexible flatfoot is the most common condition treated by a paediatric orthopedic surgeon. Treatment is mainly conservative, but may become surgical in symptomatic patients aged over 8 years with painful feet, fatigue, and clumsiness during and after physical activity. The extended practice of sport in childhood and consequent increase of functional requirements have determined an increase of arthroereisis for the correction of flatfoot. The purpose of this study was to report on the results obtained in children that practiced sports, using the calcaneo-stop procedure.
*Materials and Methods*: A total of 410 feet in 242 patients were treated using the calcaneo-stop procedure from January 1999 to March 2010. All patients followed a rehabilitation protocol avoiding running and jumping for 3 months while swimming was encouraged. Of the entire study group, 70 patients with a mean age of 13 years (range 8–15 years), bilaterally treated, who practiced usual sport activity were considered. A pre-operative and post-operative clinical evaluation using the Foot and Ankle Outcome Score (FAOS), a podoscopic examination and a radiographic assessment with the evaluation of Costa-Bertani angle, at a minimum of 1 year follow-up, were performed.
*Results*: The mean follow-up was 7 years. At clinical evaluation, FAOS System showed an average decrease of 21 % in symptoms and of 34.9 % in pain; daily function was improved by 17 % and quality of life by 52.7 %; sports performance were increased by 60 %. In 12 feet (8.6 %) heel valgus was observed, while the plantar arch was normalized in 128 feet (91.4 %). At radiographic evaluation the Costa-Bertani the angle had a mean decrease of 22.71° ± 6.54.
*Conclusions*: The calcaneo-stop procedure is a simple and minimally invasive procedure that allows early restoration of sport activity in young athletes.
*Significance*: Sport activity restoration came in an average time of 3 months after surgery and showed excellent outcomes, as reported by FAOS System, in which the average score of sport activity at follow-up was 97.1 ± 0.6 %. Success of technique was confirmed by radiographic evaluation, with an average improvement of 22.71° in the Costa-Bertani angle.

## EP26

### Management of high-grade slipped capital femoral epiphysis (SCFE): results of a national survey over a 3-year period

Brice Ilharreborde[1], Vincent Cunin[2], Saad Abu Amara[3]

[1]Robert Debré Hospital, Paris, France
[2]Hospital mère-enfants Lyon, Lyon, France
[3]CHU Rouen, Rouen, France

**LEVEL-III/Hip/Lower extremity**

*Aim*: The management of high-grade slipped capital femoral epiphysis (SCFE) remains challenging and controversial, with a recent trend towards reduction of the deformity. However, the rate of avascular necrosis (AVN) and the risk–benefit of corrective procedures are still unclear. The goal of this study is to report the functional and radiological outcomes of a national multicenter series of high-grade SCFE, treated over a 3-year period.
*Methods*: 186 cases of high-grade (i.e. slip angle >45°) SCFE, stable or unstable, were retrospectively analyzed. The mean follow-up was 2 years. All radiographs were reviewed by three different experienced investigators. The type of procedure was reported and complications rates were assessed. Clinical and radiological outcomes were evaluated, with a minimum 1-year follow-up.
*Results*: According to Loder's criteria, 94 SCFE were stable and 92 unstable. 92 (49.5 %) patients were treated by corrective osteotomy without hip dislocation and 1 with modified Dunn procedure. 43 cases (23 %) were partially reduced preoperatively by external maneuvers and then fixed without open reduction, while 50 patients (27 %) were treated by in situ fixation. Surgery was performed as an emergency (within 6 h after admission) in 4 cases (2 %), and after 24 h in 84 %. The overall AVN rate was 13.5 %. Unstable SCFE had a 3 times higher risk (20.6 vs. 6.3 %), but the delay of surgery did not appear to have a significant influence. 75 % of the cases with hypoperfusion preoperatively developed AVN. The incidence of total or partial AVN was 15 % in the group of corrective osteotomies. No AVN was reported after in situ fixation on stable SCFE. However, radiological femoro-acetabular impingements (FAI) were significantly more frequent in this group, with lower functional scores.
*Conclusion*: Corrective osteotomies through anterior or lateral approaches, without hip dislocation, allow restoration of the proximal femoral anatomy, with a 15 % risk of AVN. The stability of the SCFE is the most important prognostic factor, and perfusion of the hip should be systematically investigated in unstable cases. Radiological FAI are frequent after in situ fixation, but their impact on clinical outcomes need to be further studied.
*Significance*: This large multicenter series of high-grade SCFE show that corrective osteotomies significantly reduce FAI at 2-year follow-up, with a risk of AVN of 8 % in stable hips and 22 % in unstable ones. The higher rate of AVN in unstable SCFE can be partly explained by the post-traumatic preoperative hypoperfusion.

## EP27

### Pediatric ACL injury is associated with increased lateral tibial slope: a case–control study with 152 MRI measurements

David Dare[1], Peter Fabricant[1], Moira McCarthy[1], Brian Rebolledo[1], Daniel Green[1], Frank Cordasco[1], Kristofer Jones[2]

[1]Hospital for Special Surgery, New York, USA
[2]UCLA, Los Angeles, USA

**LEVEL-III/Trauma/Lower extremity**

*Purpose*: Previous research evaluating tibial slope and paediatric ACL injury is limited to date due to small cohort sizes and characterization of the developing proximal tibial chondroepiphysis with plain radiography. The purpose of this case–control study was to use MRI to determine if alterations in posterior tibial slope are associated with ACL rupture in paediatric and adolescent patients as well as to quantify changes in tibial slope by age.
*Methods*: Knee MRI scans were reviewed by three blinded raters in a 1:1 sample of cases and age/gender matched controls. 76 skeletally-immature ACL-injured knees were compared to 76 knees without ACL injury. The mean age was 14.8 ± 1.3 years old. 46 % of both groups were male. Employing a method similar to that used in the adult literature, our technique differed in that the slope was measured at the true joint line (on the cartilage surface) using MRI, not the subchondral bone. Interrater reliability was assured prior to study completion using intraclass correlation coefficient.
*Results*: While the slope of the medial tibial plateau (MTS) was equivalent in the ACL-injured and control knees (5.4° ± 2.2° vs. 5.1° ± 2.3°, $P = 0.42$), the slope of the lateral plateau (LTS) was significantly greater in those with ACL injury (5.7° ± 2.4° vs. 3.4° ± 1.7°, $P < 0.001$). There were no differences in LTS between males and females (4.5° vs. 4.6°, $P = 0.75$). Receiver operating characteristic (ROC) analysis of the LTS revealed that a lateral tibial slope >4° was 76 % sensitive and 75 % specific for predicting ACL rupture in this population. Spearman correlation analysis revealed that medial and lateral tibial slope decreased, or flattened, by 0.18° ($P = 0.028$) and 0.21° ($P = 0.009$) degrees per year, respectively, as adolescents aged.
*Conclusion*: Our data suggests an increase in lateral tibial slope is significantly associated with an increased risk of ACL injury in paediatric and adolescent patients. Posterior slope was also found to decrease, or flatten, with age. A lateral tibial slope >4° was 76 % sensitive and 75 % specific for predicting ACL rupture in this population.
*Significance*: Increased lateral tibial slope, and not medial tibial slope, is significantly associated with an increased risk of ACL injury in pediatric and adolescent patients. This study improves on previous research by using a case–control design, adequately-powered sample size, MRI evaluation of the developing proximal tibial chondroepiphysis, advanced statistical analyses, and assurance of interrater reliability of the measurement method.

## EP28

### French functional method in the treatment of clubfoot 20 years of experience: advantages and limits

Robert Elbaum[1], Brigitte Noel[2], Vincent Degueldere[2]

[1]Clinique Edith Cavell, Bruxelles, Belgium
[2]Centre Hospitalier IRIS Sud, Bruxelles, Belgium

**LEVEL-III/Club-foot**

*Purpose*: Clubfoot treatment has evolved over the last 30 years. Conservative treatment, especially the Ponseti method, has showned the advantage on surgery concerning the immediate correction and the long term follow up. Another conservative method, developed in France since 1950, the Functional Method (FFM) is presented. Because of lack of anglo Saxon literature, this method is not enough diffused in other countries.
*Methods*: Since 1993, 175 children (230 clubfeet) were treated with the FFM in our Institution 210 clubfeet (149 children) were evaluated and reviewed. Only idiopathic clubfeet were included. There were 66 % male and 34 % female. Bilaterality was present in 40 % of the cases. All feet were assessed according to the Dimeglio score. FFM used in our department is based on the "Saint Vincent de Paul" protocol from Paris. (R. Seringe and R. Chedeville). It consists in daily manipulations associated with a system of strapping and plates, the lower limb maintained by thermo formable splints (X-Lite). A night ankle foot orthosis is placed until the age of 5 years. An evaluation at walking age is proposed. After the age of 5, another evaluation is performed according to Ghanem and Seringe score. From 2011, we use Gait analysis to assess long term results.
*Results*: Less than 15 % of the feet, underwent a posteromedial release at walking age (most were type 3 and 4). Compliance was good (more than 90 %). In 15 children the treatment was stopped prematurely (especially during the first years of our experience). At the last follow up, 70 % of the children had good to very good result without major residual deformity. Less than 5 % of the children required a later surgery either for recurrence, or for major residual deformity.
*Conclusion*: FFM is an interesting approach for treating clubfeet, especially concerning rigourous and modularity of splinting system. However, it requires a well-trained physical therapist team and a good collaboration of the parents. Achilleus tenotomy is less systematic in our series and is indicated at 4 months of age when X-ray show the persistence of a high calcaneuswith a tibio calcaneal angle superior to 80°. Long term evaluation encourages us to continue on this way.
*Significance*: FFM represents an alternative method to the Ponseti method for conservative treatment of clubfoot. However comparative study needs to be done, especially regarding the compliance and the cost of those two methods.

## EP29

### Influence of different criteria on severity of clubfoot: a retrospective multicenter study

Robert Elbaum[1], Maxime Bricoteau[2], Brigitte Noel[2], Pierre-Louis Docquier[3]

[1]Clinique Edith Cavell, Bruxelles, Belgium
[2]Centre Hospitalier IRIS Sud, Bruxelles, Belgium
[3]Clinique Universitaire St Luc, Bruxelles, Belgium

**LEVEL-II/Club-foot**

*Objective*: There are currently very few studies describing and analyzing statistically the various criteria by which the idiopathic clubfoot (CCF) is currently caracterised.
The aim of this retrospective study is to analyze these criteria, their influence on each other and finally their influence on the severity of the pathology (Dimeglio score).
*Methods*: A cohort study of 174 children (252 feet) was evaluated in two different Institution. In one hospital, the children were treated with the French Functional Treatment (FFT) and in the other, with the Ponseti method. We analyzed the seasonal nature (CCF births in the two hospitals compared to the Belgian INS data), the prevalence by gender, prematurity, unilateral or bilateral clubfoot (type of injury), severity (Diméglio scoring), the interest of antenatal diagnosis in the future management and finally the genetic aspects of this disease.
*Results*: Population selected for this study was very representative of the Belgian population. No significant seasonal distribution could be shown, however we noticed that the births of our population compared to the Belgian data were centered on the same mean value. The male/female ratio is 2:1. We report 21 % of premature births [IC 95 %: (6 %; 36 %)], 56 % are unilateral clubfeet, a gender influence -male- ($p = 0.0020$), female patients with a bilateral involvement presented a more severe deformation of both feet ($p = 0.005$), a positive antenatal diagnosis when the disease is bilateral ($p < 0.001$). There was a significant heredity factor in our population ($p = 0.021$).
*Conclusion*: CCF affects predominantly boys, who presents more often a bilateral involvement and usually have a grade 3 on the Dimeglio score. The severity can be influenced by gender and by the interaction of gender/type of injury. The hereditary nature of CCF is clearly discernible in our study, specifically among boys (81 %).
The authors also noted the influence of prenatal diagnosis between the 9th and 11th week of pregnancy, on the long term result of the treatment.
*Significance*: Etiology of CCF is multifactorial. The authors pointed the influence of different criteria (gender, type of injury,prematurity…)and the interaction on the severity of CCF.

## EP30

### Outcomes of selective-ultrasound screening for developmental dysplasia of the hip compared to clinical screening alone: a population-based cohort study using a 40-year old historic control group

Fran Sutton[1], Shiela Lee[2], Rachael Hutchinson[1], Anish P. Sanghrajka[1]

[1]The Norfolk and Norwich Hospital, Norwich, UK
[2]Norwich Medical School, Norwich, UK

**LEVEL-III/DDH**

*Purpose*: In 1969, clinical screening of the newborn child for developmental dysplasia of the hip (DDH) was introduced in the United Kingdom (UK). In 1977, a study from our institution was published detailing the incidence of late-diagnosis DDH (defined as age at diagnosis over 3 months) and the rate of surgery for DDH over a 5-year period (1968–1972). Over the last 20 years, "selective ultrasound-screening" (hips scanned if risk factors present or abnormal neonatal examination) for DDH has become well-established in the UK. The purpose of this study was to determine the efficacy of selective ultrasound-screening for DDH in comparison to clinical screening alone, using the incidence of late-diagnosis DDH and the rate of surgery for DDH as outcome measures.
*Methods*: This was a retrospective cohort study performed at a University teaching hospital. The department's neonatal DDH database was interrogated to identify all cases of DDH in newborns during the study period (2008–2013). Our surgical database was used to identify all cases of closed or open reduction for DDH (over the same period). Patients born outside our region, and teratologic cases were excluded from analysis. The Obstetric database was interrogated to ascertain total number of live births over the 5-year period. The resulting data was used to calculate the incidence of late-diagnosis DDH in our region, and the rate of surgery for DDH. These results were compared to the results of the study performed in 1977, using that as the historic control group. Relative Risk (RR) was calculated for the two groups, and analysed for statistical significance.
*Results*: The incidence of late-diagnosis DDH over the recent 5-year study period was 0.67/1,000 live births, compared to 0.6/1,000 in the control group. The RR for late-diagnosis DDH was not significantly different between the two groups (RR 1.14, 95 % CI 0.6–2.2). The rate of surgery for DDH was 0.96/1,000, compared to 0.9/1,000 live births in the control group. The RR for surgery for DDH in the current study population compared to the historic control was 0.97, but this difference was not statistically significant (95 % CI 0.57–1.68; $p = 0.92$).
*Conclusion*: This study shows that despite advances in screening for DDH over the last 40 years, there has been no change in either the incidence of late-diagnosis DDH, or rates of surgery for DDH in our region. Selective ultrasound-screening appears to be no better than clinical screening alone for DDH.

## EP31

### Epsilon-aminocaproic acid does not reduce blood loss associated with periacetabular osteotomy: a retrospective cohort study

Alexander McLawhorn[1], Ashley Levack[1], Kara Fields[1], Evan Sheha[1], Kathryn DelPizzo[1], Ernest Sink[1]

[1]Hospital for Special Surgery, New York, USA

**LEVEL-III/DDH**

*Purpose*: The goal of the periacetabular osteotomy (PAO) is to reorient the acetabular cartilage for acetabular dysplasia. It is a complex series of pelvic osteotomies that has the potential for significant blood loss, for which transfusion rates of up to 50 % have been reported. Therefore it is important to identify effective strategies to manage blood loss and decrease morbidity. Epsilon-aminocaproic acid (EACA) is an anti-fibrinolytic agent known to reduce blood loss for other surgeries. Its efficacy for PAO surgery has not been reported. The purpose of this study was to determine the effects of EACA and other blood management strategies on blood loss after PAO.
*Methods*: 107 consecutive PAOs for acetabular dysplasia were reviewed and dichotomized based on EACA exposure. Demographics, autologous blood pre-donation, anesthetic type, intraoperative estimated blood loss (EBL), cell-saver utilization, and transfusions were recorded. 58 patients received EACA intraoperatively. Total perioperative EBL was calculated. Two-sample t-test, rank-sum test, and Chi square or Fisher's exact tests were used as appropriate. The association between EACA administration and calculated EBL, cell-saver utilization, intraoperative EBL, and maximum difference in postoperative hemoglobin (Hgb) were assessed via multiple regression, adjusting for confounders. Post hoc power analysis demonstrated sufficient power to detect a 250 mL difference in calculated EBL between groups. Alpha level was 0.05 for all tests.
*Results*: Mean perioperative EBL was 1,297 ± 432 mL in the control group (no EACA) and 1,192 ± 354 mL in the EACA group ($p = 0.172$). There was no association between EACA administration and perioperative EBL adjusted for other variables ($p = 0.127$). There were no differences in intraoperative EBL ($p = 0.522$) and cell-saver utilization ($p = 0.665$). There were no differences in preoperative Hgb ($p = 0.142$), Hgb nadir ($p = 0.244$), and maximum Hgb change ($p = 0.788$). Neuraxial anesthesia was most associated with reduction in intraoperative EBL and cell-saver utilization, but had no association with overall perioperative EBL. 4.1 % of controls and 1.72 % of EACA patients received allogeneic blood postoperatively ($p = 0.239$). 40.8 % of controls were transfused autologous units versus 8.6 % of EACA patients ($p < 0.001$). There were no demographic differences between groups.
*Conclusions* EACA administration was not associated with a reduction in any measure of perioperative blood loss in patients receiving PAO. Autologous blood donation resulted in a higher transfusion rate, but had no relationship with blood loss measures.

## EP32

### Demographics and early functional outcomes of combined hip arthroscopic labral repair and periacetabular osteotomy for symptomatic acetabular dysplasia

Benjamin Ricciardi[1], Kara Fields[1], Stephanie Mayer[1], Bryan Kelly[1], Ernest Sink[1]

[1]Hospital for Special Surgery, New York, USA

**LEVEL-III/DDH**

*Purpose*: Labral pathology is common in adolescent and young adult patients presenting with symptomatic acetabular dysplasia. The purpose of this study is to present the demographics and early clinical outcomes associated with combined hip arthroscopic labral repair and periacetabular osteotomy (PAO) for symptomatic acetabular dysplasia.
*Methods*: A retrospective cohort study design was utilized for this study. Patients were enrolled through our prospective hip registry from March 2010 to August 2014. Two groups were utilized: patients undergoing combined hip arthroscopy with labral repair and periacetabular osteotomy (Scope/PAO group) were compared with patients undergoing PAO alone (PAO group). Demographic data was recorded. Pre and postoperative radiographic and functional outcome scores [modified Harris Hip Score (HHS), Hip Outcome Score (HOS), and International Hip Outcome Tool (iHOT-33)] were recorded at 6 months and 1 year follow up. Exclusion criteria included <6 months follow up and bilateral PAO.
*Results*: A total of 57 patients met inclusion criteria (15 patients Scope/PAO, 42 patients PAO). Scope/PAO patients were older relative to PAO alone [29yo (range 15–41 yo) scope/PAO versus 23 yo (range 12–43 yo PAO alone); $p = 0.007$] and had increased previous hip surgery (0 % scope/PAO vs. 31 % PAO). There were no gender differences between the two groups. Labral repair for labral tear was performed in all scope/PAO patients. Mean preoperative and postoperative lateral center edge angle and anterior center edge angle were not different between groups. Mean difference of the change in mHHS adjusted for age was not different between the PAO versus scope/PAO

groups at 6 months [mean difference 95 % confidence interval (CI) − 2 (−13, 9); $p = 0.72$] or 1 year follow up [mean difference (95 % CI): 3 (−13, 19); $p = 0.71$]. HOS Sport subscore showed significant improvements in the scope/PAO versus PAO group at 1 year follow up adjusted for age [mean change (standard deviation): 49 [20] scope/PAO versus 20 [25] PAO; mean difference (95 % CI) 29 [7, 51]; $p = 0.013$]; N = 7, 19 respectively]. No differences were seen in HOS ADL or iHOT quality of life at any time-point. Complications and reoperations were not different between the two groups.
*Conclusion*: Patients undergoing combined hip arthroscopy with labral repair and periacetabular osteotomy are older in age, achieve similar radiographic correction, and had increased improvements in higher-level activity by the HOS Sport subscale relative to PAO alone. Combined hip arthroscopy with labral repair and periacetabular osteotomy is safe and provides significant clinical benefit to appropriately selected patients.

## EP33

### The Aetiology of Thoracic Insufficiency Syndrome in Neuromuscular Scoliosis based on Quantitative Dynamic Lung MRI (QdMRI)

Robert M. Campbell[1], Jayaram Udupa[2], John M. Flynn[1], Oscar H. Mayer[1], Michael Nance[1], Howard Panitch[1], Wei-Hsun Wang[1], Yubing Tong[2], Ivy Wu[2], Keith Baldwin[1], Joseph McDonough[1], Andrew Mong[1]

[1]Children's Hospital of Philadelphia, Philadelphia, USA
[2]University of Pennsylvania, Philadelphia, USA

**LEVEL-III/Spine**

*Introduction*: Neuromuscular (NM) scoliosis is associated with poor pulmonary function felt due to muscle weakness, but thoracic insufficiency syndrome (TIS) due to complex spine/chest deformity also contributes, addressable by surgery, but difficult to assess with current technology. Standard dynamic Lung MRI (dMRI) can only qualitatively show TIS, but we have developed a new quantitative dMRI approach termed QdMRI, and in this pilot study have analyzed the dMRIs of five neuromuscular scoliosis patients. Based on these results, we propose new physiologic mechanisms for TIS in NM scoliosis that appear to improve with treatment.
*Methods*: Retrospectively the records, xrays, and pre-op dMRI scans of five NM scoliosis pts, three post op scans. All pts were treated with bilat rib to pelvis hybrid VEPTRs (Eiffel Tower). QdMRI analysis was by constructing a consistent 4D image from the free-breathing dMRI slice acquisitions, segmenting key anatomic structures in the 4D volume, and deriving quantitative dynamic parameters from the defined structures.
*Results*: Avg age 7.9 years, avg f/u 2.13 years. Scoliosis 75° preop, 45° at f/u. SAL .84 preop, .96 at f/u. Pelvic obliquity 19.6°, f/u 13.6° One pt had substantial soft tissue remodeling of the kidney with increased excursion on dMRI. On QdMRI, preop concave ?rib cage volume (cc) was 46.5, ?diaphragm volume 25.7, convex ?rcv 40.5, ?dv 27.4. Kidney excursion (mm) concave was 2.58, convex 3.02. Two pts had concave ?dv < convex, one concave > convex, two had equal volumes.
For the three patients with post op scans, concave ?rib cage volume increased 57 % (convex 72 %), ?diaphragm volume increased 128 % (convex 109 %). Concave kidney excursion increased 6.3 mm (320 %), convex 5.2 mm (192 %).
Complications included two pts with migration of devices proximally.
*Conclusions*: VEPTR treatment appears to increase rib cage expansion more on the convex side of the thorax than the concave, and conversely increases diaphragm excursion on the concave side. With treatment, renal excursion increases bilaterally, concave > convex, probably reducing a blockade effect on the diaphragm.
*Significance*: QdMRI can measure separate contributions of diaphragm and rib cage to lung expansion during respiration for each hemi-thorax, enabling accurate assessment of thoracic function for the first time. QdMRI has potential to increase the understanding of the anatomic mechanisms of spine/chest wall deformity that cause restrictive lung disease in NM scoliosis and may enable development of new treatments for it.

## EP34

### Ingrowing toenails in children and adolescents: is operative treatment superior to non-operative treatment?

Sumanth Gera[1], DK Sa'diah PG Zaini[1], Siti Hauzah Abdul Rahaman[1], Kevin Lim[2]

[1]KK Hospital, Singapore, Singapore
[2]KK Hospital, Singapore, Singapore

**LEVEL-IV/Other/varia**

*Purpose*: To compare the results of non-operative treatment with that of operative treatment in the management of ingrowing toenails (IGTN) in children and adolescents.
*Methods*: All children and adolescents who were evaluated for IGTN in our institution over a 4-year period (2007–2010 inclusive) were included for study; clinical charts were reviewed retrospectively. Demographic data, treatment prescribed and outcome at 6 months after presentation were recorded.
*Results*: 151 patients were recruited. There were 80 males (53.0 %) and 71 females (47.0 %); the median age was 13.8 years (range 0.43–16.0). 77 of 151 toes (60.6 %) were treated non-operatively with nail care advice, topical antibiotics and daily cleansing. 74 toes (49.0 %) were treated surgically; in this operative group, 48 of 74 (64.9 %) underwent wedge resections without matrix ablation while the remaining 26 (35.1 %) had total nail avulsions without matrix ablation At 6 months follow up, there were 7 (9.1 %) recurrences in the non-operative group and 13 (17.6 %) recurrences in the operative group; this difference was not statistically significant.
*Conclusion*: In this series, there is a lower recurrence rate for IGTN treated non-operatively compared with those treated operatively without matrix ablation. However, the difference between the groups is not statistically significant.
*Significance*: Index presentations of IGTN in children and adolescents can be managed non-operatively in the first instance. Meticulous nail care (without aggressive nail trimming) and cleansing should the mainstay of treatment. Operative options should be reserved for recurrent cases, and should probably include at least a partial germinal matrix ablation.

## EP35

### The effect of alendronate-loaded polycarprolactone scaffolds for the enhancement of osteogenic differentiation in vitro and bone regeneration in vivo

Kwang-Won Park[1], Ji-Ho Lee[1], Han-Ju Kim[1], Hae-Ryong Song[1]

[1]Korea University Guro Hospital, Seoul, Republic of Korea

**LEVEL-I/Basic science**

*Purpose*: The purpose of the study was to investigate the effect of alendronate (Aln)-loaded polycarprolactone (PCL) scaffolds for the osteogenic differentiation in vitro and bone formation in vivo.
*Methods*: Aln/PCL scaffolds were prepared by using a rapid prototyping system. The characterizations of Aln/PCL were evaluated by scanning electron microscopy (SEM) and thermogravimetric analysis (TGA). The osteogenic differentiation of MG-63 cells on Aln/PCL was evaluated by examining alkaline phosphatase (ALP) activity and calcium content in vitro.
*Results*: The Aln/PCL combination showed significantly more ALP activity and mineralization than the PCL alone. The capacity of Aln/PCL to regenerate new bone was determined in a rat tibia defect model in vivo. In vivo study was evaluated by radiography (X-ray), micro-computed tomography (micro-CT), and histological analysis. At 8 weeks after implantation, Aln/PCL had a positive effect on bone formation as judged by the results of X-ray, micro-CT, and histological analysis.
*Conclusion*: These results suggested that Aln/PCL scaffolds enhanced the osteogenic differentiation in vitro as well as bone formation in vivo compared with PCL scaffolds.

## EP36

### Normalization of acetabular index in infant with hip instability (without subluxation or dislocation): natural history with minimum 3 years follow-up

Blazej Pruszczynski[1], H. Theodore Harcke[2], Laurence Holmes, Jr.[2], J. Richard Bowen[2]

[1]Medical University of Lodz, Lodz, Poland
[2]Nemours AI duPont Hospital, Wilmington, DE, USA

**LEVEL-II/DDH**

*Purpose*: The natural history of hip instability (without subluxation or dislocation) and treatment in infants remain controversial. We performed a retrospective cohort case-only study with blinded, prospectively collected data to assess normalization of the acetabular index in consecutive untreated infant hips with sonographic instability.
*Methods*: Consecutive hips meeting inclusion criteria were followed by sonography and radiography, data were analyzed using tabular and regression models.
*Results*: In 48 hips, acetabular index (AI) measured by radiography normalized within 3 years of age without treatment. Age at normalization of AI occurred as follows: by 7 months in 35 %, 12 months in 67 %, 18 months in 75 %, 24 months in 81 %, and 36 months in 100 %. Two patterns of normalization of the acetabular index were observed: group I showed ossification in a physiological range of normal by 7 months of age, and group II had delayed ossification with later normalization of the acetabular index measurement. Breech presentation ($p = 0.013$) and cesarean delivery ($p = 0.004$) correlated statistically directly with a later normalization.
*Conclusions*: The natural history of infant hip instability (without subluxation or dislocation), which is reduced at rest and unstable with stress as diagnosed by the Harcke method of sonography, has spontaneous normalization of the acetabular index within 3 years of age. We suggest three patterns of acetabular ossification in unstable infants' hips: (1) normal ossification, (2) delayed ossification with normalization of the acetabular index by age 3 years, and (3) defective secondary centers of ossification with an upward tilt of the lateral acetabular rim in adolescence.

## EP37

### Natural history of pediatric elbow dislocation: functional and radiological evaluation in the adult

Elena Galvez[1], Luis Moraleda[1], Samuel Antuña[1]

[1]Hospital Universitario La Paz, Madrid, Spain

**LEVEL-II/Trauma/Upper extremity**

*Purpose*: The purpose of this study is to evaluate the long term clinical and radiological outcome of an elbow dislocation during childhood.
*Methods*: 12 patients who sustained an elbow dislocation during childhood were reviewed at an average follow-up of 13 years. Data regarding age at the time of dislocation, sex, side involved, hand dominance, associated fractures, time of immobilization and complications were recorded. Patients returned for clinical evaluation that included range of motion, clinical carrying angle and stability. Clinical outcomes were described according to the Mayo Elbow Performance Score (MEPS). Patients completed the quick-DASH questionnaire to measure disability. Radiographs of both elbows at the time of follow-up were evaluated to look for osteoarthritic changes.
*Results*: The mean age at the time of fracture was 10.2 years and at the latest follow-up was 23 years. There were no new episodes of elbow dislocations. There were significant differences between injured and uninjured elbow at follow-up regarding flexion–extension (injured: $135° \pm 8.4°$, uninjured: $141° \pm 5.5°$, $p = 0.024$), pronation (injured: $75.4° \pm 6.6°$, uninjured: $80° \pm 0°$, $p = 0.034$), and pronosupination (injured: $154.6° \pm 6.6°$, uninjured: $160° \pm 0°$, $p = 0.020$). The average score was 0 for the QuickDASH questionnaire and $98.7 \pm 4.3$ for the MEPS. No elbow instability was present. A 'non-perfect result' was found in three patients and was not associated with age at the time of dislocation ($p = 0.237$), age at the latest follow-up ($p = 237$), follow-up ($p = 0.310$), time of immobilization ($p = 0.552$) or presence of an epitroclear fracture ($p = 0.1$).
*Conclusion*: Adult patients who sustained an elbow dislocation during childhood present excellent functional results. Elbow instability or degenerative changes are not present.

## EP38

### How long should the Pavlik harness be continued?: a study of acetabular development during Pavlik harness treatment in DDH

Kenjiro Wakabayashi[1], Ikuo Wada[1], Eisuke Sakuma[1], Kinya Ito[1], Kazuki Hattori[1], Yasuhiro Shirai[1], Myong Su Ha[1], Takanobu Otsuka[1]

[1]Nagoya City University, Nagoya, Japan

**LEVEL-III/DDH**

*Purpose*: The Pavlik harness is the standard treatment for developmental dysplasia of the hip (DDH), but there is a lack of consensus on how long the harness should be continued. The purpose of this study was to identify a new indicator for determining the duration of the Pavlik harness treatment.
*Methods*: The study targeted 49 hips in 49 patients (4 male and 45 female) with unilateral DDH treated with the Pavlik harness. Plain X-ray was used to measure the acetabular index before and after harness treatment. The patients were divided into two groups. Group 1 (32 hips) were Severin classification type I or II at final examination. Group 2 (17 hips) required surgery to correct residual subluxation, or were Severin classification type III or IV at final examination. The mean age at final examination was 13 years 9 months old. As a control group, we researched the unilateral limited hip abduction patients who were not DDH. In the control group consisting of 42 hips of 42 patients, the acetabular index was measured at the same age as the harness treatment group.
*Results*: Mean decrement of the acetabular index during harness treatment was 7.6° ± 4.6°, which differed significantly from mean decrement of the acetabular index (1.0° ± 1.5°) in the control group ($p < 0.001$). Mean acetabular index at harness removal in group 1 was 28.7° ± 3.0°, which differed significantly from the mean acetabular index (33.2° ± 3.0°) at harness removal in group 2 ($p < 0.001$). As a result of having calculated in receiver operating characteristic analysis, the boundary between group 1 and group 2 of the acetabular index at the Pavlik harness removal was deemed to be 31°.
*Conclusions*: There was remarkable acetabular development during harness treatment. Patients with the acetabular index ≤31° at the Pavlik harness removal subsequently showed good acetabular development, whereas patients with the acetabular index >31° at harness removal subsequently showed poor acetabular development. These findings suggest that the Pavlik harness should be continued until the acetabular index improves to ≤31°.

## EP39

### Preoperative treatment with pamidronate for the structural and functional state of osseous tissue in children with osteogenesis imperfecta

Iurii 1. Guk[1], Andrii 2. Zyma[1], David Stewart[2], Tamara Kinchaya-Polishchuk[1], Stepan Martsynyak[1], Andriy Cheverda[1]

[1]Institute of Orthopedics, Kyiv, Ukraine
[2]Childrens Bone and Spine Surge, Las-Vegas, USA

**LEVEL-IV/Metabolic**

*Purpose*: The disordered structural and functional state of bone in osteogenesis imperfecta complicates surgical interventions (thin cortex, difficulty preparing canal for intramedullary rod). We studied the impact of preoperative pamidronate on surgical outcomes in patients with osteogenesis imperfecta.
*Methods*: Retrospective analysis of 12 patients with osteogenesis imperfecta (Sillence type I in 5 patients, III type in 7 patients) who did not ambulate without assistance (8 females and 4 males). We assessed structural and functional state of bone by biochemical study of bone metabolism and DEXA of lumbar spine with subsequent preoperative treatment of metabolic alterations that included administration of calcium supplements (500–800 mg/day), active forms of vitamin D (0.25–1 μg/day) and pamidronate (0.5–1.0 mg/kg/day provided that serum ?? was above 2.4 mmol/L). Pharmacological preparation included three cycles separated by 3 months intervals. The number of infusions was determined by the level of disturbance of bone density and metabolism. Therapeutic efficacy was assessed by the decrease of β-??? and increase of Z-factor. Subsequently the patients underwent surgical interventions for correction of long bone axial deformities with intramedullary telescopic rods.
*Results*: Average age on treatment was 8.5 years (range 3–15 years), with 2-year postoperative follow-up. To evaluate the osseous tissue's condition preoperatively we checked level of serum osteocalcin (118.14 ± 63.61 ng/ml), total P1NP (342.07 ± 293.14 ng/ml), β-??? (1.24 ± 0.68 pg/ml), and DEXA Z-score (−4.64 ± 1.14). Analysis of the data received showed that after pamidronate treatment, the rate of bony resorption diminished on average by 25 % (β-???—0.93 ± 0.388 pg/ml, $p = 0.026$); Z-score increased by 12 % (−3.9 ± 1.4; p = 0.04). We noted that the lowering of serum ?? by 0.2–0.3 mmol/l during administration of pamidronate comparing to the baseline parameters was eliminated with calcium supplementation and vitamin D. Visually during the operative intervention we noted the thickening of cortical layer and increase of bony strength. All operative patients experienced timely union of osteotomies. One patient developed a femoral fracture with a rod in situ after the onset of independent ambulation that was effectively manged witha plaster cast. 8 patients became community ambulators without support, whereas the remaining 4 patients are at the last stage of surgical intervention.
*Conclusion*: Preoperative treatment with pamidronate and calcium in patients with osteogenesis impefecta improves bone quality and does not inhibit bony union after osteotomy.
*Significance*: The above-mentioned therapy programme leads to improved technical conditions during implantation of telescopic rods in children with osteogenesis imperfecta.

## EP40

### Two year evaluation of use of tranexamic acid and its efficacy in paediatric hip reconstruction surgery: retrospective case series

Ibidumo Igah[1], Ebraheim Izadi[1], Claudia Maizen[1]

[1]Royal London Hospital, London, UK

**LEVEL-III/Cerebral Palsy**

*Background*: Tranexamic acid has been shown to reduce mortality in patients with or at risk of significant haemorrhage following trauma in high quality randomized control trials. It has also been shown to have efficacy in paediatric craniosynostosis surgery. However there is no current evidence showing efficacy in major paediatric orthopaedic surgery.
*Methods*: Retrospective study of all patients who had hip reconstruction surgery done within a 2 years period. Data of use of tranexamic acid, amount of blood transfused (ml), blood loss (ml), pre-operative and post-operative haemoglobin (mg/dl) level was collected.
*Results*: There were 37 patients who had hip reconstruction within the 2 years period and all patients were included in this study. Age range was 2–15 years with average age of 8 years. The procedures performed were bilateral pelvic osteotomy and femoral osteotomy (8), bilateral femoral osteotomy and unilateral pelvic osteotomy (4), bilateral femoral osteotomy (3), unilateral femoral and pelvic osteotomy (13), unilateral pelvic osteotomy (4), unilateral femoral osteotomy (4) and bilateral pelvic osteotomy (2). 14 of 37 patients that had hip reconstruction surgery had tranexamic acid during their procedure. Tranexamic acid was used in more complex hip reconstruction surgery; bilateral femoral and pelvic osteotomy (90 %) and unilateral femoral and pelvic osteotomy (38 %) and 7 of these patients required intra-operative blood transfusion. 4 patients out of the 13 patients in the group who had no tranexamic acid but less complex hip reconstruction were transfused intra-operatively.
All patients had a decrease in haemoglobin 24 h post operatively. There was no significant difference in decrease in haemoglobin level 24 h post operatively between patient who had intra-operative blood transfusion with or without use of tranexamic acid. However, there was significant difference in decrease in haemoglobin level 24 h post-operative in patients who had no transfusion with use tranexamic acid against those who had no tranexamic acid (2.0 mg/dl vs. 3.3 mg/dl; $p < 0.05$).
*Conclusion*: This study suggests tranexamic acid has efficacy in paediatric hip reconstruction surgery in reducing blood loss. A larger designed randomized controlled trial would be required in future to clearly demonstrate this results and determine perioperative tranexamic acid administration guidelines.

## EP41

### Is percutaneous gracilis tenotomy as effective and safe as the open procedure?

Bilal Hachache[1], Tony Eid[1], Elias Ghosn[1], Amer Sebaaly[1], Khalil Kharrat[1], Ayman Assi[2], Ismat Ghanem[1]

[1]Hotel-Dieu de France Hospital, Beirut, Lebanon
[2]School of Medicine USJ, Beirut, Lebanon

**LEVEL-II/Cerebral Palsy**

*Introduction*: The purpose of this prospective study was to compare the effectiveness and safety of percutaneous gracilis tenotomy (PGT) with those of the open precudre (OGT).
*Methods*: A cross-over randomized controlled trial was conducted on 59 consecutive hips in 31 CP patients scheduled for gracilis tenotomy in the setting of multilevel procedures or hip surgery. A pediatric orthopaedic surgeon conducted a PGT. Another surgeon extended the wound to explore what had been cut during the PGT, and completed the tenotomy if necessary. Hip abduction (HA) was assessed by a third surgeon immediately before PGT, after PGT, and then after OGT, using a goniometer, in a standardized reproducible manner. Primary end-points included the percentage of muscle portion sectioned percutaneously (%MPSP), and the HA measure. Comparison between HA before and after PGT and between HA after PGT and OGT was done. Bleeding and the iatrogenic lesions were identified. Relation between HA after PGT and %MPSP was evaluated.
*Results*: Mean HA measured 33.71° preoperatively and increased to 45.90° after PGT. After OGT, HA averaged 48.71° with no statistical gain compared with that observed after PGT. Muscular portion of gracilis origin was cut to an average of 91.95 %. Gain in HA did not correlate with the extent of the muscular portion sectioned percutaneously ($R = -0.043$). Partial section of adductor brevis after PGT was encountered in 39 cases. Considerable bleeding with hematoma formation requiring hemostasis during the open control procedure occurred in 30 hips. Partial iatrogenic injury of the anterior branch of the obturator nerve was encountered in one patient bilaterally with very severe adductor contracture, due to an anatomic variant of the branch localization (too medial).
*Conclusions*: This is the only prospective study concerning the effects of PGT. The authors detail the technique of PGT and show that although PGT is a fast, simple and effective procedure, it is not as safe as the open release even when done correctly by an experienced surgeon, mainly because of the increased risk of bleeding.

## EP42

### Subscapularis release in the treatment of shoulder adduction and internal rotation contracture in children with Erb’s palsy

Elias Naoum[1], Elie Saghbini[1], Ayman Assi[2], Ismat Ghanem[1]

[1]Hotel-Dieu de France Hospital, Beirut, Lebanon
[2]School of Medicine USJ, Beirut, Lebanon

**LEVEL-III/Trauma/Upper extremity**

*Purpose*: This paper evaluates the results on shoulder function of an isolated proximal subscapularis release in children with Erb’s palsy.
*Methods*: A retrospective study was conducted on 64 consecutive children with Erb’s palsy who underwent a Carlioz proximal subscapularis release between 2001 and 2012. Fifty-one children with complete records and a minimum follow-up of 2 years were included for evaluation. Age at surgery ranged from 1.3 to 4.5 years (average 2.6 years). Preoperative passive and/or active (depending on age at surgery) shoulder abduction/anterior elevation (ABD), external and internal rotations (ER–IR) as well as the Mallet score were compared with those found at 6 and 24 months postoperatively using the «student paired *t* test», with a confidence interval of 95 %. The results were compared between children <3 years of age at surgery and those older, and between children who had an isolated C5, C6 and those with greater involvement. A $p < 0.05$ was considered as statistically significant.
*Results*: Abduction improved 21° at 6 m and 31° (total) at 2 years ($p < 0.01$) with an overall Mallet abduction score improvement of 0.58 at 6 m and 0.6 (overall) at 2 years ($p < 0.01$). External rotation improved 52° at 6 m and 35° (total) at 2 years ($p < 0.01$) with an overall Mallet external rotation score improvement of 1.3 at 6 m ($p < 0.01$) and 0.52 (overall) at 2 years ($p = 0.013$). There was no statistically significant improvement in internal rotation ($p = 0.37$). We found no correlation between the child’s age or the severity of involvement at surgery and the end result.
*Conclusion*: Proximal subscapularis release according to Carlioz is simple and effective in improving overall shoulder function in children with obstetrical brachial plexus palsy, mainly abduction and external rotation. Improvement tends to reach a plateau around 6–12 months postoperatively. The improvement of shoulder internal rotation remains the most difficult goal to achieve in these children.

## EP43

### We need another alternative for the treatment of aneurysmal bone cysts: preliminary results of sclerotherapy using Surgiflo

Ismat Ghanem[1], Nicolas Nicolas[1], Ayman Assi[2], Gaby Kreichaty[1], Sami Slaba[1]

[1]Hotel-Dieu de France Hospital, Beirut, Lebanon
[2]School of Medicine USJ, Beirut, Lebanon

**LEVEL-II/Tumours**

*Introduction*: Aneurysmal bone cysts (ABC) are benign bone lesions that can be locally aggressive causing bone destruction and pathological fracture. Traditional treatments consist of surgery (curettage or excision), and/or intralesional sclerotherapy. Ethibloc was one of the most effective fibrosing agents but was withdrawn because of reported local and general complications. The purpose of this preliminary study was to evaluate the results of a percutaneous haemostatic agent Sugiflo (Ethicon) in the treatment of active and aggressive ABC.
*Methods*: Thirteen consecutive patients with ABC were treated in our institution between December 2009 and June 2013, at an average age of 10.1 years (3.2–17.4) by percutaneous intracystic administration of a mixture of a haemostatic agent Surgiflo and pure alcohol. The cysts were located in the proximal humerus (5), proximal tibia (3), distal femur (2), iliac bone (1), L3 (1), and C4 (1). Six patients were previously treated with surgical curettage (3), internal fixation (2) or surgical excision (1). The mixture was administered under fluoroscopic and/or computed tomographic guidance under local, regional or general anesthesia. Patients were assessed clinically and radiologically at an average follow-up of 16 months (6–42). The number of required procedures per patient, the improvement of pain, rate of pathologic fracture if any, tumor volume, distance to the physis, as well as improvement in cortical thickness were assessed. Complications and recurrences were recorded.
*Results*: The procedure was performed once in 12 patients, and twice in 1 patient. At last follow-up, 12 patients were pain free. One patient with a very aggressive ABC of C4 developed neurologic symptoms 2 months following the procedure due to vertebral collapse and cord compression and required surgical resection and cervical fusion. There was a decrease in mean tumor volume by 13 %, an increase in mean distance between physis and tumor of 7.9 mm, and in mean cortical bone thickness of 2.5 mm. The complications were minor and included postoperative pain for 3 weeks (1 case) and slight limb shortening (3 cases).
*Conclusion*: Percutaneous administration of Surgiflo for active and aggressive ABCs seems to be effective and safe and shows promising results. It is an easier alternative to surgery mainly in some specific locations for which surgery may be associated with a high morbidity.

## EP44

### Is an age less than 16 years a valid “red-flag” for low back pain? An evaluation based upon positive magnetic resonance imaging findings

Joanna Thomas[1], Julekha Girach[2], Kate Armon[1], Rachael Hutchinson[1], Anish Sanghrajka[1]

[1]Norfolk and Norwich Hospitals, Norwich, UK
[2]Norwich Medical School, UEA, Norwich, UK

**LEVEL-IV/Spine**

*Introduction*: Low back pain whilst very common in the adult population, is still regarded as unusual in the paediatric population. Consequently, when managing back pain, patient age <16 years is regarded as a “Red Flag”, in which investigation should be performed to exclude a significant pathology such as infection or tumour.
*Aim*: The purpose of this study was to investigate whether patient age <16 years is a valid “red flag” for back pain, by determining how often magnetic resonance imaging (MRI) investigations in these patients demonstrated significant pathology.
*Methods*: This was a retrospective case review performed at a University teaching hospital. Inclusion criteria for this study were patients under the age of 16 having an MRI scan of the lumbar spine for a primary complaint of low back pain. Emergency admissions were excluded from the study. The radiology department’s database was interrogated to identify all patients under the age of 16 who had undergone an MRI scan of their lumbar spine during the study period (2008–2012). All emergency and inpatient admissions were excluded from the study. Casenotes of each of these patients were analysed for demographics and features of the history, examination and management.
*Results*: 98 eligible cases were identified over the study period. The age range of these patients was 2–16 years (mean age 12.63 years). The scan found no abnormalities in 71.4 % of cases. Tumour or infection were found in 3 % of cases, (2 cases sacroilitis, 1 sacral chondroblastoma). There had been sacral or sacroiliac tenderness in each of these cases. The other positive findings were 8 cases of spondylolysis, 3 spondylolistheses, 9 cases of disc degeneration and 5 cases of Scheuermann’s.
*Conclusion*: This study shows that whilst the diagnostic yield of MRI in patients under the age of 16 with low back pain is relatively high (28.6 %), in the vast majority of cases (97 %) it did not significantly alter management. Sinister pathology (infection or tumour) was found in only 3 % of cases. We therefore suggest that an age of <16 years, in isolation, should no longer be a red flag indicator for low back pain. We do however advise a lower threshold for imaging in patients presenting with sacral region pain and tenderness.

## EP45

### Rebound growth after hemiepiphysiodesis

Ignacio Sanpera[1], Laura Corominas[2], Guillem Frontera-Juan[2], Carles Saus-Sarrias[2]

[1]Hospital SonEspases, Palma de Mallorca, Spain
[2]Hospital Son Espases, Palma de Mallorca, Spain

**LEVEL-I/Basic science**

*Purpose*: Rebound growth after hemiepiphysiodesis may be a normal event, but little is known about its causes, incidence or factors related to its severity. The aim of this study was to evaluate rebound growth under controlled experimental conditions.
*Methods*: Twenty-two rabbits were submitted to medial proximal tibial hemiepiphysiodesis using a 2-hole plate and screws. Temporal arrest was maintained during 3 weeks, and animals were sacrificed at intervals ranging between 3 days and 3 weeks after the device removal. The radiological angulation of the proximal tibia was studied at weekly intervals during and after hemiepiphysiodesis. At sacrifice a histological study of the tibial proximal physis was performed.
*Results*: The mean angulation achieved at 3 weeks was 34.5° ± 3.36°, and remained unchanged for the studied period of up to 2 weeks. At 3 weeks time, the mean angulation dropped to 28.09 ± 1.79 ($p > 0.001$). Histologically, a widening of the medial side was noted during the first 2 weeks.
*Conclusion*: Rebound growth is an event of variable incidence and intensity and when present does not appear immediately after growth restoration, but may take some time to appear.
*Significance*: This work contributes to the understanding of the rebound growth observed after removal of hemiepiphysiodesis implant.

## EP46

### Trends in the operative treatment of femoral fractures in children and adolescents

Justine St George[1], Christopher Phoon[1], David Little[1]

[1]CHW, Sydney, Australia

**LEVEL-III/Trauma/Lower extremity**

*Background*: Femoral shaft fractures are a common injury in children and adolescents and debate still exists regarding the safety of Trochanteric Anterograde Nails (TAN) fixation in this population. Increased deformity has also been noted with the use of Titanium Elastic Nails (TENs) in children over 50 kg. At our institution there has been increasing surgeon preference for TAN fixation. The objectives of this study were to note evolving trends in our practice and evaluate the outcomes comparing each method of intramedullary fixation.
*Methods*: A retrospective review was performed of all femoral fractures treated at The Children's Hospital at Westmead from November 1998 to June 2013. All pathological fractures were excluded.
*Results*: The study population consisted of 148 patients with 150 femoral shaft fractures, 95 of which underwent TENs fixation, and 55 of which underwent TAN fixation. Patients were predominantly male (77 %) with a mean age was 12.2 (7–15) years for TAN and 8.4 (4–13) years for TENs fixation. The vast majority were closed (97.4 %) and resulted from high energy injuries (57.3 %). Most of the TAN cases were performed in the last half of the 15 years study period.
Malunion was defined as more than 10° of angulation in the coronal or sagittal plane. No significant difference in the resulting deformity was found between the two groups, with 0 % of TAN and 4.4 % of TENs resulting in coronal plane; and 7.3 % of TAN and 14.7 % of TENs resulting in sagittal plane deformity ($p = 0.29$ and $p = 0.20$ respectively). Rotational deformity was found in 3.6 and 3.2 % of TAN and TENs fixation respectively and corrective osteotomy was required in 1.8 % of TAN and 3.2 % of TENs fixation.

A clinically significant limb length discrepancy (≥2 cm) occurred only following of TENs fixation in 3.2 % of cases. All required epiphyseodesis.

There were no cases of avascular necrosis of the femoral head.
*Conclusion*: There has been a trend at our institution to increasing use trochanteric entry nails without an increase in complications.

## EP47

### Oral risedronate as treatment of osteogenesis imperfecta

Nagy Sabet[1]

[1]Faculty of Medicine M.U.S.T., Cairo, Egypt

**LEVEL-I/Other/varia**

*Purpose*: Osteogenesis Imperfecta (OI) is a heritable disorder characterized by either a reduction in the production of normal type I collagen or the synthesis of abnormal collagen as a result of mutations in the type I collagen genes.
Affected patients tend to have fragility fractures from the mildest trauma and progressive skeletal deformities.There is no effective medical treatment for OI. Bisphosphonates are synthetic analogues of pyrophosphate that inhibit bone resorption by their action on osteoclasts.

Recently, beneficial effects of intravenous pamidronate treatment are reported in OI. However, this treatment requires frequent hospital admissions and is relatively expensive. Risedronate is an oral bisphosphonates effectively used in adults with osteoporosis. This study aimed to evaluate the efficacy of oral risedronate in children with OI.
*Methods*: Between 2006 and 2009, 23 patients with OI were referred for bisphosphonate therapy. The OI was classified using the Sillence criteria. All patients underwent baseline biochemistry, radiographic studies and bone mineral density (BMD) measurements before commencing therapy. Patients were commenced on oral risedronate (5 mg alternate days in patients below 6 years and every day for the others) along with calcium (500 mg/day) and followed up for a period 2–4 years. Five cases underwent surgery to correct deformities and/or fracture fixation Serum calcium (Ca), phosphorus (P), alkaline phosphates (ALP), and urinary Ca/Cr ratio were studied 6 monthly and BMD by DXA on 6–12 monthly basis.
*Results*: After treatment, the fracture rate decreased significantly, and bone density improved in each individual case. Serum Ca, P, ALP, and urinary Ca/Cr did not change significantly during treatment. All QOL markers showed significant improvement in response to risedronate therapy.
*Conclusion*: Risedronate is effective, safe and a practical alternative to intravenous bisphosphonates in treatment of children with OI.

## EP48

### $O_2$ cost and walking speed in youth with cerebral palsy

Julieanne Sees[1]

[1]Nemours Hospital for Children, Wilmington, USA

**LEVEL-I/Cerebral Palsy**

*Background/Objectives*: Oxygen ($O_2$) cost (ml$O_2$/m) represents gait economy and is derived by dividing oxygen uptake (ml/kg/min) during gait by walking speed (m/min). In normal gait it is stable over a modest range of speeds. We know gait in youth with cerebral palsy (CP) is associated with high $O_2$ cost, but we do not know whether $O_2$ cost is stable across speeds in this group. Improved economy is a common goal of gait surgery and achieving it requires knowledge of how walking speed and $O_2$ cost interact for youth with CP. The purpose of this study was to examine the stability of $O_2$ cost over a range of self-selected walking speeds in youth with CP.
*Participants and Setting*: A convenience sample of youth with CP, GMFCS Levels I–III, ages 6–18 years, without recent orthopedic surgery or Botox injections from two pediatric hospitals.
*Materials and Methods*: Youth with CP at GMFCS Level I (28), II (16) and III (13) mean age 12.6 (SD = 3.3) participated in a single test session performing a range of physical activity (PA) tasks while wearing the Cosmed k4b2. During three 6-min walk tasks, youth walked at a slow, brisk, and fast pace. Mean VO2 from the middle 2-min of each walk was divided by velocity (d/t) to calculate $O_2$ cost. ANOVA was used to analyze differences in walking speed and $O_2$ cost between the three walking tasks.
*Results*: 53 participants had complete VO2 and speed data. On average, youth had a 61 % change in walking speed from slow to fast. Differences in speed between tasks are significant for all comparisons ($p < .001$). No significant differences exist for $O_2$ cost between speeds.
*Conclusions/Significance*: Self-paced walking speeds and $O_2$ costs varied widely among youth in this study who were able to vary speed by more than 60 % from slow to fast. Changes in walking speed, however, produced no significant differences in $O_2$ cost. This supports the use of $O_2$ cost for pre to post surgical outcome evaluation in the presence of modest speed differences.

## EP49

### Safety profile of three guided growth implants: body weight correlates with implant failure

Yong-Woon Shin[1], Tyler Uppstrom[1], Samir Trehan[1], Roger Widmann[1], Daniel Green[1]

[1]Hospital for Special Surgery, New York, USA

**LEVEL-III/Hip/Lower extremity**

*Purpose*: The purpose of this study was to evaluate retrospectively the safety profile of three different implants used to correct lower extremity malalignment in paediatric patients.
*Methods*: This retrospective study reviewed a consecutive series of children who underwent implant-mediated guided growth performed by two surgeons between 2004 and 2014. Implants were selected based on surgeon preference. Each patient received post-operative radiographs and patient medical records were analysed. Deformity correction and implant integrity were assessed on post-operative radiographs. Screw pull-out, defined as >5 mm, was also recorded. Rate of correction was determined for all cases that completed guided growth treatment.
*Results*: During the study period, 115 plates were implanted in 52 patients (24 males, 28 females). Average age at implantation was 12.3 (range 4.3–16.5) in boys and 11.1(range 6.4–15.0) in girls. Average length of follow-up was 18.4 months. Of the 115 plates in this series, 63 were peanut plates (Biomet, Warsaw, Indiana), 30 eight-plates (Orthofix, Lewisville, TX) and 22 hinge plates (Pega Medical, Montreal, ON). There was no statistically significant difference in rate of correction between implant types ($p = 0.081$).
Three (2.6 %) screw breakages were observed which all involved cannulated screws in peanut plates. A total of four peanut plates (6.3 %) demonstrated an implant-related complication: three cannulated screw breakages and one screw pull-out. Two of the 30 eight-plates (6.7 %) showed partial screw pull-out and one eight-plate (3.3 %) showed partial screw pull-out with bending of a cannulated screw. There were no reported complications within the hinge plate group. Multivariate analysis demonstrated that increased body weight was significantly associated with implant complications ($p = 0.046$). There were no deep infections, premature growth arrests or plate breakages in this cohort.
*Conclusion*: Implant-mediated guided growth is a safe technique for paediatric lower extremity deformity correction with a low rate of complications. Obese patients were at a significantly increased rate of implant complications in our cohort. Screw breakages were only observed in cases with cannulated screws and the less flexible peanut plates.
*Significance*: This retrospective review evaluating three guided growth implants demonstrated an increased implant complication rate in obese patients. In addition, we recommend using solid, non-cannulated screws in obese children who are at higher risk of hardware failure.

## EP50

### Low-risk of physeal damage from a medial patellofemoral ligament (MPFL) reconstruction technique that uses an epiphyseal socket in children

Jonathan Haskel[1], Tyler Uppstrom[1], Elizabeth Gausden[1], Daniel Green[1]

[1]Hospital for Special Surgery, New York, USA

**LEVEL-III/Hip/Lower extremity**

*Purpose*: The purpose of this study was to assess the short-term distal femoral growth plate safety associated with femoral sockets for hamstring autograft fixation in a paediatric MPFL reconstruction.
*Methods*: We retrospectively reviewed a consecutive series of 31 patients (22 females, 9 males) that underwent MPFL reconstruction by one surgeon at a tertiary care academic medical center between 2008 and 2014. Study inclusion criteria consisted of patients who were skeletally immature at the time of surgery, who had >1 year radiographic follow-up and who had a femoral socket introduced during their procedure. Femoral socket location was verified by intraoperative fluoroscopy. All patients received a post-operative X-ray and a clinical follow-up. For the 12 patients who had a post-operative MRI, growth plate safety was assessed by examining the location of the femoral socket relative to the physis to rule out growth plate injury. Development of lower limb angular deformities or limb length discrepancies was evaluated by examining post-operative standing hip-to-ankle antero-posterior radiographs, patient records, and clinical assessments.
*Results*: The average age at surgery of 13.0 years. The average length of radiographic follow-up was 1.5 years and the average length of clinical follow-up was 1.78 years. At most recent clinical follow-up, 90 % (28/31) of patients reported no subsequent patellar dislocations in the treated knee. No patients showed evidence of an angular deformity or limb length discrepancy. Of the 12 patients with postoperative MRI, all showed femoral sockets positioned distal to the physis without growth plate disturbance.
*Conclusions*: The use of an epiphyseal femoral socket for graft fixation presents minimal risk of physeal violation and ensures patellar stability in the majority of paediatric patients. We have demonstrated that using fluoroscopic assistance to place the femoral socket distal to the distal femoral physis is a reliable and safe method for avoiding physeal injury in children with patellar instability.
*Significance*: Anatomic MPFL reconstruction with hamstring autograft can be performed safely in children with open growth plates.

## EP51

### All-inside, all-epiphyseal ACL reconstruction in skeletally immature athletes: return to play, incidence of second surgery and 2-year clinical outcomes

Daniel Green[1], Jessica Graziano[1], Polly DeMille[1], Theresa Chiaia[1], Moira McCarthy[1], Danyal Nawabi[1], Frank Cordasco[1]

[1]Hospital for Special Surgery, New York, USA

**LEVEL-II/Hip/Lower extremity**

*Purpose*: The purpose of this study was to evaluate 2 years clinical outcomes of an all-inside, all-epiphyseal ACL reconstruction in skeletally immature athletes with 3–6 years of remaining growth, focusing on return to play and incidence of second surgery.
*Methods*: 23 skeletally immature athletes (6 females, 17 males) were prospectively evaluated after an all-inside, all-epiphyseal ACL reconstruction utilizing hamstring autograft. The mean chronological age was 11.8 years (range 10–13) and the mean bone age (Greulich and Pyle method) was 12.1 years. Lacrosse (38 %) and soccer (31 %) were the sports most commonly associated with ACL injury in our cohort. All athletes were evaluated with a physical examination, KT-1000 arthrometry, isokinetic testing, and validated outcome scores (IKDC, Lysholm and Marx activity scales). Standing radiographs and SPGR MRI analysis was performed at 6, 12 and 24 months post-operatively. A 'Return to Play' analysis was performed postoperatively to assess symmetry, alignment, control and the ability to decelerate and return to play safely.
*Results*: At a minimum follow-up of 2 years (range 2–4.5), the mean IKDC score was 94.5 ± 5.4, the mean Lysholm score was 98.1 ± 4.1 and the mean Marx activity score was 12.8 ± 3.1. Lachman and pivot shift testing was negative in all patients. The mean side-to-side difference in the KT-1000 arthrometry was 0.9 ± 0.6 mm, with a maximum difference of 2 mm. Isokinetic testing showed a mean deficit of 4.3 % in extension torque and 9.1 % in flexion torque at a repetition speed of 180°/s. No angular deformities, significant leg length discrepancies or physeal disturbances were observed on post-operative radiographs or MRI. 2 athletes (8.6 %) required a second surgery: 1 (4.3 %) underwent revision ACLR for traumatic graft disruption at 10 months, and 1 (4.3 %) required meniscectomy for an incompletely healed meniscal repair at 13 months. There were no contralateral injuries in this cohort of athletes. The mean time for return to unrestricted competitive activity after successful completion of the 'Return to Play' analysis was 12.5 ± 1.25 months from the time of surgery.
*Conclusions*: An all-inside, all-epiphyseal ACL reconstruction technique demonstrates excellent clinical outcomes in skeletally immature athletes without growth disturbance.
*Significance*: All-inside, all-epiphyseal ACL reconstruction using hamstring autograft can be performed safely with good short-term clinical results.

## EP52

### Long-term follow-up of pediatric ACL reconstruction in New York State: high rates of subsequent ACL reconstruction

Emily Dodwell[1], Moira McCarthy[1], Ting-Jung Pan[1], Daniel Green[1], Stephen Lyman[1]

[1]Hospital for Special Surgery, New York, USA

**LEVEL-II/Hip/Lower extremity**

*Purpose*: The primary aim of this study was to determine the rate of subsequent ACL reconstruction and additional non-ACL knee surgery in children that had previously undergone ACL reconstruction in New York State. The secondary aim was to assess factors associated with additional ACL and non-ACL knee surgery.
*Methods*: Paediatric patients (age <21 years) who underwent ACL reconstruction between 1997 and 2010 in New York State were identified using the Statewide Planning and Research Cooperative System (SPARCS) database. Patients were tracked for subsequent ACL reconstruction and additional non-ACL knee surgery. Each case had a minimum of 1 year follow up. A Cox proportional hazards model was used to assess time to subsequent surgery, adjusting for age, sex, race, comorbidity index, insurance type, surgeon and hospital ACL volume, and poverty prevalence.
*Results*: 23,912 primary pediatric ACL reconstructions were identified. 1,955 patients (8.2 %) underwent subsequent ACL reconstruction. 7.4 % had one additional ACL reconstruction and 0.7 % had two or more additional ACL reconstructions. 3,341 patients (14 %) had subsequent non-ACL knee surgery with 11.1 % having one subsequent surgery and 2.9 % having two or more additional knee surgeries. Risk factors for revision ACL surgery were younger age at time of primary ACL surgery ($p < 0.001$), male gender ($p < 0.001$), white race ($p = 0.003$), private insurance ($p < 0.001$), higher hospital ACL volume (>20 per year; $p = 0.002$), and higher surgeon ACL volume (>10 per year; $p = 0.003$). Risk factors for return to the OR for other non-ACL knee surgeries were younger age at the time of primary ACL reconstruction ($p < 0.001$), white race ($p < 0.001$), private insurance ($p = 0.016$), and higher hospital ACL volume (>20 per year; $p = 0.007$).
*Conclusion*: This study aims to provide information on the rate of return to the operating room and rate of secondary ACL reconstruction in an adolescent population over the past 20 years in New York State. Males and younger patients had a higher rate of subsequent ACL reconstruction. Higher rates of ACL reconstruction in younger children may be due to greater at-risk activities in younger children, longer follow up, or inherently less reliable reconstructions given the non-anatomic reconstructions that are frequently required in the skeletally immature. The association between socioeconomic factors and increased subsequent knee surgeries likely represents a disparity in access to care for low socioeconomic status patients.
*Significance*: This study is the first to evaluate, on a population level, the rate of subsequent ACL surgery and additional non-ACL knee surgery following primary pediatric ACL reconstruction.

## EP53

### The epidemiology and demographics of pediatric supracondylar humerus fractures in New York State

Alexia Hernandez-Soria[1], Emily Dodwell[1], Ting-Jung Pan[1], Stephen Lyman[1], Aaron Daluiski[1]

[1]Hospital for Special Surgery, New York, USA

**LEVEL-II/Trauma/Upper extremity**

*Purpose*: Supracondylar humerus fractures (SCHF) are the most common elbow fractures in children, although the true incidence is not known. The purpose of this study was to define the incidence of complications and reoperation following the surgical treatment of SCHF in children.We hypothesized that surgeons who treat a higher volume of pediatric SCHF may have lower reoperation and complication rates.
*Methods*: The SPARCS database (a census of New York State hospital admissions and ambulatory surgical procedures) was used to identify all SCHF in patients <16 years old treated in New York State between 1997 and 2009. The first admission for each patient with a SCHF was considered the index procedure. The license number for the primary attending surgeon is also recorded allowing calculation of surgeon volume. Patient complication and reoperation records were identified. These included Volkmann's contracture, compartment syndrome, peripheral nerve injury, claw hand, wrist drop, cellulitis, osteomyelitis, median nerve palsy, radial nerve palsy and ulnar nerve palsy. Multivariate logistic regression analysis was used to evaluate the association of surgeon volume with the likelihood of complications and reoperation.
*Results*: 25,872 patients were admitted for SCHF between 1997 and 2010. The mean age was 6.6 ± 4.1 years. 57.4 % were male and 43 % were female. Within 1 year of initial diagnosis 71 (0.3 %) were admitted for a Volkmann's ischemic contracture, 43 (0.2 %) for cellulitis or osteomyelitis, 10 (0.1 %) for a nerve palsy and 923 (3.6 %) underwent a reoperation. Of the 923 patients who underwent reoperation within 1 year, 773 (83.8 %) were treated by a low volume surgeons (<6 cases/year) and 150 (16.3 %) by a high volume surgeons (>6 cases/year). Patients treated by high volume surgeons had a lower risk of reoperation than patients treated by low volume surgeons [OR 0.61; 95 %CI (0.51, 0.73); $p < 0.001$].
*Conclusion*: Patients treated by high volume surgeons have a lower risk of reoperation compared to those treated by a lower volume surgeon. Despite limitations inherent in the database model, the overall rate of complications and reoperations is comparable to the current literature.
*Significance*: Further study is required to identify the specific factors that contribute to the higher reoperation rates such as patient comorbidities, fracture complexity and surgeon characteristics (e.g. fellowship training, years in practice, practice setting, etc.).

## EP54

### Percutaneous transphyseal screw epiphysiodesis: efficacy and complications

Emily Dodwell[1], Elise Bixby[1], Kristina Astone[1], Roger Widmann[1]

[1]Hospital for Special Surgery, New York, USA

**LEVEL-III/Hip/Lower extremity**

*Purpose*: Previous reports have identified a number of complications associated with percutaneous transphyseal screw epiphysiodesis (PTSE), including implant failure, implant prominence, angular deformities, and delayed growth inhibition. The aim of this study was to assess the efficacy and complications associated with screw epiphysiodesis at a single high volume orthopedic center.
*Methods*: Chart review identified all patients who underwent distal femoral and/or proximal tibial PTSE between 2007 and 2013 inclusive. Complications including pain, knee effusion, angular deformity, unscheduled implant removal, infection and failed epiphysiodesis were recorded. For patients with complete pre- and post-operative calibrated imaging, total limb length and length of each tibial and femoral segment were recorded pre-operatively and at skeletal maturity. The Multiplier Method was used to calculate expected length at maturity of each bone segment, and expected physeal inhibition for each bone segment. Efficacy (actual growth inhibition divided by expected growth inhibition) was calculated. Mean leg length discrepancy (LLD) was evaluated at maturity.
*Results*: 62 patients with 96 treated physes (47 distal femurs and 49 proximal tibias) were included. 35 % of patients had complications including pain (N = 22, 19 %), failed epiphysiodesis requiring revision (N = 2, 3 %), knee effusions (N = 4, 6 %) and angular deformity (N = 4, 6 %), defined as a change in mLDFA or MPTA $^{3}$5°. No deformities were severe enough to require revision. 27 % of patients underwent screw removal.
Average LLD at maturity was 1.8 cm (SD = 0.7 cm). 81 % of patients underwent epiphysiodesis later than the predicted ideal surgical timing. Delayed epiphysiodesis was primarily due to late presentation of the patient, or surgeon preference to err on the side of undercorrection. Surgeon experience with the procedure was not associated with improved length discrepancy at maturity.Overall, efficacy was 110%; 107 % for distal femur epiphysiodesis and 113 % for proximal tibia epiphysiodesis, suggesting that PTSE on average provided slightly more growth inhibition than calculated.
*Conclusion*: PTSE is an effective method of permanent epiphysiodesis. Although 35 % of patients experienced some form of complication, the majority were mild and transient, not requiring additional unplanned surgery. Contradicting previous reports, significant angular deformities were not identified in this series.
*Significance*: Contrary to previous reports, PTSE in this series did not result in significant angular deformity, insufficient growth inhibition or other serious complications. Further comparative studies, such as a randomized trial, could further elucidate the relative outcomes for this and other epiphysiodesis techniques.

## EP55

### Defining CT scan parameters of talus in relapsed clubfoot treated previously by soft tissue release and its correlation with ICFSG scoring: search for a novel outcome predictor in older children!

Nirmal Raj Gopinathan[1], Venkatramana Kuruba[1], Pebam Sudesh[1], Akshay Kumar Saxena[2]

[1]Department of Orthopaedics, PGIMER, Chandigarh, India;
[2]Department of Radiology, PGIMER, Chandigarh, India

**LEVEL-II/Club-foot**

*Introduction*: The importance in search for a predictive prognostic factor in children affected with clubfoot is important as the results of management either conservative or surgical is not always satisfactory. Though the diagnosis of congenital talipes equino varus is obvious by clinical examination itself, radiographs and other imaging modalities like ultrasonogram are tested repeatedly to ascertain the severity and to predict the prognosis of treatment. There are many classification schemes, but none offers adequate prognostic value. No parameter has been described to accurately define the severity of the deformity. In an attempt to define parameter that correlates with the severity of the deformity as well predict the prognosis of the outcome of any method of treatment; an attempt has been done to measure the anatomical angles on CT scan of the affected feet defining the morphology of deformed talus as well as the interrelation between the tarsal bones.
*Materials and Methods*: We studied on 20 children with 29 clubfeet, with a mean age group of 7.9 years. All cases had received manipulative casting and soft tissue release before 2 years age and had relapse by the time of presentation. Severity was clinically assessed by ICFSG scoring and 3 of them had GOOD, 17 had FAIR and 9 had POOR outcome scoring. This was correlated with angles measured on CT 3D reconstructions of the feet, Torsion of talar head, talar neck-body angle (medial inclination and plantar inclination) and talocalcaneal angles in AP and lateral 3D images.
*Observations and Results*: Significant correlation was found with Talar torsion with a p value of 0.007, talocalcaneal angle AP with *P* value OF 0.008, talocalcaneal angle lateral with *P* value of 0.03, medial inclination of head of talus with *P* value of 0.001. Plantar inclination of head of talus is insignificantly related with p value at $>0.05$
*Conclusion*: An attempt was made to measure the three dimensional characteristics of talus using a 3D CT Scan and to correlate it with severity of the relapsed deformity. The talar torsion angle correlated significantly with the deformity and we suggest that these parameters can be better predictors of outcome and needs to be studied further in detail.

## EP56

## Comparison of healing characteristics of humeral and femoral lengthenings

Jan Poul[1], Karel Urbasek[1], Karel Rocak[2]

[1]University Hospital Brno, Brno, Czech Republic
[2]Hospital Prerov, AGEL, Prerov, Czech Republic

**LEVEL-III/Limb reconstructions**

*Aim*: To compare healing characteristics among groups of femoral and humeral lengthenings.
*Materials and Methods*: 28 femoral lengthenings in 26 patients, 16 girls, 10 boys. Most commonly Mefisto external fixator was used (Prospon 1x, Orthofix 3x, Mefisto 24x). In 15 cases congenital shortening of femur, in 5 secondary shortening of femur due to impairment of distal femoral physis, in two cases shortening ofter osteomyelitis, in one case shortening after avascular necrosis of femoral head, in one case shortening after pathological fracture of femur, in one case disturbance of distal femoral physis due to enchondromatosis and in one case overgrowth after fracture. Average age at the beginning of lengthening was 11 (4–16 years). 20 humeral lengthening in 18 patients. Orthofix external fixator most commonly used (Orthofix 11x, Prospon 2x, Mefisto 7x). In nine cases shortening after osteomyelitis of proximal humerus, in five congenital shortening, in four secondary shortening after tumor involvement of proximal humeral physis. Average age at the beginning of lengthening 13 (8–19 years).
*Results*: Femoral lengthening: Average lengthening of femur in all pts was 40.2 mm (SD $\pm$11.1), index of osteotomy (OI) 41 % (SD $\pm$9.8), lengthening percentage (LP) 10.9 % (SD $\pm$3.8), lengthening index (LI) 14.5 days/cm (SD $\pm$3.5), healing index (HI) 52.6 days/cm (SD $\pm$0.1), consolidation index (CI) 93.3 days/cm (SD $\pm$40.0). Number of complications: minor and moderate 11 (39.2 %), serious and critical 8 (28.6 %). 14 pts (53.8 %) completely without any complication Two complications developed in 5 pts (17.9 %). Lengthening percentage versus number of complication showed in statistical testing significant difference ($p = 0.019$). On the other side there was no stastistically significant difference between healing index and age at time of operation ($p = 0.836$), respectively the gender ($p = 0.546$) (Mann–Whitney test). Relation between IO versus HI was statistically not significant ($p = 0.492$). Correlation of lengthening index and healing index was statistically significant ($p < 0.001$). Humerus lengthening: Average lengthening of humerus was 70.5 mm, LI 13.2 days/cm, HI 28.6 days/cm, CI 44.5 days/cm. Number of complications: minor and moderate 4, serious and critical 5. Eleven lengthenings were without any complications.
*Discussion*: Comparison of both studies showed that healing index as well as consolidation index are for humerus half against femur.
*Conclusion*: This findings can help to predict the length of treatment specifically for humerus and femur.

## EP57

## Non-operative treatment of pediatric forearm fractures yields excellent functional results

João Diogo Castro[1], Cristina Alves[1], Inês Balacó[1], Pedro Sá Cardoso[1], Tah Pu Ling[1], Gabriel Matos[1]

[1]Hospital Pediátrico-CHUC, EPE, Coimbra, Portugal

**LEVEL-II/Trauma/Upper extremity**

*Purpose*: The objective of this study is to evaluate the functional results of the non-operative treatment of forearm fractures, in children aged between 5 and 17 years old, using the Disability of Arm, Shoulder and Hand (QuickDASH) Score.
*Methods*: Patients diagnosed with a diaphyseal fracture of the forearm bones and treated in our institution during the year of 2012, by means of closed reduction and cast immobilization, were recruited to this study. Patients who had a surgical intervention were excluded. Data was collected regarding the demographics of the patients, the nature of the accident, type of fracture and fracture healing. Patients were interviewed on the phone and the functional evaluation was done using the Quick-DASh Score.
*Results*: During the period of the study, we identified 46 patients with a forearm fracture, needing reduction. Surgical tretamnet was undertaken in 16 patients, who were therefore excluded from this study. We evaluated 30 patients treated by closed reduction and cast immobilization. 18 patients were boys and 12 were girls., with an average age of 10 $\pm$ 3.19 years. A simple fall was the most common causal accident (63 %). In 70 % of cases, there was a complete fracture and 60 % of these were located in the distal 1/3 of the diaphysis. Closed reduction under general anesthesia was performed in 83 % of fractures. In 93 % of cases, an anatomical reduction was obtained. All fractures united and healed within 4 ± 0.81 weeks. Patients had 2 ± 1.24 follow-up appointments and 4 ± 1.34 radiographs from the moment of diagnosis to the definitive discharge from the Outpatient Clinic. We applied the Quick.ASH score through a telephone interview and evaluated the patients at 30 ± 2.82 months of follow-up. Excellent results (QuickDASH < 5) were obtained in 86 % of patients. There were no patients with a bad result. The anatomical location of the fracture did not influence the functional results. We found 1 patient with a residual deformity of the forearm, who was not satisfied.
*Conclusion*: Non-operative treatment of diaphyseal forearm fractures in the paediatric age patients yields excellent functional results.
*Significance*: Understanding the properties of the paediatric skeleton is essential to make proper decisions in the management of paediatric forearm fractures. Most of these lesions can be treated non-operatively.

## EP58

## Split paediatric forearm POP casts do not need reinforcing

Nimesh Patel[1], Lance Wilson[2], Guy Wansbrough[3]

[1]Torbay Hospital, Torquay, UK
[2]University of Technology, Queensland, Australia
[3]Torbay Hosptital, Torquay, UK

**LEVEL-II/Trauma/Upper extremity**

*Purpose*: Displaced paediatric forearm fractures are most often treated by manipulation under anaesthetic, followed by the application of a circumferential Plaster of Paris (POP) cast. Some surgeons choose to split part, or all of the cast in order to facilitate immediate "spreading" with minimal distress to the patient, should the distal limb become compromised. Usually however, this does not occur, and the cast is completed at a later visit to the plaster room. Time, money and inconvenience could be saved if this modification was not necessary, and the final plaster would be lighter. This study set out to establish whether the mechanical properties of a split POP are sufficient to stabilise a forearm fracture, and protect the patient from further injury.
*Methods*: The repeatability of all tests was established on control samples before undertaking the trial. 42 standardised 8 layer POP cylinders of appropriate dimensions were fabricated, of which 21 were split longitudinally. The splints were subjected to non-destructive tests for bending, kinking and torsion, and the load recorded at clinically relevant end-points. These simulated the deformity at which the splint no longer provided adequate stability and alignment, or at which the wearer was no longer protected. The splints were then loaded to destruction to establish the mode of ultimate failure.
*Results*: The mean loads at the clinical end points were 1115N in Bending, 544N in Kinking and 12.3Nm in Torsion (equalling 67.3, 70.4 and 47.4 % of the equivalent values for a circumferential POP). Loads were in excess of body weight for most paediatric patients. After ultimate failure, the proportion of casts that became unstable was similar (44 % of full casts and 50 % of split casts).
*Conclusion*: Split POP casts which have not been spread, provide adequate stabilisation and protection of paediatric forearm fractures, and do not routinely require completion.

## EP59

## The management of thoracolumbar deformity in the mucopolysaccharidoses

Deborah Eastwood[1]

[1]Great Ormond St Hospital, London, UK

**LEVEL-IV/Spine**

*Background*: The mucopolysacchiradoses represent a group of storage disorders caused by enzyme deficiencies. They are characterised by a range of skeletal manifestations, including abnormalities around the thoracolumbar junction. Small series in the literature have reported on these findings. We present one of the largest series on spinal deformity in mucopolysaccharidosis (MPS) patients and the requirement for surgical intervention.
*Materials and Methods*: 194 paediatric patients with mucopolysacchiradoses were reviewed by the metabolic team in a tertiary referral centre. 107 of these patients underwent spinal imaging in this unit during a 10 years period from 2003 to 2013. Data collected included the number of patients with each subtype of MPS, the number with thoracolumbar spinal deformity, the presence of thoracolumbar vertebral morphological abnormality, and the number of patients progressing to surgery. The need for revision surgery was also reviewed.
*Results*: 107 of the 194 patients underwent spinal imaging. This included 45 patients with MPS I, 23 with MPS II, 20 with MPS III, and 19 with MPS IV. Deformity was present in 75 patients (70 %) and was most common in MPS I (80 %) and IV (89 %). Morphological abnormality was seen in 54 patients (50 %) with involvement in 89 % of MPS IV patients but only 5 % of MPS III patients. 29 patients (27 %) required surgery for progressive deformity not controlled by non-operative measures. 22 patients underwent anterior fusion, 4 had posterior fusion, and 3 had anterior and posterior procedures. 5 patients (23 %) required further procedures for progressive adjacent deformity.
*Discussion*: MPS is an uncommon aetiology for thoracolumbar deformity. However, a large number of these patients have abnormal vertebral morphology and it is important to understand the optimum methods of managing progressive deformity. Further work on the role of enzyme therapy and its effect on spinal deformity in MPS will help guide the non-operative measures available to clinicians.

## EP60

## Selective ultrasound screening is inadequate to identify patients who present with symptomatic adult acetabular dysplasia

Ernest Sink[1], Benjamin Ricciardi[1], Katrina Dela Torre[1], Charles Price[1]

[1]Hospital for Special Surgery, New York, USA

**LEVEL-IV/DDH**

*Purpose*: Many patients presenting with symptomatic acetabular dysplasia at skeletal maturity are not diagnosed with DDH during infancy. Selective ultrasound screening may identify patients with neonatal hip instability but may be ineffective for prevention of dysplasia presenting in adulthood. The purpose of this study is to identify the prevalence of risk factors for DDH that would have warranted selective ultrasound screening in patients with symptomatic acetabular dysplasia after skeletal maturity.
*Methods*: Patients with symptomatic acetabular dysplasia presenting between June 2011 and December 2013 were enrolled through our prospective single-center hip preservation registry. Inclusion criteria included a lateral center edge angle (LCEA) on the AP radiograph <20° and/or an anterior center edge angle (ACEA) <20° on the false profile radiograph. Exclusion criteria included neuromuscular dysplasia, teratologic hip dislocation, or inability to confirm birth history. We retrospectively identified a consecutive series of 68 skeletally mature patients recommended to have corrective osteotomy for symptomatic hip dysplasia. Prospective data was collected at the time of initial consultation from all patients regarding their birth history and risk factors for DDH including: family history of hip pain in first or second-degree relatives, hip surgery, or DDH; breech, method of delivery, previous treatment or surgery on their hips and birth order. LCEA, ACEA, Tonnis grade, and Tonnis angle were recorded from plain radiographs.
*Results*: 67 females and 1 male were identified. No patients were previously diagnosed with DDH or received treatment for their hips. Eight patients (11.8 %) were confirmed breech. A family history of DDH was present in two additional patients (2.9 %). Therefore, current United States guidelines would recommend selective ultrasound screening in 10/68 patients (14.7 %) of this cohort. Characteristics that were identified but are not current indications for selective ultrasound screening included 36 patients (52.9 %) that were first born, 25 patients (36.8 %) with a family history of hip osteoarthritis in a first or second degree relative (but not confirmed by family as DDH) and 5 patients (7.4 %) who were born premature. Despite the presence of symptomatic hip dysplasia at skeletal maturity, no patients in this series were diagnosed with DDH as children or received any treatment for their hips.
*Conclusions*: The majority (85.3 %) of patients with symptomatic acetabular dysplasia at skeletal maturity would not have met current recommendations for selective ultrasound screening in the United States had they been born today.

## EP61

## Ponseti recasting for congenital clubfoot residual deformities after walking-age

Giuseppe Vena[1], Pasquale Farsetti[1], Ernesto Ippolito[1]

[1]University of Rome Tor Vergata, Rome, Italy

**LEVEL-III/Foot and ankle**

*Background*: Although the Ponseti method has shown excellent results in patients affected by clubfoot, residual deformities after walking age still represent a challenge in paediatric orthopaedics and there is no consensus on the best modality of treatment. The purpose of our paper was to review the results of treatment by re-casting according to Ponseti in 44 patients (68 clubfeet) with recurrent or persistant deformities after walking age.
*Methods*: Our series included 44 patients (68 feet) with idiopathic congenital clubfoot previously treated in other institutions by either conservative or surgical management. The mean age was 2.8 years (range from 1 to 7 years) and the common feature of all our cases was foot stiffness. Manipulations and recasting were performed under sedation either in restless children or in very rigid deformities. The number of plaster-casts ranged from 2 to 4 and each cast was maintained for 2–4 weeks. During the index procedure, 28 clubfeet required a percutaneous Achilles tenotomy, and 6 clubfeet a percutaneous plantar fasciotomy. In 20 clubfeet, tibialis anterior tendon transfer was performed after recasting.
*Results*: Objective improvement of the residual deformities was achieved in all cases. Equinus, heel varus and supination were the deformities best corrected. Cavus and adduction were more resistant than the other deformities to full correction, even when plantar fasciotomy was performed. The most striking result of our treatment was the recovery of foot flexibility even in very rigid feet.
*Conclusions*: Our results show that recurrent and persistant clubfoot deformities may be successfully treated by recasting followed by bracing after walking age up to 2.5 years of age. Whereas in older children, tibialis anterior tendon transfer after recasting was necessary to maintain the correction.

## EP62

## Manual angle measurement reproducibility and reliability using EOS ultra-low dose two dimensional stereography in adolescent idiopathic scoliosis

Vincent Salabi[1]

[1]Robert Debré Hospital, Paris, Paris, France

**LEVEL-II/Spine**

*Introduction*: EOS imaging system has been described to deliver 8–10 times less radiation exposure than conventional full spine X-ray. It has been methodologically validated for usual angles and distances in computed 2D and 3D analysis of the spine. In daily practice, pediatric surgeons often do not have the 3D reconstructions available for each patient during the consultation, and follow-up of the spinal deformity is performed with manual measurements. Reliability and reproductibility of manual mesurement on 2D EOS ultra-low dose biplanar stereography in an Adolescent Idiopathic Scoliosis (AIS) population have to be evaluatued in order to validate its use in daily practice.
*Patients and Methods*: This monocentric series included 50 AIS patients with more than 10° Cobb angle. Patients underwent ultra low-dose standing biplanar stereoradiographs. Three operators (junior pediatric surgeon, senior pediatric surgeon, senior pediatric radiologist) performed sixteen classical angle mesurements for each patient, eight on the antero-posterior (AP) view and eight on the lateral view. The measurements were performed once manually on the films and once using a whidespread methodologically validated imaging analysis software. Intraoperator repeatability and interoperator reproducibility were calculated for both measurement methods, and each result was compared to the 3D computed reconstruction considered as the Gold Standard.
*Results*: On the AP view, the reproducibility was 4° for frontal parameters (main Cobb angle, end vertebra, proximal thoracic Cobb angle, lumbar countercurve Cobb angle); on lateral view the reproducibility was 5° for kyphosis and lordosis, 3° for pelvic parameters wether measured on film or on computer. No inferiority of the manual measurement on film or on computer versus the Gold Standard method were found.
*Discussion*: Ultra-low dose EOS delivers up to 48 times less radiation for a full-spine acquisition. One of the most interesting applications of EOS biplanar stereography concerns adolescent idiopathic scoliosis, where reduction of ionizating radiation exposure is of major interest given the fact that these particular patients will have to undergo full-spine imaging repeatedly during several years. Manual measurements of basic frontal and sagittal spinal angles on 2D ultra-low dose EOS are reproducible and reliable in daily practice for AIS diagnosis and follow-up.

## EP63

## Hip contracture in patients with cerebral palsy: external validation of the predicted surgical outcomes using the random forest algorithm

Anne-Laure Simon[1], Krista Coleman-Wood[2], Brice Ilharreborde[1], Kenton R. Kaufman[3]

[1]Robert Debré Hospital, Paris, France
[2]Mayo Clinic, Rochester, USA
[3]Mayo Clinic, Rochester, France

**LEVEL-IV/Cerebral Palsy**

*Aim*: Clear criteria to define patients requiring psoas lengthening procedure are lacking. Since physical examination is not reliable enough, combined criteria from 3D gait analysis might be used. A recent publication by Schwartz et al. introduced the Random Forest Algorithm (RFA) as a preoperative predictive tool for hip function outcomes after single-event multilevel surgery (SEML) with or without psoas lengthening. The aim of the study was to evaluate the validity of their surgical prediction algorithm.
*Methods*: All records of ambulatory children with cerebral palsy were retrospectively reviewed. Selection criteria included (1) hip contracture on physical examination (2) full 3D gait analysis and (3) pre- and postoperative assessments. Collected data included the hip extension angle on physical examination and 3D gait kinematics, kinetics and time-distance parameters, which were used for calculation of the Hip Function Index (HFI), Pelvis–Hip Deviation Index (PHiDI), and the RFA. The HFI and the PHiDI quantitatively assess the dynamic hip and/or pelvis function. Patients with a postoperative PHiDI score <90 or with an improvement of <5 were considered poor results. The RFA assess the need for a psoas lengthening procedure. If the RFA score is more than 58 %, patients were considered to meet the criteria for the procedure. A two-tailed Student t-test or a 2-sample Wilcoxon test was performed for pre- and postoperative means comparisons, depending on the normalcy of the variable distribution.
*Results*: A total of 27 patients (mean age at surgery 7.2 ± 0.6 years) of which 25 underwent a postoperative gait analysis at a mean follow-up of 7.0 ± 0.6 months, were reviewed. None of the patients had a psoas lengthening performed. Preoperative data showed that the patients had poor hip function (mean PHiDI 71.3 ± 2.3, mean HFI 4.9 ± 0.4) and 71 % of the cases met the criteria for a psoas lengthening (mean RFA 69.2 ± 4.0). Postoperative comparisons revealed that there were not significant changes in hip function (PHiDI 68.3 ± 1.6, $p = 0.3$; HFI 4.7 ± 0.3, $p = 0.72$; RFA 68.5 ± 4.3, $p = 0.85$). Only the hip extension angle was significantly improved after a SEML surgery without psoas lengthening (−7.5° ± 1.4° vs. −2.5° ± 1.3°, $p = 0.04$).
*Conclusion*: The study confirms the specificity of the RFA for hip dysfunction in children with cerebral palsy. A SEML surgery without psoas lengthening does not improve dynamic hip function.
*Significance*: External validation of the RFA for prediction of hip and pelvis function outcomes in children with cerebral palsy.

## EP64

## The effectiveness of pre-operative halo-gravity traction (HGT) in early onset scoliosis (EOS)

Ana Maria Ey Batlle[1], Norbert Ventura Gómez[1], Inmaculada Vilalta Vidal[1], Augusto Covaro[1], Melisa Stitzman Wengrowicz[1]

[1]Hospital Sant Joan de Déu, Esplugues (Barcelona), Spain

**LEVEL-III/Spine**

*Summary*: Treatement of complex spinal deformity in EOS remains a challenge. A rigid spinal deformity, poor pulmonary function, malnourished patients, poor bone stock complicated and surgical treatments.
*Introduction*: HGT is able to restore coronal and sagital balance, improve pulmonary function and reduce the risk of neurological injury.
*Methods*: A retrospective review of 21 patients with severe rigid scoliosis or kyphoscoliosis was performed to assess the safety and efficacy of HGT. The use of HGT in 15 patients was preoperative and in 6 perioperative. The analysis focused on the impact of HGT on curve flexibility, thoracic improvement, complications and surgical outcomes in a single spine center between 2005 and 2011 (mean follow-up: 35 months). Space Available For the Lung (SAFL) and T1–S1 distance were used to measure thoracic improvement. HGT traction protocol included 30–40 % of patients' weight (depending on patients condition) during 8 weeks.
*Results*: 21 patients, 8 males and 13 females. Mean age 9.33 (range 3–17 years). Etiologies were 1 idiopathic, 1 congenital, 10 neuromuscular and 9 others (6 arthrogryposis, 2 osteogenesis imperfecta and 1 syndromic).
Mean pre-HGT values were: coronal Cobb: 99.3° (range 62–146), sagittal Cobb: 82° (34–125), SAFL index: 79 (48–127), T1–S1 distance: 235 mm (143–345).

With HGT mean values were: coronal Cobb: 70° (27.7 % of improvement), sagittal Cobb: 62.8° (21.5 % of improvement), SAFL index: 83.2 (5.3 % of improvement) and T1–S1 distance: 269.5 mm (14.2 % of improvement).

At the end of follow-up mean values were: coronal Cobb: 57.5° (41 % of improvement), sagittal Cobb: 53.7° (24.6 % of improvement), SAFL: 86.6 (9.6 % of improvement) and T1–S1 distance: 298.1 mm (26.9 % of improvement).

No neurological complications had been observed.

These results are consistent with others published in the literature. Despite 1 pin infection that needed removal and antibiotic treatment there were no serious complications.
*Conclusion*: We found that HGT is not only safe and useful as a preoperative treatment in patients with severe rigid scoliosis, but also as a perioperative adjuvant in complicated kyphoscoliosis. Significant deformity correction averaging 24.6 % can be expected during HGT treatment; this correction is maintained or even improved with subsequent surgical correction.

## EP65

### Could we improve the healing of chondral defects in osteocondhitis diseccans? In vitro and in vivo study

Patricia Sanz-Ramos[1], Julio Duart-Clemente[2], Maria-Victoria Rodriguez-Goñi[3], Gonzalo Mora[4], Mikel Vicente-Pascual[5], Javier Dotor[5], Iñigo Izal[5]

[1]University of Navarra, Pamplona, Spain
[2]Navarra Hospital Complex, Pamplona, Spain
[3]Alava University Hospital, Vitoria-Gasteiz, Spain
[4]Centro de Traumatología Dr Mor, Logroño, Spain
[5]University of Navarre, Pamplona, Spain

**LEVEL-I/Basic science**

*Purpose*: Osteochondritis dissecans is a challenging clinical problem. Autologous chondrocytes implantation (ACI) may be a good choice for the treatment of large defects. Their use is limited because of loss of the cartilage phenotype during expansion. The mechanosensing capacity of chondrocytes suggests evaluating the use of soft substrates for in vitro expansion. The aim of this study was to test the expansion of chondrocytes on collagen hydrogels to improve their capacity for chondrogenesis after a number of passages.
*Methods*: Rat cartilage cells were expanded on collagen hydrogels and on plastic, and the preservation of their chondrogenic capacity was tested both in vitro and in vivo. The expression of relevant markers during expansion on each surface was measured by real-time polymerase chain reaction (PCR). Expanded cells were then implanted in focal lesions in the medial femoral condyle of healthy sheep, and the newly formed tissue was analyzed by histomorphometry.
*Results*: Compared with cells cultured on plastic, cells cultured on hydrogels had better maintenance of the expression of the Sox9, Col2 (type-II collagen), FGFR3, and Alk-5 genes and decreased expression of Alk-1 and BMP-2. Pellets also showed increased expression of the cartilage marker genes aggrecan, Sox9, and Col2, and decreased expression of Col1 and Col10 (type-I and type-X collagen). Enzyme-linked immunosorbent assay (ELISA) also showed a higher ratio of type-II to type-I collagen in pellets formed from cells expanded on hydrogels. When sheep chondrocytes were expanded and implanted in cartilage lesions in the femoral condyle of healthy sheep, hydrogel-expanded cells produced histologically better tissue compared with plastic-expanded cells.
*Conclusions*: The expansion of chondrocytes on collagen hydrogels yielded cells with an improved chondrogenic capacity compared with cells expanded on plastic.
*Significance*: The study results favor the use of hydrogel-expanded cells over the traditional plastic-expanded cells for autologous chondrocyte implantation.

## EP66

### Age of recurrence in idiopathic clubfoot treated with the Ponseti method

Rachel Goldstein[1]

[1]Children's Hospital LA, Los Angeles, USA

**LEVEL-III/Club-foot**

*Purpose*: Previous studies of patients undergoing the Ponseti method for idiopathic clubfoot often demonstrate excellent correction and plantigrade feet after initial treatment. However, recurrence occurs in one-third of cases, and the only known factor to mitigate this is compliance with brace wear. This is difficult to adhere to when parents are told that recurrence can occur up to age 5–7 years. To date, few studies have demonstrated the precise age at which recurrence typically occurs.
*Methods*: All patients with idiopathic clubfoot treated with Ponseti method at our institution with >3 years of follow-up were evaluated. Demographic and treatment data, need for surgical intervention and procedure information were recorded. Dimeglio/Bensahel and Catterall/Piriani scores were recorded at initial presentation, at initiation of FAO, and yearly up to 5 years of age.
*Results*: A total of 110 patients met inclusion criteria. These patients were followed for an average of 5.9 years (range 3–14 years). Thirty-two patients (29.1 %) required surgical intervention, 31 patients (28.2 %) for tibialis anterior tendon transfer (ATTT) and two patients (1.8 %) for posteromedial release (PMR). The average age at which anterior tibialis tendon transfer (ATTT) was carried out was 4.9 years (range 2.3–8.1 years). Posterior medial release (PMR) was performed at an average age of 1.8 years (range 0.3–3.2 years). Patients who eventually required surgery during the follow-up period had significantly higher Dimeglio/Bensahel scores at presentation. Though they maintained higher scores throughout the follow-up period, they did not demonstrate *significantly* higher scores until 3 years of age. These patients also demonstrated higher Catterall/Pirani scores, but again this difference did not become significant until 3 years of age. Scores in both scoring systems in both patient populations continued to increase throughout the follow-up period.
*Conclusion*: Patients with recurrence after initially successful Ponseti treatment generally do not become distinguishable by our current classification systems until 3 years of age. In addition, scores continue to increase throughout the follow-up period, and the average age for an ATTT was almost 5 years old.
*Significance*: These findings reinforce that close follow-up is important at least until 3 years of age, and that ongoing follow-up should probably continue intermittently throughout childhood.

## EP67

### The use of the foot ankle orthosis after Ponseti treatment of idiopathic clubfoot: how long is long enough?

Rachel Goldstein[1], Alice Chu[1], Debra Sala[1], Wallace Lehman[1]

[1]Children's Hospital LA, Los Angeles, USA

**LEVEL-III/Club-foot**

*Purpose*: Foot ankle orthosis (FAO) noncompliance has been identified in numerous studies as a major contributing factor for recurrence in the treatment of idiopathic clubfoot. However, studies have yet to identified how long the FAO needs to be worn to decrease the risk of recurrence. The purpose of this study was determine if age at discontinuation of the FAO was associated with an increased risk of need for surgical intervention.
*Methods*: This was an IRB-approved retrospective review of all patients with idiopathic clubfoot treated with the Ponseti method at our institution. Patients were excluded if they did not follow-up until at least 3 years old. Age at discontinuation of the FAO by either the physician or the parent was noted. Need for surgical intervention, type of surgery, and age at first surgical intervention were also collected.
*Results*: A total of 110 patients met inclusion criteria, and data was available on the use of the FAO for 104 of these patients. Patients were followed for an average of 5.9 years (range 3–14 years). FAO was used until an average age of 2.6 years (range 0.4–5.1 years). There was a significant difference in the age at which the FAO was stopped in those patients who eventually required surgery versus those who did not (2.2 vs. 2.8 years, $p = 0.031$). There was also a significant difference in the need for surgery for patients who used the FAO for at least 2 years (21.6 %) versus those who did not (43 %) ($p = 0.025$).
*Conclusions*: Patients who used the FAO until >2 years of age were 2.77 times less likely to require surgery than those who used it <2 years.
*Significance*: FAO use should be continued until at least 2 years of age in patients undergoing Ponseti treatment for idiopathic clubfoot.

## EP68

### Correction of pelvic obliquity after spinopelvic fixation in cerebral palsy patients: a comparison study

Oussama Abousamra[1], Tristan Nishnianidze[1], Kenneth J Rogers[2], Ilhan A Bayhan[2], Petya Yorgova[2], Suken A Shah[2]

[1]NAIDHC, Wilmington DE, USA
[2]NAIDHC, Wilmington, USA

**LEVEL-III/Cerebral Palsy**

*Introduction*: The optimal choice for spinopelvic fixation in cerebral palsy (CP) scoliosis with pelvic obliquity is controversial. This study aims to compare outcomes among three different instrumentation types: unit rod, iliac screws and sacral alar iliac (SAI) screws in terms of pelvic obliquity correction in children with CP.
*Methods*: Singleinstitution cohort data were collected prospectively and reviewed retrospectively. Patients with minimum 2 years follow up were divided into three groups according to instrumentation type and matched based on preoperative pelvic obliquity and coronal major curve magnitude. Demographic and functional information were reviewed. Radiographic measurements included horizontal pelvic obliquity angle (PO), spinopelvic angle (SPA), coronal and sagittal Cobb angles and T1 pelvic angle (T1PA to evaluate sagittal plane reconstruction). Implant related complications were recorded. Procedures were done by four surgeons in one pediatric institution between 2004 and 2012. All measurements were done by a single independent reviewer who was not involved in the procedures.
*Results*: Seventy-seven patients (42 unit rod, 14 iliac screw and 21 SAI screw) were included. Gender, age and functional status (GMFCS) distribution was similar across all groups [56 % males and 44 % females, mean age 13.5 years (6.3–18.6 years), 84 % GMFCS V]. Mean follow up was 3.6 years. Comparing pre- and postoperative measurements (Results Table), there was a significant decrease ($p < 0.05$) in PO and SPA in all groups. No significant loss of correction occurred during follow up. T1PA improved similarly as PO and SPA in the unit rod and SAI groups, but not in the iliac screw group. Postoperatively, coronal major Cobb angle and kyphosis decreased significantly in all groups with no change during follow up. Lordosis did not change significantly. Nonsymptomatic loosening was noted in 59 % of unit rods, 57 % of iliac screws and 52 % of SAI screws. One prominent iliac screw needed removal. One nonsymptomatic rod fracture, one infected pseudarthrosis and one rod malposition occurred in unit rod group.
*Conclusion*: In patients with CP scoliosis, significant correction of pelvic obliquity, as measured by PO and SPA, was achieved using each of the three reported methods of spinopelvic fixation. This correction was maintained at midterm follow up. All three methods were comparable with no significant differences in correction between them.
*Significance*: This study suggests that, for correction of pelvic obliquity in children with CP scoliosis, iliac and SAI screws were similar to unit rod in comparative effectiveness and implant safety profile.

## EP69

### Slip progression after in situ screw fixation of slipped capital femoral epiphysis

Rachel Goldstein[1], Stephanie Chu[1], Erin Dawicki[2], Herman Luther[2], Lindsay Andras[1], Michael Millis[2]

[1]Children's Hospital LA, Los Angeles, USA
[2]Boston Children's Hospital, Boston, USA

**LEVEL-III/Hip/Lower extremity**

*Purpose*: Slip progression after in situ screw fixation is rarely reported and its true incidence is unknown. Greater than five threads across the physis is the only known factor to minimize slip progression after in situ screw fixation. The purpose of this study was to determine the incidence of continued slip progression after in situ screw fixation and identify potential risk factors for this post-operative complication.
*Methods*: This was a retrospective review of all patients presenting for treatment of a slipped capital femoral epiphysis from January 1, 2003 to December 31, 2012 at two major children's hospitals. Exclusion criteria were previous surgical treatment at an outside institution or lack of follow-up through physeal closure. A slip was considered to have progressed if the Southwick angle increased post-operatively by more than ten degrees prior to physeal closure.
*Results*: A total of 112 patients met the inclusion criteria. 13 of these patients demonstrated an increase in Southwick angle of ten degrees or more after in situ screw fixation (11.6 %). Males were significantly more likely to demonstrate slip progression than females (26 vs. 3 %, $p < 0.001$). Body mass index, weight, age at presentation, duration of symptoms, and co-morbid conditions were not significantly associated with continued slip. Patients with bilateral SCFE at presentation did not have an increased incidence of continued slip ($p = 0.223$). 23 % (12/52) of stable slips demonstrated slip progression versus 14 % (1/7) of unstable slips ($p = 0.598$). Wilson percent displacement ($p = 0.06$) and Southwick angle ($p = 0.899$) at presentation were not associated with continued slip. Less than five screw threads across the physis was associated with continued slip ($p = 0.049$). However, number of screws used was not associated with continued slip ($p = 0.605$).
*Conclusion*: Male patients and patients with <5 threads across the physis were at increased risk of continued slip after in situ pinning.
*Significance*: Male patients have an increased risk of continued slip progression after in situ pinning and may warrant closer follow-up. Greater than five screw threads across the physis decreases the risk of slip progression after in situ pinning.

## EP70

### Factors affecting compliance to hospital visit among clubfoot patients in a developing country: a cross sectional study from a tertiary referral clubfoot clinic

Venkatesan Sampath Kumar[1], Shah Alam Khan[1], Rishi Ram Poudel[1], Ashok Kumar[1], Vivek Tiwari[1]

[1]AIIMS, New Delhi, India

**LEVEL-III/Club-foot**

*Purpose*: Ensuring compliance to treatment protocol, especially regular visit to treating facility, is an important aspect of clubfoot management. Factors affecting compliance to follow-up schedule in developing countries are myriad and they have been never studied.
*Patients and Methods*: A cross sectional study was undertaken among caregivers of clubfoot patients from a tertiary referral clubfoot clinic in a developing country. Hospital records were reviewed to collect demographic data and subjects were classified as either 'regular' or 'irregular' if they missed $\leq$3 and >3 scheduled hospital visits respectively. Various factors that could affect compliance such as family structure, number of children, literacy of care giver, occupation of breadwinner and time taken to travel to hospital were studied. Caregivers were probed regarding the reason for their irregularity.
*Results*: A total of 238 patients were included of which 138 formed the 'regular' group and the rest 100 formed the 'irregular' group. Patients in the regular group were significantly younger (mean age 43.8 months) compared to irregular group (59.8 months) ($p = 0.001$). The mean follow-up period in regular group was 28.1 months and irregular group was 33.8 months and the difference was significant ($p = 0.021$). No statistically significant difference was noted in sex distribution, laterality, syndromic association, literacy of parent and occupation of breadwinner. However, among non-singular children (with at least one more sibling in family), females are more likely to be irregular as compared to males ($p = 0.038$). Patients in irregular group had to travel for longer duration ($p = 0.022$).
*Conclusion*: In a developing country setting, higher age, larger follow-up, longer distance to travel and being a female child in a family having more than one child are associated with irregularity to hospital visit protocol. At clubfoot clinics, identifying these children and counselling their caregivers might improve compliance.

## EP71

### Lengthening in achondroplasia

Gamal Hosny[1]

[1]Benha Faculty of medicine, Cairo, Egypt

**LEVEL-IV/Limb reconstructions**

Reports of limb lengthening in Achondroplasia have varied greatly in terms of the quality, the evaluation criteria, the patients characteristics, the technique of lengthening [unifocal and bifocal] and the type of fixators used.So,we report a single author experience.
*Methods*: From 1993 to 2011, 77 patients with achondroplasia were treated by bone lengthening due to short stature. Operative technique includes Ilizarov method and transverse lengthening. Hospital stay: 2 days postopertively. Latency period was 7–15 days and the rate of distraction: 1/3 mm every 8 h. Original height ranged from 95 to 131 cm. Age at operation ranged from 5 to 18 years. Follow-up was 2–14 years. Of the 77 patients: 13 completed the protocol, 32 Tibial lengthening alone[unifocal] 24 Tibial and Femoral Lengthening, 8 Tibial lengthening [bifocal]. The patients were evaluated clinically and radiographically including the ROM, functional activities and satisfaction of the patients.
*Results*: Magnitude of lengthening achieved ranged from 5 to 18 cm (average 8.5 cm) in one stage (25–80 % of the original length). Healing Index 39 days/cm. Complications: 1-Somesort of pin track infection in all cases. 2-Common peroneal N in 4 cases which developed after 2 days, 3 months and 5 months consequently. The first case was treated with removal of the Tibio-Fibular wire, the second with retraction of that wire till it become flush with the fibula and the third with decompression. 3-Knee subluxation in one case during Femoral lengthening, treated by extension of the frame to the Tibia and gradual reduction. 4-Fracture of the regenerate in 6 segments in 3 cases, 2 treated with frame reapplication, 2 with percutaneous pinning and casting and 2 with casting. 5. One case. femoral nonunion. 6-Joint stiffness (Mostly after femoral lengthening). Only three patients required quadriceps plasty. TTA was required in eight patients to correct equinus deformity.
*Conclusion*: Ilizarov method for bone lengthening in Achondroplasia is an effective method as none of our cases required bone grafting except one. Maximum magnitude of lengthening in this series was 80 % of the original length.

## EP72

### Forces, growth plate areas and compressive stresses at the proximal femur

Bernhard Heimkes[1], Christian Ziegler[1], Dominik Eibl[1], Tobias Geith[2], Andrea Baur-Melnyk[3]

[1]Department of Orthopaedic Surgery, Muenchen, Germany;
[2]Department of Clinical Radiology, Muenchen, Germany;
[3]Department of Cinical Radiology, Muenchen, Germany

**LEVEL-I/Basic science**

*Purpose*: The proximal femoral is particularly suitable for recognizing principles concerning the enchondral ossification and the longitudinal growth of the skeleton as it holds two morphologic identically structured growth plates of different size and different load. The purpose of the study was to compare the forces, areas and stresses acting on the epiphyseal growth plate and the greater trochanter growth plate during growth.
*Methods*: Pseudanonymised MRI data of 36 hip-healthy children of different age and gender were taken to specify the loads, areas and compressive stresses on the epiphyseal and apophyseal growth plates. The areas were surveyed by means of the software tool OsiriX, the forces were calculated by established numerical modelings.
*Results*: 1. The areas of the two mentioned growth plates steadily increased from infancy until puberty. The epiphyseal growth plate increases 0.8 $cm^2$/year ($\sigma = 0.068$), the apophyseal growth plate 0.5 $cm^2$/year ($\sigma = 0.048$).
2. The epiphyseal growth plate area and the apophyseal growth plate area are at a ratio of 1:0.56. This ratio remains unchanged during the entire growth period ($\beta = 0.012$).

3. Both growth plates are loaded different during growth, but their computed compressive stresses are at a ratio of $\sigma e$:$\sigma a = 1$:1.07 during the entire growth period ($\beta = -0.022$)
*Conclusion*: With respect to the proximal femur you will find the fundamental skeletal remodeling law that growth plates of different size and different load are stressed by identical compressive stresses during the entire growth period. Increasing forces during growth enlarge the growth plates in order to keep compressive stresses constant according to the formula: **σc = force/area = const**.

## EP73

## Thoracic and lumbar spinal fracture treated conservatively in children and adolescent, outcomes in coronal and sagittal planes

Audrey Angelliaume[1], Jérôme Sales de Gauzy[2], Louis Boissière[1], Jean-Roger Pontailler[1], Bouty Aurore[1], Yan Lefèvre[1]

[1]University Hospital Bordeaux, Bordeaux, France
[2]University Hospital Toulouse, Toulouse, France

**LEVEL-III/Spine**

*Introduction*: The authors examined a case series of patients younger than 18 years who had sustained a traumatic spine fracture to evaluate radiological and clinical findings of coronal and sagittal spinopelvic balance, after conservative treatment.
*Materials and Methods*: From 1996 to 2014, a tricentric cohort of 48 patients with an average of 12 years was radiographically reviewed at 50 months. All demographic data and Cobb angle of fractured vertebra and regional Cobb angle were measured. In coronal plane, analyses were done according to initial Risser grade, number of fractures and level of injury. In sagittal plane, 8 spinopelvic parameters were recorded. All measurements were done at the moment of the accident, authors using full spine radiograph with brace or cast and at last follow up with another full spine radiograph without contention; all radiograph were done in standing position.
*Results*: Patients with Risser grade 2 or less have a higher remodeling potential than those with Risser grade 3 or above both in coronal and sagittal plane. In coronal plane, there was a total of 11 scoliosis. In group with Risser grade 3 or above, with a single vertebral fracture and lumbar fracture final regional Cobb angle was statistically higher than initial regional Cobb angle. In sagittal plane, lumbar fractures show 8.7° of residual local kyphosis and have an increased lumbar lordosis at the end of follow-up. Thoracic fracture had a residual local kyphosis at 8.2° with no modification of final thoracic kyphosis. Analyses reported no modification of pelvic parameters except sacral slope in one subgroup.
*Conclusion*: Our study confirms that young patients have a great growth potential to correct post trauma local deformity and that lumbar fracture seems to be responsible for more adaptive responses than thoracic, in both coronal and sagittal plane. Thoracic and/or lumbar spinal fracture is a risk factor of scoliosis in patients younger than 18 years old. In sagittal plane, local kyphosis is compensated not only thanks to remodeling bony power but also thanks to adaptive spinal mechanism in adjacent structures.

## EP74

## Gait adaptations in children with anterior cruciate ligament injury

Monica Ursei[1], Marino Scandella[1], Franck Accadbled[1], Gorka Knorr[1], Caroline Munzer[1], Jérôme Sales de Gauzy[1]

[1]Hôpital des Enfants, Toulouse, France

**LEVEL-IV/Hip/Lower extremity**

*Introduction*: ACL injuries are common in adult population, resulting in knee instability, pain and increased risk of knee osteoarthritis. Several studies have demonstrated changes in gait pattern of subjects with this pathology. Once thought to be rare in a paediatric population, ACL injury has received increased attention recently. ACL tears are becoming more frequent, as children are more involved in contact sports and better diagnostic techniques (such as MRI) are available. Few studies have examined changes in gait pattern of children with ACL injury and gait adaptations related to this pathology. The purpose of our study is to examine gait compensation in children with symptomatic ACL injury.
Materials and Methods: 54 children [16 girls, 38 boys; mean age 14.2 years (range 10–17)] with unilateral ACL injury were recruited from Children's Hospital, Toulouse, France. All of them presented knee pain, different degrees of swelling and knee instability. Diagnosis was confirmed by MRI scan. All the patients with neurological or congenital musculoskeletal condition or previous knee surgery were excluded. Each patient had undergone gait analysis at between 2 and 40 months (mean 9.3 months) after injury. We analysed ankle, knee and hip kinematic parameters and foot progression. Patient data were compared to our gait lab reference normal gait values.
*Results*: Ankle kinematics were statistically different from normal values especially at early stance and mid-swing. 47 (87 %) patients had plantar flexion at initial contact (between 2° and 15°, mean 4.5°). Plantar flexion was increased in terminal stance in 44 (81 %) patients. Ankle dorsiflexion was diminished in swing in 37 patients (68.5 %). We report no significant changes regarding knee kinematics. 36 (66.7 %) patients had increased hip extension in terminal stance. Foot external rotation was increased both in stance (31 patients) and in swing (47 patients)
*Discussion and Conclusion*: Children with ACL injury walk with modified gait pattern: plantar flexion at initial contact, increased plantar flexion and hip extension in terminal stance, increased external foot rotation in stance and swing. Modified ankle and hip kinematics seem to be an adaptation to avoid knee instability. Understanding gait changes in children with symptomatic ACL injury can be useful in adapting its management.

## EP75

## The Fitbone®-system: a concept for limb lengthening and deformity correction

Rainer Baumgart[1]

[1]ZEM-Germany, Munich, Germany

**LEVEL-IV/Limb reconstructions**

The Fitbone®-System is a fully implantable device based on an external computerized control unit and a wireless energy transmission to a motorized distraction nail. The system is not only a lengthening device but also a multifunctional correction tool for posttraumatic and congenital deformities and bone defects. Which results can be expected?

Worldwide about 2,500 implantations of the Fitbone-System were done in 13 countries, 1,200 of them in our Center in Munich. 47 % of our patients were posttraumatic cases, 33 congenital cases and 13.5 % bilateral cases due to dwarfism or cosmetic reasons. The medium lengthening amount was 39 mm (range: 20-85 mm). Bone transport was performed in 3.6 % of the cases and combined with lengthening in another 2.9 %.

660 patients which were treated with 719 Fitbone implants were evaluated for quality control with LSR before and after finishing treatment. In 628 cases the device was used for lenthening with or without axis and torsion correction (58 % femur, 42 % tibia). The mean lengthening amount was 41 mm (20–85) at the femur and 35 mm (20–60) at the tibia. In 32 cases bone transport was performed. 94 % of the lengthenings and 97 % of the bone transports were finished sucessfully (±5 mm). The mechanical axis was within the pysiological range (±5 mm at the knee joint level) in 89 % of the lengthening group. 4 % of the patients had a slight varus and 7 % had a slight valgus of <5°. There was no clinical relevant torsion deviation. The range of motion of the knee and the ankle joint was reduced temporarely, especially when femur and tibia were lengthened simultaneouly but recovered in all cases exept one ankle joint contracture at the time of follow up.

Considering the developed operation technology made for the Fitbone-device, axis deformities and deviations of torsion are able to be acutely corrected intraoperative while lengthening can be precisely performed computer controlled postoperatively. Technical problems during the time of early development could be diminished completely. In our study there was not a single implant related failure due to the high technical standard which was reached meanwhile. In conclusion the Fitbone-System is an efficient, safe, comfortable and reliable tool for exact corrections of all geometric parameters of the lower leg. The operative technique is sophisticated but justified by the precise outcome and the excellent cosmetic result.

## EP76

### Femoral overgrowth in children with congenital pseudarthrosis of the tibia (CPT) with special reference to associated factors and its developmental patterns

Mi Hyun Song[1], Tae-Joon Cho[2], Won Joon Yoo[2], In Ho Choi[2]

[1]Jeju National University Hospital, Jeju, Republic of Korea
[2]Seoul National University Hospital, Seoul, Republic of Korea

**LEVEL-IV/Trauma/Upper extremity**

*Purpose*: We have observed a tendency of femoral-length overgrowth of the affected limb in children with CPT. The intention of this study was to identify associated factors with femoral overgrowth, and to determine the developmental patterns of femoral overgrowth in children with CPT.

*Methods*: The data on 28 children (10 boys and 18 girls) with atrophic-type CPT, who were treated by the Ilizarov method, were retrospectively reviewed. Either primary osteosynthesis alone in 9 children (Group 1), or distal osteosynthesis plus proximal lengthening of the tibia/fibula by distraction osteogenesis (DO) in 19 children (Group 2) was performed at an average of 4.5 years (1.7–7.3). Children in Group 2 underwent a total of 30 rounds of DO in the affected tibia/fibula. Serial slit scanograms and standing teleradiograms were taken to determine lower limb-length inequality in all children during follow-up (average 10.6 years, range 4.4–18.3 years). Femoral overgrowth was defined as a femoral segment of the affected limb was longer than 10 mm or more relative to that of the contralateral normal limb. To identify factors associated with femoral overgrowth, several candidate variables including, presence or absence of neurofibromatosis, deformity severity, the extent of tibial shortening, increased femoral neck-shaft angles, treatment with or without DO, and re-fracture were evaluated. We also determined the developmental patterns of femoral overgrowth according to Shapiro’s classification.
*Results*: At last follow-up, 10 children (36 %) presented an average of 13 mm (10–18) of femoral overgrowth. Tibial and fibular lengthening by DO was significantly associated with femoral overgrowth ($p = 0.049$), whereas other remaining factors turned out to be insignificant. There was significant difference in femoral-length overgrowth between Group 1 and Group 2. Interestingly, all ten children with femoral overgrowth showed an increased femoral neck-shaft angle of the affected limb. The developmental patterns of femoral-length overgrowth were diverse and varied among individuals, when assessed according to Shapiro’s classification.
*Conclusion*: This study demonstrates that tibial and fibular lengthening by DO can readily contribute to ipsilateral femoral overgrowth in diverse developmental patterns in children with CPT. It is conceivable that increased blood flow during distraction, together with unknown multiple factors, could influence on the speed of femoral growth.
*Significance*: Taken that femoral overgrowth of the affected limb is not infrequently observed during tibial and fibular lengthening in children with CPT, this point should be taken into consideration for lower limb-length equalization.

## EP77

### Bioexpandable prostheses: new perspectives after resection of malignant bone tumours in children

Rainer Baumgart[1]

[1]ZEM-Germany, Munich, Germany

**LEVEL-IV/Tumours**

Expandable endoprostheses could be an option after resection of malignant bone tumors of the lower extremities in children and adolescents not only to bridge the defect but also to overcome limb length discrepancy. To achieve equal limb length at maturity new concepts of expandable endoprosthesis are now available. Which results can be expected?

One option is to lengthen the prosthesis with an internal power unit but especially in cases of a huge demand of lengthening the relationship from prosthesis to the remaining bone becomes worse. As consequence a new idea was to lengthen not the prosthesis but the remaining bone as it is performed frequently in cases of congenital or posttraumatic limb length discrepancies. Both systems (MUTARS Xpand and MUTARS BioXpand, implantcast, Buxtehude, Germany)

are equipped with a motor drive which gets its energy wireless from outside by electromagnetic waves. After surgery the skin is closed completely. In case of bone lengthening with the "bioexpandable" endoprosthesis, the relationship of the prosthesis length compared to leg length develops in favour of the remaining bone.

5 patients (3 male, 2 female) were treated with the BioXpand. In 4 cases the femur and in 1 case the tibia was lengthened after resection of an osteosarcoma (3) or an Ewings-sarcoma (2). The mean age of the patients was 16.8 years and the mean amount of lengthening was 78 mm. In all cases limb length equality could be achieved, in one case lengthening was performed in two steps.

There was no infection and lengthening could be finished in all cases. The bone regenerate in the tibia case was poor so that bone grafting had to be performed from the iliac crest. In one cases a temporarily contracture of the knee joint was observed which recovered completely after finishing lengthening. In one case a breakage of the nail occurred 2 years after lengthening just before the replacement to the final prosthesis was planned.

The "bioexpandable" prosthesis is a favourable option for children after tumour resection. The device is safe and offers the same advantages as in congenital or posttraumatic shortenings and better long term stability of the prosthesis can be expected. The small diameter and the length of the remaining bone as well as stress shielding are basic problems, also the repeated operative interventions are a challenge for further developments.

## EP78

### Characteristics of dysplastic acetabulum in neurogenic hip disease with three-dimensional computer evaluation

Marek Jozwiak[1], Bartosz Musielak[1], Po Jung Chen[1], Maciej Idzior[1]

[1]Dega Hospital, Poznan, Poland

**LEVEL-III/Cerebral Palsy**

*Background/Objectives*: The hip dysplasia is a common finding in cerebral palsy patients. The evaluation of hip morphology nowadays is mainly based on X-Ray and two-dimensional computed tomography (CT). However, the accuracy of using only two-dimensional images to claim the three-dimensional structure of hip is doubtful. A more precise three-dimensional image study is essential to help orthopedic surgeons understand comprehensively the true morphology of dysplastic acetabula in order to make a proper treatment decision. This study presents the morphological differences between healthy and dysplastic acetabula by using the reverse-engineering technique to reconstruct detailed and precise three-dimensional pelvic models.
*Methods*: This project developed a new universal method to determine the acetabular volume, and orientation in both healthy and dysplastic acetabula. The DICOM format CT images were used to perform the three-dimensional reconstruction of the geometry of the pelvis. With the use of methods of reverse engineering and rapid prototyping (ScanIP, Rhinceross) measurements based on the 3D reconstructions were carried. For the orientation assessment new, stable reference plane was used (the Base of the Sacrum). Based on it ten cases of neurogenic hip dislocation (GMFCS IV and V) were evaluated in means frontal (inclination angle), horizontal (anteversion angle) and saggital (tilt angle) orientation and were compered with the average values.
*Results*: Children with Neurogenic Hip present completely reversed orientation of the acetabulum. Significantly higher is inclination angle (>90°, whereas in healthy individuals 71°), anteversion and tilt angle is reversed (in neurogenic cases retroversion and posterior tilt, in healthy individuals 31° of anteversion and 31° of anterior tilt).
*Conclusions*: The presented method of measurements is precised tool for assessment of 3D morphology of the acetabulum in not only neurogenic hip disease, but in all paediatric hip pathologies.
In children with GMFCS IV and V it is common to find completely reversed orientation of the acetabulum, what may be very challenging during reconstructive surgeries of this hip

## EP79

### Symmetry of the vertebrae and pedicles in the true transverse plane in adolescent idiopathic scoliosis

Rob Brink[1], Tom Schlösser[1], Dino Colo[1], Marijn van Stralen[1], Jack Cheng[2], Koen Vincken[1], René Castelein[1]

[1]UMC Utrecht, Utrecht, The Netherlands
[2]Prince of Wales Hospital, Shatin, Hong Kong

**LEVEL-II/Spine**

*Background Context*: Studies on transverse plane asymmetry in adolescent idiopathic scoliosis reported relatively longer and thinner pedicles on the concave side of the spinal curvature as compared to convex. Based on these data, it has been suggested that AIS is the result of an active asymmetrical growth process of the vertebrae. The existence of transverse asymmetry of the pedicles in AIS, however, remains controversial and to the best of our knowledge, transverse vertebral body morphology has not been studied before. This study determined the asymmetry in both the vertebral body and pedicle in the true transverse plane in AIS patients and compared it with normal spinal anatomy.
*Methods*: Vertebral body and pedicle asymmetry from T4 to L5 were evaluated on computed tomographic scans of 77 AIS patients and 32 controls using semi-automatic analysis software. Scans were acquired for navigation purposes. Vertebral body symmetry was defined by a dice coefficient (the percentage of overlap between the segmentation of the left and right side of each endplate). Pedicle asymmetry was determined by comparing the width, length of the pedicle and length of the pedicle screw trajectory.
*Results*: Small asymmetry was observed in the vertebral bodies and pedicles of both cohorts. However, there was significantly more asymmetry in AIS patients than in controls in terms of vertebral body symmetry (96.0 ± 0.6 vs. 96.6 ± 0.4 %), pedicle length (−6.2 ± 5.7 vs. −3.0 ± 3.9 %) and screw trajectory length (−5.0 ± 3.4 vs. −2.6 ± 2.3 %). Overall, there was no significant difference in pedicle width between the cohorts. Each vertebra separately, the vertebral asymmetry was more pronounced in the lumbar vertebrae and the pedicle asymmetry more in the thoracic vertebrae. Pedicle asymmetry was most pronounced around the apex, in which the concave pedicle was thinner and longer, but did not correlate with axial rotation.
*Conclusions*: Vertebrae and pedicles of AIS curves are almost as symmetrical as the normal spine, except in the apex. This suggests a limited role for active (asymmetrical) skeletal growth in the etiopathogenesis of AIS.

## EP80

### The impact of clubfoot treatment on parents and caregivers of affected children: a comparison of two urban populations in Europe and Africa

Francesc Malagelada[1], Saadia Mayet[2], Greg Firth[2], Manoj Ramachandran[1]

[1]Royal London Hospital, London, UK
[2]Chris Hani Baragwanath Hospital, Johannesburg, South Africa

**LEVEL-IV/Club-foot**

*Introduction*: With the Ponseti treatment method established as the gold standard, children with clubfeet face a prolonged treatment regimen that might impact on their families. We aimed to determine how Ponseti treatment influences the lives of parents and caregivers and what coping strategies are used by them during the casting and bracing phases of treatment. Secondarily, we aimed to identify any potential differences between two urban referral centres for clubfoot: The Royal London Hospital, London (United Kingdom) and Chris Hani Baragwanath Academic Hospital, Johannesburg (South Africa).
*Materials and Methods*: A total of 70 parents of children affected with idiopathic clubfoot were recruited and included in two groups: one from the United Kingdom (UK) and the other from South Africa (SA). All children were in the bracing phase of treatment and were under 5 years of age. The participants were asked to complete the following three instruments twice, once for the serial casting, and again for the bracing phases of treatment: the Impact On Family Scale (IOFS), the Multidimensional Scale of Perceived Social Support (MSPSS), and the Brief COPE.
*Results*: The mean age of affected children at the time of the study was 24.2 months. A mean of 7.69 serial casts were applied and percutaneous tenotomy was performed in 80 % of cases. The results of IOFS and MSPSS were 29.6 and 63.7 respectively during the casting phase for the UK cohort, versus 32.4 and 69.9 for the SA cohort ($p > 0.05$). During the bracing phase, the IOFS was significantly lower for both cohorts, being 27.1 for the UK ($p = 0.004$) and 28.3 for the SA ($p = 0.006$). MSPSS results did not differ significantly between treatment phases. The SA population used coping strategies at a significantly higher level when compared to the UK population ($p < 0.05$) throughout the treatment phases. Excellent Pirani scores were seen after treatment with minimal complications in both centres.
*Conclusions*: This is the first study to show that the treatment of clubfoot in children causes an impact on family function. This impact is significantly higher during the casting than the bracing phase in the studied populations. In SA, coping strategies are used more than in the UK to deal with the stressful treatment circumstances.

## EP81

### Proximal migration of fibular malleolus during tibial lengthening despite syndesmotic screw fixation: a series of 22 cases

Dimitri Camus[1], Franck Launay[2]

[1]Hopital Timone Enfant, Marseille, France
[2]Hopital Timone Enfant, Marseille, France

**LEVEL-IV/Limb reconstructions**

*Introduction*: During tibial lengthening procedures, it is recommended to prevent fibular malleolus proximal migration using a distal tibiofibular syndesmotic screw, which is removed at 6 months. We have observed proximal migrations of the fibular malleolus despite placement of this syndesmotic screw.
*Objective*: The objective of this study was to demonstrate this migration and to study the influence of two factors that may favor its occurrence: positioning of the syndesmotic screw and union of the fibula at the time of removal.
*Hypothesis*: An unhealed fibula at the time the distal tibiofibular syndesmotic screw is removed and its tricortical position promote the proximal migration of the fibular malleolus.?
*Materials and Methods*: This was a retrospective, single-center, analytical study that included 22 length- ening procedures in 18 patients from 5 to 17 years of age who had undergone tibial lengthening and presented a preoperative continuous fibula. The position of the fibular malleolus, union of the fibula, and the tri- or quadricortical position of the screw were assessed based on four successive X-rays.
*Results*: Tricortical positioning of the syndesmotic screw was significantly associated with proximal migration of the fibular malleolus during lengthening ($P = 0.0248 < 0.05$). However, there was no significant relation between an unhealed fibula and proximal migration of the fibular malleolus when the screw was removed ($P = 0.164 > 0.05$).
Discussion: Proximal migration of the fibular malleolus during lengthening is promoted by placing a non-quadricortical syndesmotic screw. Quadricortical positioning of the screw should be recommended. Migration of the fibular malleolus after ablation of the syndesmotic screw seems to be related to absence of fibular union but this series was too small to demonstrate this clearly.

## EP82

### Brain damage results in more collagen in spastic biceps brachii muscle compared to control: a contribution to contracture formation?

Eva Pontén[1], Ola Gremark[1], Lars Björk[1], Stefan Gantelius[1]

[1]Karolinska Institutet, Stockholm, Sweden

**LEVEL-III/Trauma/Upper extremity**

*Introduction*: Children with upper motor neuron lesions (UMNL) with spasticity, such as cerebral palsy (CP) often develop contractures and muscle stiffness. This is often explained as that the muscles do not grow with the same pace as the bone. We wanted to explore if there is more to the contracture—is the muscle tissue itself changed? Around each muscle fiber and around muscle fiber bundles there is supportive collagen containing extracellular matrix (ECM), the endomysium and perimysium, which play an important role in force transduction and passive resistance. Is there an increased amount of ECM in spastic muscle compared to controls? What is the location of the ECM in spastic muscle?
*Materials and Methods*: We compared the biceps brachii muscle in children and adolescents with an UMNL, who due to elbow flexion contractures were planned for biceps tendon lengthenings, (n = 20, mean age 16 years), with age and sex-matched healthy controls who had had an accidental death (n = 10, mean age 15 years, range 7–21 years). Of the children with UMNL, 18 had CP, and 2 had sustained a brain damage later during childhood. The specimens were snap frozen, cross-sectioned and stained with Sirius Red, which stains collagen red. The perimysium and the endomysium were differentiated using polarized light in a Leica microscope. Images were captured by a digital camera (Leica DFC320). The percentage of the cross sectional area of the muscle occupied by the perimysium was calculated using image analysis (Leica Qwin), and compared using Mann–Whitney–Wilcoxon non-parametric test.
*Results*: The perimysium, surrounding muscle fiber bundles, was thickened in the spastic muscle, resulting in an increased total percentage of ECM to 6.4 % +2.0 SD, compared to 5.04 % +1.2 SD in control muscle, $p < 0.03$. The ECM around each muscle fiber was, however, not different from control.
*Significance*: Stiffness of spastic muscles could, to a large part, be caused by an upregulation of collagen in the perimysial extracellular matrix. This could be the reason that cutting of intramuscular fascial strokes in the muscle often is a very effective treatment of contractures, lengthening the myotendious complex.

## EP83

## Surgical treatment of hand enchondromas in multiple enchodromatosis

TIphanie Delcourt[1], Celine Klein[1], Arielle Salon[2], Georoges Finidori[2], Christophe Glorion[2], Stephanie Pannier[2]

[1]Hopital Necker Enfant Malade, Paris, France
[2]Hopital Necker Enfant Malade, Paris, France

**LEVEL-IV/Tumours**

*Purpose*: Multiple enchondromatosis (Ollier's disease) is a rare disease characterized by the development of multiple cartilaginous tumors responsible for bony deformations often appearing during the first decade of life. The treatment of metacarpal and phalangeal localizations usually comes secondarily after the treatment of leg deformities. The purpose of this study was to evaluate the results of the surgical treatment of hand lesions in children.
*Methods*: In this retrospective monocentric study, ten patients presenting Ollier's diseas were operated on for metacarpal or phalangeal enchondromas since 1990. Thirty-five enchondromas were operated by curettage and corticoplasty, nine enchondromas had an additional bone graft. The patients were operated on at mean age 10.5 (5–18 years old). We analyzed the global functional result, the finger's mobility, radiological bone filling and recurrence according to Tordai classification and the existence of complications at mean follow up of 49 months (87 months).
*Results*: At last follow up, all the patients had a satisfying functional result and complete mobility of the digit. Bone filling was staged Tordai 1 or 2 for 33 enchondromas. One patient presented a radiological recurrence in three enchondromas that needed a second curettage. There were three complications: one partial epiphysiodesis responsible of clinodactylia that needed reoperating on 2 years later, one hematoma with secondary asymptomatic calcification, and one hyperlaxity with "col de cygne" deformity. No malignant transformation was observed. There were no differences of results between simple curettage and curettage associated with bone graft.
*Conclusion*: Few studies have analyzed the management of hand lesions in Ollier's disease. Our results show that the surgical treatment of metacarpal and phalangeal enchodromas gives a satisfying functional result. At medium rang we observed radiological filling of the tumors and osseous remodeling. This study shows that the surgical treatment of hand enchodromas in Ollier's disease should be considered.

## EP84

## The revised FLACC behavioural pain scale: reliability and validation for pain assessment in children with cerebral palsy

Line Kjeldgaard Pedersen[1], Ole Rahbek[1], Lone Nikolajsen[1], Bjarne Møller-Madsen[1]

[1]Aarhus University Hospital, Aarhus, Denmark

**LEVEL-II/Cerebral Palsy**

*Purpose*: Assessment of pain in children with cerebral palsy (CP) is a challenge since they might not be capable of using a self-rating assessment tool; hence an observational or behavioral pain score is needed. The r-FLACC behavioral pain scale has only been assessed for reliability by the developers of the score. The aim of this study is to make an independent evaluation of the reliability and validity of r-FLACC for pain assessment in children with (CP).
*Methods*: Twenty children with CP undergoing orthopedic surgery were included. The parents assessed the pain intensity with the Observational Visual Analog Scale and a 2 min standardized video recording was made for r-FLACC scoring. Two nurses r-FLACC scored the recordings independently. Ten of the recordings were reviewed again 1 year later.
*Results*: Reliability was supported by three measurement properties. Internal consistency was excellent with a Cronbachs alpha of 0.9278 and 0.9758. A test–retest showed excellent intra-rater reliability with a Spearman Correlation of 0.0040 and an intra-class correlation (ICC) of 0.97530. Inter-rater reliability was acceptable with a Spearman Correlation of 0.000 and an ICC of 0.74576. Validity was supported by three measurement properties. Construct validity was supported by a significant increase in r-FLACC scores following surgery ($p = 0.0427$). Criterion validity was acceptable with Pearson's Correlation Coefficients of 0.75 and 0.59 when comparing the r-FLACC scores and the VAS-OBS scores.
*Conclusions*: The r-FLACC behavioral pain scale has high reliability and validity and may be considered as gold standard for pain assessment in children with CP.

## EP85

## How successful is closed reduction of the dislocated hip after failed Pavlik harness or ring splint treatment?

David Spence[1], Elaine Robinson[1], Aidan Cosgrove[1]

[1]RBHSC, Belfast, UK

**LEVEL-III/DDH**

*Purpose*: The aim of this study was to determine the success rate of closed hip reduction under general anaesthetic following a failed trial of Pavlik harness or ring splints in children with developmental dysplasia of the hip (DDH).

*Methods*: This is a retrospective review of all children with a dislocated hip undergoing closed reduction in the years 2007–2012 in Northern Ireland. Data was obtained from the Northern Ireland DDH database, hospital notes and electronic care records. Children included in the study were those with a dislocated hip who failed a trial of Pavlik harness or ring splint treatment. Exclusion criteria included children with neuromuscular disorders and children that proceeded directly to closed reduction without any preceding splintage. All radiographs of successful reductions were reviewed and the presence of AVN was recorded.
*Results*: There were 1,362 hips in 1,073 children treated in Pavlik harness or ring splints. 47 hips in 42 children failed to stabilise in Pavlik harness or splint and proceeded to arthrogram and closed reduction under anaesthesia with adductor tenotomy, if required. Mean age at time of procedure was 182 days. 11 hips in 10 children failed to reduce under anaesthetic and later underwent open reduction. The remaining 36 hips in 32 children had successful closed reductions in theatre with application of hip spica in the human position of Salter. Of the 36 deemed successfully reduced on arthrogram, 8 hips in 6 children were found to be dislocated or subluxed on post operative CT scan or on follow up, for these children treatment in spica was abandoned, mostly within the first few days. Therefore out of the initial group of 47 hips, 28 hips in 26 children (60 %) had successful closed reductions. At a mean of 2.9 years radiographic follow up, 66 % (18) of these hips had no evidence of AVN and 26 % (7) type I AVN with one type II and one type IV AVN changes. One patient has had a pelvic osteotomy for residual dysplasia.
*Conclusion*: For hips that failed to stabilise in Pavlik harness or ring splints, we had a 60 % success rate for arthrogram and closed reduction. Therefore following failed Pavlik harness or ring splint treatment, we continue to advocate, proceeding to arthrogram with the potential for closed reduction before considering open reduction.

## EP86

### Cumulative radiation exposure with EOS® imaging compared to standard spine radiographs

Anthony Stans[1], T David Luo[1], Beth Schueler[1], A Noelle Larson[1]

[1]Mayo Clinic, Rochester, USA

**LEVEL-III/Spine**

*Purpose*: EOS® is a slot-scanning X-ray system designed to reduce radiation exposure in orthopaedic imaging. There are few independent studies comparing EOS® PA, AP, and lateral imaging versus conventional projection radiographs for children with spinal deformity and most report skin entrance dose. We sought to estimate the total radiation exposure to scoliosis patients during the course of treatment using standard imaging techniques versus EOS® PA and AP views.
*Methods*: Forty-two skeletally immature idiopathic scoliosis patients were treated with bracing (21) or spinal fusion (21) and were followed to skeletal maturity. The number of scoliosis radiographs (PA and lateral) for each patient was recorded. A computerized dosing model was used to calculate estimated patient and organ doses for PA and lateral scoliosis X-rays taken with EOS®, computed radiography with and without filter (CR and CRF). At this time, our EOS system did not have a copper filter. Assuming that each X-ray taken delivered the same radiation as the phantom calculation, we estimated the total effective and organ dose that each child would have received using EOS®, CR, or CRF. Annual background radiation is 3 mSv.
*Results*: Mean number of radiographs per patient was 20.9 (range 8–43). Patients who underwent surgical treatment had a significantly greater number of X-rays than patients who were braced (27.3 vs. 14.5, $p < 0.001$). Assuming all films were CR, the mean cumulative dose is estimated at 5.4 mSv. With EOS® films, the mean cumulative estimated dose is 2.7 mSv, a decrease of 51 %. An AP versus PA EOS® radiograph results in an 8× higher radiation dose to the breasts and 4× higher dose to the thyroid.
*Conclusions*: The EOS® imaging system moderately reduced the total radiation exposure to skeletally immature scoliosis patients. Over the entire treatment course, use of EOS instead of CR represents 2.7 mSv mean dose reduction (or 0.91 years of background radiation). PA films significantly reduced breast and thyroid dose.
*Significance*: Dosing to the thyroid is four times higher and to the breast is eight times higher with an EOS AP compared to PA film. Compared to CR imaging, our EOS imaging technology (without the copper filter) resulted in a 50 % decrease in total effective radiation dose. This is less reduction than most previous studies on this topic, which simply reported skin entrance dose.

## EP87

### Long-term outcomes and rates of arthroplasty after pediatric surgery for developmental hip dysplasia

Anthony Stans[1], Ernest Young[1], A Noelle Larson[1]

[1]Mayo Clinic, Rochester, USA

**LEVEL-IV/DDH**

*Purpose*: The long-term outcomes of pediatric developmental hip dysplasia are not well-described in the literature. We sought to determine the rates of total hip arthroplasty (THA) and function following surgical treatment of developmental hip dysplasia in a novel cohort of patients with 20+ years follow-up.
*Methods*: Our center treats hip dysplasia in both children and adults. Records were retrospectively reviewed to identify patients who underwent surgery for developmental hip dysplasia prior to age 10. Patients were treated with closed or open reductions, pelvic osteotomies (Salter and Pemberton), femoral osteotomies or a combination of the above. 226 patients met inclusion criteria. Patients with syndromes and neuromuscular disorders were excluded. Follow up information was obtained from mailed surveys and/or the medical record.
*Results*: Of the 226 patients, recent follow-up information was available for 96 dysplastic hips (85 patients) at a mean 29 years follow-up. Of these, 33 were treated with open or closed reduction alone, 46 were treated with pelvic osteotomies (43 Salter, 3 Pembertons), 37 of which only had pelvic osteotomy and 9 with combined pelvic and femoral osteotomies. 17 patients had only femoral osteotomies. Mean age at treatment was 2.6 years old. Of the 96 hips, 21 had gone on to THA at a mean of 26 years after surgery (mean patient age, 32 years old). In addition, 12 patients needed additional surgery (pelvic or femoral osteotomy) for residual dysplasia, and five underwent periacetabular osteotomy as an adult. No patients in the open or closed reduction subgroup went on to arthroplasty. THAs were performed in 7/17 (41 %) of hips treated with femoral osteotomy alone, 11/37 (30 %) of patients treated with pelvic osteotomy alone, and 3 (33 %) of hips treated with combined pelvic and femoral osteotomy. Using a Kaplan–Meier survival analysis for only the pelvic osteotomy patients, rate of THA was 22 % at 20 years of follow-up and 40 % at 30 years of follow-up.

*Conclusions*: Total hip arthroplasty was a common outcome in patients who had pelvic and/or femoral osteotomies in childhood for developmental hip dysplasia. Our study is limited by patient loss to follow-up. However, at mean of 29 years after pediatric surgery for DDH, more than 30 % of those patients located had gone on to joint replacement.

## EP88

## Congenital and chronic traumatic radial head dislocation: the results of osteotomy using 3-dimensional artificial bone models

Hui Taek Kim[1], Tae Young Ahn[1]

[1]Pusan National University, Busan, Republic of Korea

**LEVEL-III/Limb reconstructions**

*Purpose*: Chronic radial head dislocation in children is often difficult to treat due to the complexity of the bony deformity. Therefore simple correction of the deformity based on 2-dimensional (2D) radiographs may not be enough for good results, especially in congenital cases. The purpose of this study was to see if 3-dimensional (3D) models were more instructive than traditional 2D images for planning osteotomy cuts.
*Materials and Methods*: Five patients who had unilateral radial head dislocation and who had 3DCTs taken before and after surgery were selected. Dislocation was traumatic in 2 and congenital in 3. Mean age at the time of surgery was 11.8 years. 2D images were converted to 3D data. 3D bone models were produced using Poly Jet 3D Printer. Two preoperative and two postoperative 3D bone models were made for each patient. Likewise, bone models of five normal forearms were also produced. We used a silicon band to connect the radius and ulna, as an annular ligament and for the distal radioulnar joint, and silicon sheet for the interosseous membrane. For simulation of surgery, osteotomies were performed on another two preoperative models for each patient in the ulna (at one level) and/or radius (at one or two levels) using the normal bone models as a reference. The osteotomies were fixed with AO plates. We measured rotational arc for all four bone model groups: (1) normal, (2) patient's preoperative, (3) patient's postoperative, and (4) simulated (osteotomized) model based on patient's preoperative one. The forearm was pronated and supinated by universal testing machine using customized apparatus for rotation along the axis running from the center of the radial head to the center of the distal ulna. Maximal pronation and supination were defined when the radial head began to displaced from the ulna, and these positions were checked by computer from sensors (surface markers) placed on the bones. Mean values of the four groups were compared.
*Results*: Osteotomized (simulated) bone models showed more similar shape to the normal bone models than to the patient's postoperative bone models. Mean rotational arc of the simulated models was significantly greater ($66.8 \pm 20.29$) than our patient's preoperative ($52.4 \pm 24.05$) and postoperative ($60.2 \pm 20.03$) ones ($p < 0.05$).
*Conclusion*: Detailed analysis based on 3D bone models is helpful in understanding the complex deformities involved in chronic radial head dislocation, and leads to improve surgical outcomes by more accurate correction of the deformities.

## EP89

## Lateral ankle instability in choreoathetoid CP patients

Maria Vlachou[1], Stefanos Chatzipantelis[1], Dimitris Dimitriadis[1]

[1]Mitera Children's Hospital, Athens, Greece

**LEVEL-IV/Foot and ankle**

*Purpose*: The study concerns patients with severe uncontrolled choreoathetoid CP, with excessive involuntary muscle contractions of the lower limbs, mostly located at the ankle joints forcing them into plantar flexion and inversion. These stressful attacks inevitably lead to the development of lateral ligamentous failure and consequently to instability and chronic painful arthritic changes, swelling and varus tilt of the talus in both ankles.This new technique aims to provide satisfactory results in terms of stability by reconstruction of the lateral ligamentous complex.
*Methods*: 17 patients with 24 feet showing choreoathetoid CP and lateral ankle instability, underwent the combination of Chrisman–Snook/split brevis graft technique. This is designed to replace both ATFL and CFL and to prevent anterior translation and inversion by restriction of the subtalar motion. In addition a split tibialis posterior tendon transfer laterally to the Peroneus Brevis (Green technique) was added in order to reinforce the weakened peroneal action. The mean age of the patients at the time of the operation was 12 years (11–17 years) and the average follow-up was 5 years (2–8 years). All patients were placed in a short leg cast with the ankle at 90°, in a slight eversion and non weight bearing for 6 weeks. Patients were allowed to return to full activities with ankle bracing an average of 4 months after surgery.
*Results*: The outcome of the procedure was satisfactory in 23 feet as the ankle became stable and symptomless, while the unsatisfactory results occurred in 1 foot. On this foot during the surgical exposure, the Peroneus Brevis was found ruptured and markedly frayed along most of its length, therefore making it inappropriate for use as a ligament. As an alternative, half of the Peroneus Longus was used for ligament reconstruction, upon which the transferred split posterior tibialis was anchored. Inevitably 3.5 years post-op there was a recurrence of lateral ankle instability and the patient underwent a successful ankle fusion.
*Conclusions*: The results obtained suggest that the application of the combination of these two techniques can be beneficial to a young patient at an early stage of choreo-athetosis with lateral ankle instability, thus avoiding joint fusion.
*Significance*: The combination of the Chrisman–Snook procedure with the split tibialis posterior tendon transfer as a new technique has been successfully employed in 24 consecutive cases of chronic ligamentous instability in children and adolescents with choreoathetoid CP with very satisfactory overall results and almost no complications.

## EP90

### Treatment of simple bone cysts in children: a comparison of steroid injection and percutaneous filling with synthetic calcium phosphate ChronOS inject

Simon Ondrus, M.D., Ph.D.[1], Jiri Jochymek, M.D., Ph.D.[1], Ladislav Planka, M.D., Prof.[1], Petr Gal, M.D., Prof.[1]

[1]The University Hospital, Brno, Czech Republic

**LEVEL-II/Other/varia**

*Purpose*: The aim of the study was to compare two methods of simple bone cyst therapy: the most widely used method of repetitive Depo-Medrol applications against the novel method based on mini-invasive percutaneous filling of the cyst with ChronOS inject, a synthetic biocompatible resorbable material. An alternative hypothesis assumed that the new method would result in fewer operations in patients with simple cyst and better treatment outcomes, as evaluated by Neers criteria for bone cyst therapy.
*Methods*: Patients treated for simple bone cysts at the Department of Paediatric Surgery, Orthopaedics and Traumatology between 2001 and 2011 were included. In the 2001–2003 period, 24 patients were treated with Depo-Medrol. Between January 2005 and December 2011, 54 patients with the same diagnosis were treated by minimally invasive application of ChronOS inject.
*Results*: A total of 59 surgical interventions were performed in 54 patients treated by ChronOS inject and 90.7 % cyst healing without the need for additional surgery was achieved. Of the 24 patients treated with Depo-Medrol, 12 patients (50 %) showed cyst healing with no further surgery required. A total of 69 applications were needed.
*Conclusions*: The results showed that, in the patients treated by the ChronOS injection method, the outcomes achieved were significantly better than those in the patients treated with Depo-Medrol. The difference was in the number of operations needed, which were significantly fewer in the ChronOS inject method, as well as in the overall treatment outcome, with significantly more excellent results in the ChronOS inject method.

## EP91

### Types and arrangement of thyroid shields to reduce exposure of surgeons to ionizing radiation during intraoperative use of C-arm fluoroscopy

Byung Chae Jo[1], Chin Youb Chung[1], Kyoung Min Lee[1], Sang Hyeong Lee[2], Dae Gyu Kwon[3], Ki Hyuk Sung[4], Seung Yeol Lee[4], In Ho Choi[5], Tae-Joon Cho[5], Won Joon Yoo[5], Moon Seok Park[1]

**LEVEL-II/Basic science**

*Purpose*: Even though the danger to the thyroid from exposure to radiation is well-known, there are no guidelines for the proper use of thyroid shields. We performed this study to assess how the radiation dose is affected by changes in the types of thyroid shields used, and by the arrangements in which they are worn.
*Methods*: Two photo-luminescence dosimeters were used to measure the dose of scatter radiation arriving at the location of the thyroid in a whole body phantom, in the position of the surgeon. On an operating table beside this set-up, was an anthropomorphic chest-phantom representing a patient, for which treatment with C-arm fluoroscopy was simulated. Radiation doses were measured using three different arrangements of the thyroid shield: (1) worn tightly, (2) worn loosely, and (3) worn loosely with a bismuth masking reagent. Same tests were performed using two kinds of thyroid shield: lead and lead-equivalent. The scatter radiation dose delivered to an unshielded thyroid was also measured.
*Results*: For the lead shield group, radiation doses were measured in three arrangements; worn tightly, worn loosely, and worn loosely with bismuth masking reagent, for which the results were 1.91 ± 0.13, 2.35 ± 0.22, and 1.86 ± 0.13 μSv/min, respectively, For the lead-equivalent shield group, doses were measured for the same three arrangement, for which the results were 1.79 ± 0.12, 1.82 ± 0.11, and 1.74 ± 0.12 μSv/min. The unshielded thyroid group received a radiation dose of 16.32 ± 0.48 μSv/min. Wearing the shield tight, or wearing it loose with a bismuth masking reagent, yielded better results than simply wearing it loosely.
*Conclusion*: The use of some form of thyroid shield is essential during treatment with C-arm fluoroscopy. It was found that the best ways to reduce scatter radiation exposure to the thyroid were wearing the thyroid shield tightly or wearing it loosely in combination with a bismuth masking reagent. Use of a bismuth masking reagent, inter alia, provides comfort to the surgeon's neck with a high radiation shielding property.
*Significance*: The significance of this study is to present guidelines for the proper use of thyroid shields.

## EP92

### Single stage posterior pedicle screw based correction for neglected congenital scoliosis presenting in adulthood

Jae Hyuk Yang[1], Seung Woo Suh[1], Jin Ho Hwang[2]

[1]Korea University, Guro Hospital, Seoul, Republic of Korea
[2]Yonsei University, Severance Hospital, Seoul, Republic of Korea

**LEVEL-III/Trauma/Upper extremity**

*Study design*: Retrospective comparative study.
*Objective*: To analyze the efficacy of single stage posterior approach in adult neglected congenital scoliosis.
*Summary of Background Data*: Recently, posterior only approach has been found to be effective for congenital scoliosis, even in larger and stiffer curves. However effectiveness of posterior only correction in adulthood has not been studied for neglected congenital scoliosis.
*Methods*: Patients who underwent single stage posterior correction for congenital scoliosis, from 2007 to 2012 with minimum follow up of 2 years were selected. Three study groups were identified based on age: Group A (>18 years), Group B (7–11 years) and Group C (11–18 years). Pre-operative and postoperative indices of coronal (Cobb's angle, coronal balance, T1 angle, and clavicular angle) and sagittal (spinal vertical axis, kyphotic angle, and lumbar alignment) alignment were analysed. Pulmonary function, surgical time, intraoperative blood loss, hospitalization time, ICU admission and complications were reviewed.
*Results*: 9 patient of Group A formed the neglected congenital scoliosis group. The follow up period was 55.4 ± 33.3 months. Osteotomy was done in all, with thoracoplasty in 5 cases. Cobb's angle changed form 75° ± 18° to 37° ± 18° with a correction rate of 53 %. Group B and C did not show differences in terms of type of scoliosis, gender ratio, and preoperative Cobb's angle ($p = 0.08$, 0.834, 0.259, respectively) in comparison to Group A. Group A and C did not show any statistically significant differences for Correction rate ($p = 0672$), however, Group B showed significant high correction rate than Group A and B ($p < 0.001$, $p = 0.019$, respectively). Amount of blood loss in Group A and C were significantly larger than that of Group B ($p = 0.015$). Pulmonary complications were significantly higher in Group A ($p = 0.007$).
*Conclusion*: Posterior only approach achieved adequate correction in adult neglected congenital scoliosis patients. However, the correction rates were better and complications were significantly decreased when intervened at a younger (<11 years) age than later.
Keywords: Spine; Congenital Scoliosis; Posterior approach; Pedicle screw; Osteotomy

## EP93

## Allobone graft behavior after calcaneal lengthening

Moon Seok Park[1], Kyoung Min Lee[1], Soon-Sun Kwon[1], Myung Ki Chung[1], Sang Hyeong Lee[2], Dae Gyu Kwon[3], Ki Hyuk Sung[4], In Ho Choi[5], Tae-Joon Cho[5], Won Joon Yoo[5], Chin Youb Chung[1]

**LEVEL-III/Foot and ankle**

*Purpose*: Calcaneal lengthening with allograft is frequently used for the treatment of symptomatic planovalgus deformity. However, the behavior of an allobone graft after calcaneal lengthening and the risk factors for graft failure are not well documented. Therefore, the purpose of this study was to investigate allobone graft behavior and to evaluate the risk factors for graft failure.
*Methods*: We included patients age ≤20 years who underwent calcaneal lengthening for planovalgus deformity and who had pre- and postoperative antero-posterior and lateral foot radiographs. Three indices of the Goldberg scoring system were chosen for demonstration of allobone graft behavior. A score <6 after 6 months was defined as graft failure. Age, sex, diagnosis, and graft material were evaluated as possible risk factors and a generalized estimating equation was used to assess risk factors for graft failure.
*Results*: A total of 304 feet (176 cases) were included in this study and 961 foot radiographs were assessed. The mean age of the patients at the time of surgery was 11.3 ± 3.4 years, and the mean follow-up was 1.6 ± 1.7 years. The mean estimated Goldberg score was 6.15 at 6 months after calcaneal lengthening, but 11 feet (3.6 %) had a score of <6 after 6 months. Four feet (1.3 %) underwent a reoperation using iliac autologus graft due to pain and loss of correction. Graft material ($p = 0.038$) and older age ($p = 0.006$) were found to be a significant risk factor for graft failure.
*Conclusion*: Graft failure can occur after calcaneal lengthening using allograft. Our study showed that tricortical iliac allobone graft was superior to patellar allobone graft. Physicians should consider the possibility of graft failure when using allobone grafts in older patients.
*Significance*: This study was performed to investigate allograft behavior that could possibly affect graft failure and to evaluate the radiographical amounts of correction after calcaneal lengthening.

## EP94

## Reliability and validity of the Duncan–Ely test for assessing rectus femoris spasticity in patients with cerebral palsy

Moon Seok Park[1], Kyoung Min Lee[1], Byung Chae Jo[1], Sang Hyeong Lee[2], Dae Gyu Kwon[3], Ki Hyuk Sung[4], Seung Yeol Lee[4], In Ho Choi[5], Tae-Joon Cho[5], Won Joon Yoo[5], Chin Youb Chung[1]

**LEVEL-I/Cerebral Palsy**

*Purpose*: The aim of this study was to estimate the interobserver reliability and the convergent validity of the Duncan–Ely test quantitatively by comparing it with 3-dimensional (3D) gait analysis.
*Methods*: This study included 36 consecutive ambulatory patients with cerebral palsy who underwent preoperative 3D gait analysis. The Duncan–Ely test was performed during three different velocities (slow, gravity, and fast). The interobserver reliability was assessed by three examiners. The results of the Duncan–Ely test were compared with kinematic variables derived from the 3D gait analysis to assess the sensitivity and specificity of the test.
*Results*: The intraclass correlation coefficient (ICC) measuring interobserver reliability of the Duncan–Ely test showed the highest ICC value during fast velocity (0.819). The sensitivity and specificity of the Duncan–Ely test during gravity velocity for knee range of motion total were 63.0 and 100 %, respectively, with a cutoff value of 78.3°. The sensitivity and specificity of the Duncan–Ely test during fast velocity for knee range of motion total were 66.7 and 100 %, respectively, with a cutoff value of 65°.
*Conclusion*: In terms of reliability and validity, the Duncan–Ely test is a useful physical examination modality for the preoperative evaluation of rectus femoris spasticity in patients with cerebral palsy.
*Significance*: There is a lack of research regarding the reliability and validity of the Duncan–Ely test. Therefore, the present study aimed to evaluate the interobserver reliability and validity of this test. The ICC, which measured the interobserver reliability of the Duncan–Ely test, showed the highest ICC value during fast velocity (V3). Sensitivity and specificity of the Duncan-Ely test during fast velocity (V3) was highest with a cutoff value of 65°.

## EP95

### Three-dimensional reconstruction and measurement of femoral anteversion from uncalibrated biplanar X-ray images in patients with cerebral palsy

Kibeom Youn[1], In Hyeok Lee[2], Chin Youb Chung[2], Kyoung Min Lee[2], Moon Seok Park[2], Jehee Lee[1]

[1]Seoul National University, Seoul, Republic of Korea
[2]SNU Bundang Hospital, Seong Nam, Republic of Korea

**LEVEL-II/Cerebral Palsy**

*Purpose*: Although computed tomography (CT) provides benefits in accurate diagnosis of femur deformity, there are concerns on potential side effects of radiation exposure. To reduce radiation dose while maintaining the accuracy of diagnosis, reconstructing a 3D shape model from a small number of X-ray images has been studied. Our clinical interest is determining femoral anteversion of patients with cerebral palsy using uncalibrated X-rays taken from AP and LAT views that are not necessarily orthogonal to each other. Current 3D reconstruction methods require calibrated X-rays which uses a calibration object to decide the relative position and orientation of X-ray detection planes. In case of no calibration object is available, self-calibration techniques were studied given a set of corresponding feature points on the X-ray images, which serves as an alternative to a calibration object. It is difficult to exploit self-calibration techniques for femur reconstruction because femurs do not have sharp features that can be identified clearly in two different views. We propose a new reconstruction method that solves for X-ray view calibration and 3D shape reconstruction simultaneously based on advanced optimization algorithms, which do not require corresponding feature points on X-ray images.
*Methods*: Each X-ray image is aligned to match the 3D model's silhouette and the 3D shape is deformed to minimize silhouette matching error iteratively. A statistical shape model built from the femurs of 54 patients with cerebral palsy was used for modeling shape deformation. We use CMA-ES optimization to find global optima and additional manual marking of anatomical point helps avoiding local optima. The center of the femur head, greater trochanter, lateral and medial condyles are roughly marked and the correspondence of those points between X-rays is not needed.
*Results*: Thirty-six hips (18 patients) are evaluated for the measurements of femoral anteversion. The mean age is 12 (range 7–18), and average ante version is 33° (range 7°–61°). 3D reconstructed femurs obtained using the proposed method from uncalibrated biplanar X-ray images show excellent concurrent validity($R = 0.975$, $p < 0.001$) with average 3.3° angle difference (range 0.0°–6.7°).
*Conclusion*: The measurement of femoral anteversion with the proposed method using uncalibrated biplanar X-ray images showed excellent concurrent validity in patients with cerebral palsy.
*Significance*: This study proposes a method to measure femoral anteversion from uncalibrated X-rays by solving for X-ray view calibration and 3D shape reconstruction simultaneously.

## EP96

### Effects of reduction in the alpha-gal antigen on bony union: a model of xenobone graft using GalT knockout mouse

Myung Ki Chung[1], Chin Youb Chung[1], Kyoung Min Lee[1], Soon-Sun Kwon[1], Sang Hyeong Lee[2], Dae Gyu Kwon[3], Ki Hyuk Sung[4], In Ho Choi[5], Tae-Joon Cho[5], Won Joon Yoo[5], Moon Seok Park[1]

**LEVEL-I/Basic science**

*Purpose*: Among the bone graft sources used currently, the availability of autografts is limited and allografts are expensive. Therefore, xenobone grafts have attracted attention as a new source of bone grafts, although immunologic rejection issues are unresolved. This study used a GalT knockout mouse model to investigate the effects of reducing the alpha-gal epitope using alpha-galactosidase on the union of porcine xenobone grafts.
*Methods*: Sixty-eight alpha-gal knockout C57/BL6 mice and eight wild-type mice were used. The mice were divided into five groups: In group 1 (26 alpha-gal knockout mice), an alpha-galactosidase-treated porcine xenograft was transplanted into the mouse femur to reduce antigenicity, and intramedullary fixation was performed. In group 2 (26 alpha-gal knockout mice), a non-treated porcine xenobone graft was performed. In group 3 (eight alpha-gal knockout C57/BL6 mice), syngenic bone grafts were performed. In group 4 (eight wild-type C57/BL6 mice), syngenic bone grafts were performed. In group 5 (eight C57/BL6 alpha-gal knockout mice), a bone defect model was obtained by maintaining the gap of the osteotomy site. Groups 3, 4, and 5 were used for positive and negative control groups. Qualitative immunohistochemical analysis of the porcine bone was performed to detect the presence of the alpha-gal epitope in groups 1 and 2. The concentration of the anti-alpha-gal antibody was evaluated using a quantitative ELISA at the time of sacrifice. Histologic and radiologic results (Goldberg method) for the bone union were compared.
*Results*: The qualitative immunohistochemical analysis showed that the alpha-gal epitope was reduced when xenobone grafts were treated with alpha-galactosidase. Compared with group 2, group 1 showed a low anti-alpha-gal antibody concentration in the ELISA results. In group 2, the anti-alpha-gal antibody concentration increased with time. Group 1 showed significantly better histologic union than group 2, but the amount of radiologic union was similar in the two groups.
*Conclusion*: Alpha-galactosidase treatment of a porcine xenobone graft can reduce the alpha-gal epitope. This reduction in the antigen could significantly reduce the humoral immune response to the alpha-gal antigen in C57/BL6 alpha-gal knockout mice, leading to significant improvements in histologic union. This study provides a relevant GalT knockout mouse model for detecting the effects of alpha-gal epitope reduction by alpha-galactosidase on the union of porcine xenobone grafts.
*Significance*: This study was performed to provide a GalT knockout mouse model to determine the effect of reduction in the alpha-gal epitope by alpha-galactosidase on the union of porcine xenobone grafts.

## EP97

### Determining the best treatment for simple bone cyst: a decision analysis

Sang Hyeong Lee[1], Chin Youb Chung[2], Kyoung Min Lee[2], Myung Ki Chung[2], Dae Gyu Kwon[3], Ki Hyuk Sung[4], Seung Yeol Lee[4], In Ho Choi[5], Tae-Joon Cho[5], Won Joon Yoo[5], Moon Seok Park[2]

[1]Dongguk University Hospital, Go Yang, Republic of Korea
[2]SNU Bundang Hospital, Seong Nam, Republic of Korea
[3]In-Ha University Hospital, In Cheon, Republic of Korea
[4]Myongji Hospital, Go Yang, Republic of Korea
[5]SNU Children's Hospital, Seoul, Republic of Korea

**LEVEL-I/Tumours**

*Purpose*: The treatment of simple bone cyst (SBC) in children varies significantly among physicians. This study examined which procedure is better for the treatment of SBC, using a decision analysis based on current published evidence.
*Methods*: A decision tree focused on five treatment modalities of SBC: observation, steroid injection, autologous bone marrow injection, decompression, and curettage with bone graft. Each treatment modality was further branched, according to the presence and severity of the complication. The probabilities of all cases were obtained by literature review. A roll back tool was utilized to determine the most preferred treatment modality. One-way sensitivity analysis was performed to determine the threshold value of the treatment modalities. Two-way sensitivity analysis was utilized to examine the joint impact of changes in probabilities of two parameters.
*Results*: The decision model favored autologous bone marrow injection. The expected value of autologous bone marrow injection was 0.9445, while that of observation, steroid injection, decompression, and curettage and bone graft was 0.9318, 0.9400, 0.9395, and 0.9342. One-way sensitivity analysis showed that autologous bone marrow injection was better than that of decompression, in the expected value when the rate of pathologic fracture or positive symptom of SBC after autologous bone marrow injection was lower than 20.4 %.
*Conclusion*: In our study, autologous bone marrow injection is the best choice as the treatment of SBC. However, the results were sensitive to the rate of pathologic fracture after the treatment of SBC. Physicians should consider the possibility of pathologic fracture when they determine the treatment method of SBC.
*Significance*: The significance of this study is to exam the best method for the treatment of SBC, using a decision analysis based on current published evidence.

## EP98

### Estimation of the recovery of physiological genu varum with linear mixed model

Myung Ki Chung[1], Chin Youb Chung[1], Kyoung Min Lee[1], Sang Hyeong Lee[2], Dae Gyu Kwon[3], Ki Hyuk Sung[4], In Ho Choi[5], Tae-Joon Cho[5], Won Joon Yoo[5], Moon Seok Park[1]

**LEVEL-III/Hip/Lower extremity**

*Purpose*: Although previous studies have provided reference values for the lower limb alignment in children with physiological genu varum, those often have methodological flaws, including problems with assumptions of statistical independence. In this study we intend to use appropriate statistical methods to determine reference values of the recovery of physiological genu varum by using a linear mixed model (LMM).
*Methods*: The database of our institution was searched and teleroentgenograms of patients up to 5 years of age with clinical diagnosis of physiological genu varum were selected. The mechanical tibio-femoral angle (mTFA), mechanical lateral distal femoral angle (mLDFA), and mechanical medial proximal tibial angle (mMPTA) were measured from each radiograph. These measures were then incorporated in LMM. The reference values for mTFA, mLDFA, and mMPTA were calculated by LMMs with age, sex, and side of the limbs, as the fixed effects with each subject as random effects.
*Results*: A total of 425 teleroentgenograms were evaluated from 161 patients with physiological genu varum. Age, sex, and limb side terms were found to be significant contributing factors to the measures. In the LMM, with the age as quadratic, mTFA was equal to −0.3-degree varus [95 % confidence interval (CI) −5.9° to 5.3°] at the age of 3 years. The mLDFA was equal to 87.8° (95 % CI 82.8°–92.8°) at the age of 4.5 years. The mMPTA was equal to 88.8° (95 % CI 80.4°–97.2°) at the age of 2.5 years.
*Conclusion*: An LMM fitted well to estimate the natural recovery of physiological genu varum. Age, sex, and side of the limbs were found to be significant factors in the estimation of mTFA, and age and side of limbs were significant in estimation of mLDFA and mMPTA.
*Significance*: The significance of this study was to use appropriate statistical methods to determine reference values of the recovery of physiological genu varum by using a LMM.

## EP99

### Annual changes in radiographic indices of the spine in cerebral palsy patients

Byung Chae Jo[1], Chin Youb Chung[1], Kyoung Min Lee[1], Sang Hyeong Lee[2], Dae Gyu Kwon[3], Ki Hyuk Sung[4], Seung Yeol Lee[4], In Ho Choi[5], Tae-Joon Cho[5], Won Joon Yoo[5], Moon Seok Park[1]

**LEVEL-III/Cerebral Palsy**

*Purpose*: We estimated annual changes in radiographic indices of the spine in cerebral palsy (CP) patients and analyzed factors influencing its progression rate.

*Methods*: We included CP patients who had taken whole spine radiographs more than twice and were followed for at least 1 year. Scoliosis Cobb angle, coronal balance, apical vertebral translation, apical rotation, and pelvic obliquity were measured on anteroposterior (AP) radiographs; thoracic kyphosis and lumbar lordosis angles and sagittal balance on lateral radiographs; and migration percentage on AP hip radiographs to determine hip instability. For each Gross Motor Function Classification System (GMFCS) level, the Cobb angles, apical vertebral translation, coronal and sagittal balance, and pelvic obliquity were adjusted by multiple factors with a linear mixed model.
*Results*: A total of 184 patients (774 radiographs) were included in this study. There was no significant annual change in scoliosis Cobb, thoracic kyphosis, and lumbar lordosis angles in the GMFCS level I–II and III group. Whereas, in the GMFCS level IV–V group, annual increase of the scoliosis Cobb angle was 3.4° ($p = 0.020$). The thoracic kyphosis angle increased by 2.2° ($p = 0.018$) annually in the GMFCS level IV–V group. Apical vertebral translation increased by 5.4 mm ($p = 0.029$) annually in the GMFCS level IV–V group. Progression of coronal and sagittal balance and pelvic obliquity with aging were not statistically significant. Sex, hip instability, hip surgery, and triradiate cartilage did not affect the progression of scoliosis and the balance of the spine and pelvis.
*Conclusion*: The scoliosis Cobb angle, thoracic kyphosis angle, and apical vertebral translation in the GMFCS level IV–V of CP patients progressed with age. These findings can predict radiographic progression of scoliosis in CP patients.
*Significance*: The significance of this study is to estimate the annual changes of radiographic indices of the spine in CP patients and to analyze the factors that influence the rate of progression using a LMM application.

## EP100

## Factors determining outcome in the Ponseti management of idiopathic structural clubfoot

Sally Tennant[1]

[1]Rnoh, London, UK

**LEVEL-I/Club-foot**

A prospective analysis of idiopathic clubfeet presenting to a single orthopaedic surgeon over a period of 10 years was undertaken. 140 feet in 96 babies underwent the Ponseti technique consisting of weekly manipulations and castings, followed by tenotomy under local anaesthetic and the wearing of a foot abduction brace (FAB) for 5 years.

Demographic data was collected including birth history and family history. The age at which casting began, number of casts required, and the need for TA tenotomy were recorded. Outcome measures included the need for further casting, second tenotomy, tibialis anterior tendon transfer, and Roye score.

Results

Overall, the median number of casts required before tenotomy was 5 (range 3–13), and the tenotomy rate was 76 %. In babies presenting after 10 weeks of age, fewer casts were required, the tenotomy rate was lower, and they were more likely to have unilateral feet than babies presenting early for treatment (<5 weeks of age).

In those with a positive family history, slightly more casts were required and the tenotomy rate was slightly higher at 83 %. Bilateral feet had a higher median number of casts and a higher tenotomy rate than unilaterals (81 vs. 73 %).

75 % of feet had an excellent result with a corrected foot with no further casting or surgery. Repeat casting for relapse was required in 15 patients. Further surgery was required in 10 patients including 3 Tibialis Anterior tendon transfers, (2 awaiting), 5 repeat tenotomies, 1 plantar fascia release, and 1 postero-medial release. 5 babies were lost to follow-up.

Unilateral feet were less likely to require further casting or surgery (7 %) compared with bilateral feet (24 %). Antenatal counselling had been undertaken in two-thirds of mothers and could be related to compliance in the FAB, and outcome.

7 feet were classified as atypical, requiring more casts and being more difficult to fit into boots and bars. None of these underwent further surgery other than a repeat TA tenotomy. They have excellent compliance and very good results.
*Conclusions*: Late presenting Clubfeet and atypical feet can have an excellent outcome using the Ponseti method. Antenatal counselling has a positive effect on compliance and outcome. Bilateral clubfeet and those with a positive family history in this cohort required more casts, had a higher tenotomy rate and were more likely to experience a relapse.

## EP101

## Is early fever following pelvic and/or femoral osteotomy in children a predictor of post-operative infection?

Shahrier Sarker[1], Andreas Rehm[1], Francis Sim[1]

[1]Addenbrooke's Hospital, Cambridge, UK

**LEVEL-IV/Musculoskeletal infections**

*Introduction*: Fever is a systemic response to surgery in children. Postoperative fever is common, causing anxiety amongst patients, parents and doctors, often leading to further investigations for infection. The aim of this study was to determine the incidence of fever after such surgery and determine any links to infection.
*Methods*: 112 operations performed by one surgeon on children <16 years were identified. Patient demographics, maximum daily post-operative temperature, anaesthetic time, intra operative blood loss, blood transfusion, complications and any microbiological investigations were recorded. Student's t-test was used for statistical analysis.
*Results*: 27 % of children had a temperature of ≥37.5 °C immediately after surgery with 62.5 % on day 1 post-op. The maximum temperature was 38.5 °C. The mean temperature and standard deviation of the days after surgery were: Day 0: 37.12 ± 0.65 °C, Day 1:37.58 ± 0.44 °C, Day 2: 37.66 ± 0.66 °C, Day 3: 37.54 ± 0.48 °C, Day 4: 37.63 ± 0.45 °C and Day 5: 37.42 ± 0.48 °C. The temperature was significantly higher ($p < 0.05$) in each of first 4 post op days. There was no significant difference in the mean temperatures of days 1–4. There was no significant difference in the mean temperature of patients (1) having an intraoperative blood transfusion, (2) between those aged <2 years and those >2 years and (3) between patients weighing <15 kg and those >15 kg. The mean temperature of patients undergoing surgery longer than 240 min was significantly higher that those having shorter surgery ($p < 0.05$). Of these children only three had a microbiologically proven infections requiring further treatment.
Our results reveal that fever is a common finding after femoral and pelvic osteotomy in children, especially in prolonged surgery. Most patients have a temperature >37.5 °C from the first postoperative day. It does not predict infection and should not be aggressively investigated in an otherwise well child.

## EP102

### Conception of achillotomy in children with arthrogryposis

Alexey Baindurashvili[1], Svetlana Trofimova[1], Denis Derevianko[2], Dmitriy Bucklaev[2]

[1]Turner's Institute, St. Petersburg, Pushkin, Russian Federation; [2]Turner Institute, St. Petersburg, Pushkin, Russian Federation

**LEVEL-III/Club-foot**

*Introduction*: Achilles tenotomy is the last step in clubfoot correction by Ponseti method, but severe soft tissue rigidity in patients with arthrogryposis make often us to reconsider the conception of achillotomy.
Aim of our study was to analyze effectiveness of achillotomy in children of the first year of life with arthrogryposis.
*Materials and Methods*: 26 patients with AMC (52 feet) and 4 children (7 feet) with distal type, treated by Ponseti method were selected for the study. Mean number of casts in AMC group was 7.8 ± 0.21 (from 6 to 12) and in distal type group 4.7 ± 0.6 (from 3 to 7).

During conservative treatment achillotomy was performed in 25 feet of AMC group and 7 feet in distal type. Other children with AMC had residual deformity and required surgery.
*Results*: Analyze of effectiveness of the procedure, performed for equinus to be eliminated revealed that mean angle of calcaneus position change in AMC group was 23° ± 2°. Mean angle of equines before treatment in this children with AMC was 40° ± 1.4° (from 30° to 70°) and after achillotomy 15° ± 1.4° (from −5° to 25°).
Mean angle of calcaneus position change in distal type was 34° ± 1.5°. Mean angle of equines before treatment in this children with distal type was 27° ± 2° (from 15° to 45°) and after achillotomy −7° ± 3°, i.e. 7° of dorsiflexion (from −15° to 0°).

Residual equinus after achillotomy, not excideeng 20° eliminated in all patients with further weekly casting and correction of hill position during 3–4 weeks.

Thus mean angle of correction was 40° (23° after achillotomy and 20° after further casting).
*Conclusion*: We come to conclusion that effectiveness of achilolotomy in order equinus to be eliminated depends on type of the pathology (it is the most effective in children with distal type). If equinus deformity exceeds 40° achillotomy is not helpfull. Such patients should be considered for surgery.
*Significance*: Consideration of angle of equines before treatment help to choose the highly effective management of clubfoot in children of the first year of life with arthrogryposis.

## EP103

### Development of an immature live porcine hip arthroscopy model for training and for evaluating the acute effects of prolonged traction on the femoral head

Manoj Ramachandran[1], Martin Gottliebsen[2], Ahmed Abood[2], Juan Manuel Medina[2], Ole Rahbek[2], Bjarne Moller-Madsen[2]

[1]Royal London Hospital, London, UK
[2]Aarhus University, Aarhus, Denmark

**LEVEL-III/Basic science**

*Purpose*: Hip arthroscopy is being used increasingly for diagnosis and treatment of paediatric and adolescent hip disorders. To date, only one previous animal model has been reported, used specifically to test the healing of arthroscopic repaired labral tears in skeletally mature sheep (Phillipon et al. 2007). There have been no previous animal models developed for the immature hip, as training adjuncts or to assess the acute effects of hip arthroscopic traction on the femoral head.
*Methods*: We developed a specific pig apparatus for traction (PAT) based on standard hip arthroscopy traction apparatus for obtaining and maintain hip joint distraction in live anaesthetised immature Danish domestic pigs (average weight 15 kg). The PAT was successfully used in 10 consecutive pigs to maintain sufficient joint distraction to allow insertion of a 2.7 mm cannula from anterolateral and anterior portals without damaging the femoral head. The acute effects of traction were then assessed by maintaining joint distraction in one hip joint for 0 min (n = 3), 1 h (n = 3) and 2 h (n = 3) compared to an acute avascular necrosis model on the contralateral hip performed by an open procedure. The pigs were transferred anaesthetised to a 3T MR scanner and images were obtained pre- and post-gadolinium enhancement to assess acute femoral head perfusion. The experiment complied with the Danish Law on Animal Experiments and was approved by the Danish Ministry of Justice.
*Results*: The live porcine hip arthroscopy model was used successfully to obtain and maintain traction to act as an adjunct to training in hip arthroscopic surgery. In the traction experiments, femoral head blood flow was maintained in all hips on traction at all time points following release of traction, showing full acute recovery from prolonged traction. In comparison, acute complete loss of perfusion to the contralateral femoral head (open avascular necrosis model) was confirmed in all pigs on MR.
*Conclusions*: In the first live animal model for paediatric hip arthroscopy, we have shown that traction can be consistently achieved for training purposes and that the flow to the femoral head is maintained, even after prolonged traction. Clinically, prolonged traction for hip arthroscopy is likely to be limited by other factors such as soft tissue complications e.g. neurapraxia.

## EP104

### External fixation or elastic intramedullary nailing for the treatment of femoral fractures in children?

Emanouil Morakis[1], Dimitrios Petratos[2], Dimitrios Katsoulas[1], Panagiotis Krallis[1], John Anastasopoulos[1]

[1]Aghia Sophia Hospital, Athens, Greece
[2]Mitera Hospital, Athens, Greece

**LEVEL-III/Trauma/Lower extremity**

*Purpose*: To evaluate retrospectively and compare the treatment outcomes of femoral fractures in children 6–14 years old, using flexible intramedullary nailing (FIN) with those of external fixation (EF).
*Methods*: 58 closed femoral fractures in 56 children were surgically treated in our hospital between 2000 and 2009; 34 fractures using FIN and 24 using EF. The treatment outcomes were compared in terms of the length of hospital stay, time to partial (PWB) and full weight bearing (FWB) and time to fracture union. The immediate postoperative and final alignment at the fracture site was measured. The complication rates at the final review were also recorded. Statistical analysis was performed using SPSS 19 software.
*Results*: Mean follow-up was 36 months for the FIN group and 34 months for the EF group. The two groups of patients had similar age ($p = 0.232$) and weight ($p = 0.108$). The time to PWB ($p = 0.02$) and FWB ($p = 0.012$) as well as the time to fracture union ($p = 0.001$) were all significantly shorter in the FIN group. Regarding all the other parameters that were recorded, the results were better in the FIN but the differences between the two groups were not statistically significant. Although the total number of complications was similar ($p = 0.342$) in the two groups, major complications—two refractures—occurred only in the EF group.
*Conclusion*: FIN leads to better outcomes than external fixation for most femoral fractures in children aged 6–14. EF should be reserved only for highly comminuted femur fractures.
*Significance*: Level of evidence III.

## EP105

### Screening program for neonates at risk for DDH: comparing first radiological evaluation at 5 months with the standard 12 weeks ultrasound: a prospective cross-sectional cohort study

Dorien Geertsema[1], Joris E Meinardi[1], Marta Fiocco[1], Michiel A. J. Van De Sande[1]

[1]LUMC, Leiden, The Netherlands

**LEVEL-II/DDH**

*Purpose*: Recent trials on the treatment of mild DDH (Graf 2b/c) have suggested a watchful waiting policy, postponing treatment, allowing for auto-correction of immature hips. In order to assess radiological outcome of postponing diagnosis and treatment we compared two screening protocols for DDH; early 12 weeks ultrasound (group 1) with X-ray at 5 months (group 2), both including ultrasound evaluation for hip dislocation at 6 weeks with abnormal findings after physical evaluation.
*Methods*: From our prospective database (2009–2013), we analysed 3,536 screened neonates (1,745 males (49.3 %) and 1,791 females (50.7 %)) at an age of 2–3 weeks. In the presence of risk-factors for DDH, 460 screened infants were assigned to an X-ray at 5 months between 2009–2010 and between 2011–2013, 651 infants were assigned to an ultrasound at 3 months. In case of DDH, appropriate treatment was started and radiological follow up occurred at 8, 10, 12 and 18 months with measurement of the Acetabular Index (AI) according to Tönnis. We compared the incidence and severity of persistent DDH at 18 months. Risk analysis was performed using linear-regression (OR).
*Results*: Both groups were comparable for risk-factors such as gender, breech and family history. After screening both groups, 98 patients (2.8 %) had DDH; n = 37 in group 1 and n = 61 in group 2. At 18 months follow-up, 7 patients showed persistent DDH; n = 3 (8.1 %: 3/37) in group 1 and n = 4 (6.6 %: 4/61) in group 2. The mean AI at 18 months for the (left hip) for group 1 was: 22.4° (95 % CI 20.6°–24.3°) versus group 2: 22.3° (95 % CI 21.2°–23.4°), $p = 0.098$. The mean AI (right hip) for group 1 was: 21.9° (95 % CI 18.9°–24.9°) versus 21.2° (95 % CI 20.5°–22.0°) in group 2: $p = 0.293$. We found no difference in incidence of persistent DDH between both groups (policy after screening: ultrasound at 3 months or X-ray at 5 months) after 18 months adjusted for gender, breech, family history: OR 0.519 (0.07–3.845).
*Conclusion*: Equal amounts of neonates were screened at 3 and 5 months and eventually diagnosed with DDH. During follow-up, both groups showed comparable numbers of persistent DDH with comparable normalisation of the AI. We can therefore conclude, that it is save to screen neonates with risk-factors by ultrasound at around 5 months. This prevents overtreatment and allows for comparable radiological results. Screening for suspected hip dislocation remains to be performed at an earlier stage.

## EP106

### Talonavicular arthrodesis and tibialis posterior advancement for pesplanovalgus in children with cerebral palsy

Nuri Menekseoglu[1], Cemil Yildiz[1], Kenan Koca[1], Serkan Akpancar[1]

[1]GATA, Ankara, Turkey

**LEVEL-IV/Cerebral Palsy**

*Objective*: The aim of the study was to investigate results of talonavicular arthrodesis and tibialis posterior advancement for pesplanovalgus in children with cerebral palsy with clinical and radiographic parameters.
*Methods*: Ten feet underwent talonavicular arthrodesis and tibialis posterior advancement in seven patients with cerebral palsy between January 2010 and April 2013. Three patients had bilateral same surgery. Achilles tendons were lengthened at same time in four patients. Five of patients were male and two of patients were female. Mean age was 15.1 ± 2.07 (range 12–18). All patients were diplegic ambulatory cerebral palsy. Patients were evaluated with Yoo Clinic Outcome Score Scale (YCOSS), Functional Mobility Scale (FMS) and radiographic measurements preoperatively and postoperatively. Angles of calcaneal pitch, talocalcaneal and talus-first metatars were measured on lateral foot radiography in stand position. Angles of talonavicular overlap, naviculocuboid overlap and talus-first metatarsus were measured. In addition, lengthens of medial and lateral colons were measured. Short leg casting applied for 6 weeks after surgery. Preoperative and postoperative parameters were compared statically with SPSS v15.0.
*Results*: Foot pain was reduced in all patients andalf of the patients did not need foot orthotic device after surgery. Six patients had good results according to the YCOSS. Pain was eliminated completely in six foot and reduced in three foot. Pain continued in only one foot.
FMS was improved in all patients statically. It was increased from 3.9 ± 1.37 to 4.9 ± 1.37 in 5 m; 2.6 ± 1.34 to 4.5 ± 1.43 in 50 m; 2.1 ± 0.99 to 4.4 ± 1.34.

Radiographic measurements were improved in all patients. Talocalcaneal angle was reduced from 46.5 ± 11.8 to 29.9 ± 8.37 ($p < 0.05$). Calcaneal pitch angle was increased from 10.2 ± 8.33 to 12.2 ± 7.14 ($p > 0.05$). Talonavicular overlap and calcaneocuboid overlap were decreased significant statistically. Talus-first metatarsal angle was decreased in both AP and lateral radiographies. Medial colon of foot was decreased, but it was insignificant statistically.
*Conclusion*: The current study showed that talonavicular arthrodesis and tibialis posterior tendon advancement surgery was effective method by improving Yoo Clinic Outcome Score Scale and Function Mobility Scale. This surgery also improved radiographic measurement included talocalcaneal angle, talus-first metatarsals angles, talonavicular overlap and naviculocuboid overlap.

## EP107

### Management of the tibia in osteogenesis imperfecta congenita (OIC) with special emphasis on the choice of implant, surveillance and implant related complications

Matthias Rogalski[1], Katharina Fliesenberg[1], Mario Deja[1], Karen Burgemeister[1], Joseph Zacher[1], Christian Arnold[1]

[1]Helios-Klinikum, Berlin Buch, Berlin, Germany

**LEVEL-III/Metabolic**

*Purpose*: Telescopic rodding, especially after introduction of the Fassier–Duval system and sliding technique with ESIN, is the preferred method for femoral correction and stabilization in growing OIC-patients. The surgical treatment of tibial deformity due to this weak bone disease is an ongoing challenge and associated with a high number of complications. The question of the preferred tibial implant in children with OIC has not yet been answered. We present a critical review and evaluation of our experience of tibial management in OIC with special emphasis to complications and implant surveillance.
*Methods*: We reviewed the results of different methods of intramedullary fixation devices after tibial fracture treatment and correction osteototomy in 33 OIC patients over a time period of 20 years. The surgical techniques included intramedullary stabilization with non elongating single rush rods (n = 21), sliding dual ESIN (n = 8), Bailey-Dubow rodding (n = 12) and the Fassier–Duval system (n = 24). We analyzed fracture rate after treatment, nail and disease related complications and survival rate of each rod.
*Results*: The median age at surgery for the whole population was 5 years (2.5–14). The survival rates (median interval between pimary surgery and rod revision) were the following: single rush rod: 30 months, sliding dual ESIN: 28 months, Bayley–Dubow-telescopic rod: 30 months and Fassier–Duval system: 38 months. Complication rate, functional outcome and radiological data were obtained. The median follow up was 10 years (3–20 years).
*Conclusions*: Rush rods, dual ESIN and BD-telescopic nails are equally effective in terms of surveillance and complication rates in growing OIC patients. Up to now there seems to be a tendency for lower complication rates with the Fassier duval system. Refracture, recurrent deformity (antecurvation and bending) and nail migration are still problems and limiting the surveillance rate of different intramedullary devices for the tibial bone in OIC-Patients. Therefore we modified some steps of the surgical procedure and recommendations for postoperative management. Nevertheless some disease related problems still remain and demand an individualized management.

## EP108

### Risk factors for long-term sequelae of bone and joint infections in children: a multicentre study

Marta Salom[1], Ignacio Jr Sanpera[2], Julio Duart[3], Serafín García-Mata[3], Guillem Frontera-Juan[2]

[1]Hospital Universitario La Fe, Valencia, Spain;
[2]Hospital U Son Espases, Palma de Mallorca, Spain;
[3]Hospital de Navarra, Pamplona, Spain

**LEVEL-IV/Musculoskeletal infections**

*Introduction*: Bone and joint infections in children can cause long-term sequelae. Rates published range between 0 and 66 %, with risk factors not clearly defined.
*Objectives*: To evaluate the rate of long-term sequelae of bone and joint infections in three different hospitals analysing the risk factors involved.
*Methods*: During the period of 2008–2012 the cases presenting with bone and joint infections in three referral hospitals were studied. The patients were divided into three large groups according to the type of infection: Acute Osteomyelitis (AOM), Septic Arthritis (SA) and Subacute Osteomyelitis (SAOM). The following parameters were recorded: age, sex, bone or joint affected, delay in diagnosis, fever and acute reactants on admission, positive culture in joint aspirate or blood, surgical treatment, length of antibiotic regimen and presence or absence of radiological and clinical sequelae.
*Results*: A total of 130 patients were analysed; the patients were divided into 48 SA, 36 AOM and 46 SAOM. No significant differences in ages were found between groups. Nine patients developed long-term sequelae, 2 SA, 3 AOM and 4 SAOM. The joint affected was the hip in the two cases of SA. The bone affected was the proximal femur in the three cases of AOM, a hand phalanx in one case of SAOM and the distal femur in the other three cases of SAOM. Comparing the cases with and without sequelae in the different groups, a significant difference in the delay of diagnosis was found, being longer in the cases with sequelae. No significant differences were found in the rate of surgical treatment or isolated organism. Patients with sequelae had higher rates of positive cultures as reported in the literature. Needless to say, in the cases with sequelae, antibiotic treatment and follow up were longer.
*Conclusions*: Although the treatment of bone and joint infections in developed countries has improved, there are still long-term sequelae. We have performed a multicentre study to investigate the rates of sequelae and to identify the risk factors to develop them.

## EP109

### Is intramuscular psoas lengthening during single-event multilevel surgery effective in diplegic spastic children with cerebral palsy?

Cindy Mallet[1], Brice Ilharreborde[1], Anne-Laure Simon[1], Ana Presedo[1], Keyvan Mazda[1], Georges Penneçot[1]

[1]Robert Debré Hospital, Paris, France

**LEVEL-III/Cerebral Palsy**

*Purpose*: In spastic diplegic ambulatory children, psoas contracture leads to lack of adequate hip extension during mid and final stance that impedes forward progression. Intramuscular psoas lengthening (IMPL) to improve hip extension during stance remains controversial in literature. The aim of this study was to determine efficacy of IMPL during single-event multilevel surgery (SEMLS) in ambulatory spastic diplegic children.
*Methods*: Spastic diplegic ambulatory children (GMFCS 2–3) that underwent SEMLS for clinical hip flexion contracture >10° and lack of hip extension >10° at final stance (at 50 % of the gait cycle), were retrospectively reviewed. Two groups were defined: one group had psoas lengthening surgery (psoas group) and one group did not (-control group). Clinical and kinematic data including clinical hip extension, hip extension at terminal stance, Gillette Gait Index (GGI), walking velocity and stride length were analyzed preoperatively and with at least 1 year follow-up. Changes in each data pre and post-operatively were compared in the two groups.
*Results*: Forty-seven lower limbs (32 in control group and 15 in psoas group) in 34 patients were analyzed. The two groups were statistically comparable regarding age of surgery, clinical and kinematic hip flexion, GGI, stride length and walking speed. Both groups showed significant improvement in postoperative hip extension at terminal stance and GGI. Clinical hip extension, walking velocity and stride length increased significantly only in psoas group. However, postoperative improvement in hip extension at terminal stance, in walking velocity and stride length was not significantly different in the two groups.
*Discussion*: IMPL did not enhance the improvement in hip extension at terminal stance. This was more likely linked to posterior displacement of the ground reaction force vector at the hip, caused by improvement of position of knee and ankle due to SEMLS.
*Conclusion*: IMPL should be indicated in case of hip flexion contracture >10° in whom SEMLS would surely correct knee and ankle position.

## EP110

### Effect of femoral head ossific nucleus in treatment of hip dislocation: systematic review and meta-analysis update 2014

Rafal Niziol[1], Evangelia Protopapa[2], Andreas Roposch[1], Kuldeep Stohr[1]

[1]Great Ormond Street Hospital, London, UK
[2]UCL Institute of Child Health, London, UK

**LEVEL-II/DDH**

*Purpose*: Despite multiple studies, the controversy of whether a present ossific nucleus protects against osteonecrosis at the time of reduction of a developmentally dysplastic hip still remains. The aim of this study is to investigate the relationship between the presence of the femoral head ossific nucleus at the time of hip reduction and the development of osteonecrosis following treatment of a dislocated hip in DDH.
*Methods*: We systematically searched the literature up until 2014 for studies reporting on hip dislocation in DDH, ossific nucleus and osteonecrosis. Two reviewers independently assessed abstracts and quality of studies using the 'Grading of Recommendations Assessment, Development and Evaluation' statement. Studies reporting adequate data were included in a meta-analysis with the main outcome defined as the development of osteonecrosis of grade I–IV seen at a minimum of 2 years after reduction.
*Results*: We identified 14 studies, of which 5 studies (36 %) reported a protective effect of the ossific nucleus against the development of osteonecrosis. The remaining studies did not find an effect. The quality of evidence was similar among studies reporting protective and non-protective effects. A meta-analysis of 11 studies (1,015 patients) showed no significant relationship between ossific nucleus and osteonecrosis (RR 0.88; 95 % CI 0.56–1.41). When only radiographic changes of grades II–IV were defined as osteonecrosis, the results remained insignificant (RR 0.67; 0.41–1.08). In 253 cases of closed reduction, the ossific nucleus had a protective effect (RR 0.50; 0.26–0.94) against the development of osteonecrosis grades II–IV (mean follow up, 6.12 years, range 2–16 years).
*Conclusion*: We found no protective effect of the femoral head ossific nucleus on the development of osteonecrosis following reduction of a dislocated hip, regardless of the severity of osteonecrosis. However, a protective effect was seen in hips reduced by closed means. The quality of evidence on this topic is probably strong enough to have confidence in the effect estimates of this meta-analysis and it is unlikely that future studies will change it.

## EP111

### Are the validity and reliability of children's hip parameters affected by pelvic axial positioning during X-ray acquisition?

Ayman Assi[1], Ziad Bakouny[1], Aren Joe Bezdikian[1], Fares Yared[1], Christophe Sauret[2], Nour Khalil[1], Wafa Skalli[2], Ismat Ghanem[3]

[1]School of Medicine USJ, Beirut, Lebanon
[2]LBM ParisTech, Paris, France
[3]Hotel-Dieu de France Hospital, Beirut, Lebanon

**LEVEL-III/Hip/Lower extremity**

*Introduction*: In order to assess hip disorders in children, clinical parameters are measured on frontal X-rays of the pelvis. These radiographs require standard positioning of the patient during acquisition. The aim of this study was to estimate the effect of erroneous positioning of children, during pelvis frontal X-ray acquisition, on the reliability and validity of hip parameters.
*Methods*: Helical pelvis CT scans (slice thickness: 0.6 mm) of 8 children (3 F and 5 M, age: mean 12—SD 2.2) were considered. Frontal Digitally Reconstructed Radiographs (FDRRs) were reconstructed from CTs. Then, for each patient, axial rotation (AR) of the pelvis was simulated and the corresponding FDRRs were reconstructed at 5°, 10°, 15° and 20° of axial rotation. Clinical parameters were measured digitally on each radiograph, for both the left and right sides of each patient: Vertical Center Edge (VCE) angle, HTE angle, Sharp's angle, Lateral Subluxation (LatSub) angle, Acetabular Fossa relative to the ilioischial line (AcetFossa), Acetabular Depth (AcetD) distance, Acetabular Width (AcetW) distance and Migration Percentage of the Femoral Head (MPFH). Three trained operators repeated the measurements 3 times each, in each (AR) position. Intraclass Correlation Coefficient (ICC) was evaluated for intra and inter-observer agreement. The 95 % confidence interval (95 %CI) was calculated as 2SDs of inter-observer reliability. The bias of each clinical parameter, in each AR position, was calculated as the absolute mean difference relatively to the 0° position.
*Results*: Intra and inter-observer agreement was shown to be very high (ICC > 0.9) for all parameters and all AR positions. In the absence of AR of the pelvises (0°), the 95 % CI of HTE, VCE, Sharp's angle and MPFH were lower than 5 measurement units (m.u.)and lower than 1 cm for the AcetabD, AcetW, AcetFossa and LatSub. The 95 % CI increased with pelvic AR: it exceeded 5 m.u. for VCE, Sharp's angle, MPFH and reached 7° for HTE at 20° of AR. However, it remained constant for the remaining parameters. All the parameters showed an increase in bias during AR of the pelvis, where MPFH and VCE showed greatest bias (7.5 % and 6° respectively) at 20° position. AcetabD, AcetW, AcetFossa and LatSub exhibited a bias <1 cm.
*Discussion*: Hip parameters measured on frontal radiographs were shown to be less accurate and less reliable when pelvic AR increased.

Bias exceeded 10 % of normative values for most of the clinical parameters when AR exceeded 10°. This bias could significantly affect hip disorders' assessment.

## EP112

### The etiology of internal hip rotation during gait in children with cerebral palsy: a 3D subject-specific musculoskeletal analysis

Abir Massaad[1], Ayman Assi[2], Ziad Bakouny[2], Nour Khalil[2], Christophe Sauret[3], Wafa Skalli[3], Ismat Ghanem[4]

[1]Gait Lab SESOBEL, Beirut, Lebanon
[2]School of Medicine USJ, Beirut, Lebanon
[3]LBM ParisTech, Paris, France
[4]Hotel-Dieu de France Hospital, Beirut, Lebanon

**LEVEL-II/Cerebral Palsy**

*Introduction*: Internal hip rotation (IHR) during gait is widely encountered in children with Cerebral Palsy (CP). The involvement of femoral anteversion, medial hamstrings, adductors and gracilis in this abnormal gait behavior has been previously studied using either generic models or physical examination [1, 2]. The aim of this study is to assess the causes behind IHR in children with CP based on 3D subject-specific musculoskeletal parameters.
*Materials and Methods*: Twenty-two CP children (spastic diplegia N = 16, spastic hemiplegia N = 6, mean age 11.6 ± 3), with neither medical nor surgical history had undergone 3D gait analysis (3DGA). The following kinematic parameters were used to evaluate IHR: hip rotation at initial contact (P1), peak hip rotation during stance phase (2), mean hip rotation during stance phase (P3). The patients underwent an EOS® biplanar X-ray exam in order to calculate the femoral anteversion (FA), acetabular parameters (ante/retro version: rotation around the vertical axis of the pelvis, abd/adduction: rotation around the anterio-posterior axis of the pelvis) and sagittal pelvic parameters (pelvic incidence, sacral slope and pelvic tilt) in 3D. Axial MRI acquisitions were performed in order to calculate 18 belly muscle lengths in each lower limb. Musculoskeletal parameters were matched to those of 9 typically developing (TD) children. Pearson and Spearman correlations tests were applied on muscle lengths, 3D skeletal parameters and kinematics.
*Results*: Fifteen lower limbs presented excessive IHR compared to normative values. For these 15 lower limbs, FA was significantly increased in children with CP ($p < 0.001$), when compared to TD children, and was correlated to P3 (R = 0.54). 3D acetabular anteversion and abduction were correlated to P1 (R = 0.64) and P2 (R = −0.5) respectively. Pelvic incidence was correlated to P3 (R = −0.63). Muscle lengths of the gracilis, adductor brevis and longus were significantly reduced in CP compared to TD children ($p < 0.001$) but were not correlated to hip kinematics. However, adductor magnus length was correlated to P2 (R = −0.86).
*Discussion*: These preliminary results showed that 3D FA might contribute to IHR during gait, which is not in concordance with previous studies based on generic models [1, 3]. Moreover, 3D acetabular parameters, studied for the first time in CP, were shown to be related to IHR during gait. More studies should further explore the potential effect of acetabular and pelvic parameters on gait profiles.

## EP113

### The comparison of the results of treatment with Tubingen abduction orthosis in graf type 2C and Type D developmental hip dysplasia patients

Mithat Oner[1], Ibrahim Karaman[1], Ibrahim Halil Kafadar[1], Ahmet Guney[1]

[1]Erciyes University, Kayseri, Turkey

**LEVEL-IV/DDH**

*Objective*: There is no consensus on the optimal treatment device for unstable hips in the infants. The aim of this study was to evaluate the treatment effect of the Tübingen hip flexion splint in Graf type 2C hips and Type D hips.
*Materials and Methods*: This prospective study was performed between April 2011 and September 2013. We evaluated 51 patients (82 hips) during this period. 26 hips were diagnosed as type 2C (20 female, 6 male) and 56 hips were classified as type D (48 female, eight male). All this infants underwent Tübingen orthotic treatment. Treatment was terminated when development into a Graf type 1 mature hip on ultrasonography was detected.
*Results*: Type 2C Group: The mean age at the beginning of the treatment was 13 weeks (8–22). The mean total treatment time was 14 weeks (12–24). The mean follow-up period of patients was 21 months. When we evaluate the results, we found a 100 % success rate. Type 1 mature hips were obtained in all hips in this group. None of the patients required hip spica cast application or surgical intervention. None of the patients in this group had any complications.
Type D Group: The mean age at the beginning of the treatment was 12 weeks (8–19). The mean total treatment time was 18 weeks (12–26). The mean follow-up period of patients was 22 months. Treatment was successful in 52/56 hips (92.8 %). Hip spica application under general anesthesia was required for 4 hips (3 patients). None of the patients in this group had any complication. No statistically significant difference was found between the two groups when evaluated the mean age of beginning treatment and the mean total treatment time. There was no statistically significant difference between the two groups when treatment success rates were compared.
*Conclusion*: The Tübingen hip flexion Orthosis provides a reliable fixation of infants hips. Tübingen orthosis application is highly effective in the treatment of both groups (Type 2C and Type D). Also patients who has a late diagnosis (12–20 weeks of life) can be successfully treated with the Tübingen hip flexion Orthosis. Especially the development of type D hips into mature type may be delayed. So this group should be monitored more carefully.

## EP114

### Eight plate epiphyseodesis in children with sick physes

Matea Pirsa[1], Ozren Kubat[2], Igor Smigovec[2], Tomislav Dapic[2], Darko Anticevic[2]

[1]University of Zagreb Medical, Zagreb, Croatia
[2]Clinical Hospital Centre Zagreb, Zagreb, Croatia

**LEVEL-IV/Hip/Lower extremity**

*Aim*: Sick physes is the common denominator for a number of congenital syndromes, dysplasias or acquired diseases which damage the growth plate. Children affected by these disorders tend to develop angular limb deformities of varying extent early in life. Techniques of hemi-epiphyseodesis have been introduced into the orthopedic arsenal to manipulate the physis into a favourable position to achieve proper limb alignment at the end of growth. Our objective was to assess the achieved correction and report on complications and limits in treatment of children with sick physes using eight plates.
*Materials and Methods*: We reviewed charts and radiographs of children with sick physes treated by hemiepiphyseodesis according to Stevens around the knees and/or ankles in our Department from May 2007 to June 2013. Measurements of the mechanical axis deviation (MAD), mechanical lateral distal femoral angle (mLDFA), medial proximal tibial angle (MPTA) and joint line congruency angle (JLCA), as well as lateral distal tibial angle (LDTA) for those with ankle varus/valgus, were performed on preoperative and last follow-up full-length weightbearing radiographs. Time to correction, achievement of correction and complications were noted.
*Results*: 25 children (17 males, 8 females) with 38 knees (45 plates) and 7 children (4 males, 3 females) with 10 ankles (10 plates) with coronal plane deformity were identified. In the knee group, preoperative mean values were; MAD 32.7 mm (range 5–75 mm), mLDFA 91.4° (range 76°–131°), MPTA 89° (range 75°–116°), JLCA 4.9° (range 0°–22°). Mean age at time of surgery was 10.5 years (range 2–16 years). Twenty-three knees (60.5 %) finished correction. Mean duration of correction was 22.2 months (range 7.8–43.2 months) the results showed following means: MAD 12.8 mm (range 0–68 mm), mLDFA 90° (range 74°–143°), MPTA 86.7° (range 76°–102°), JLCA 3.2° ± 2.8°. Complications were found in 7 patients (9 knees), namely knee stiffness (2), backing out of the screws and plates (4) and screw failure (1). Three knees did not show improvement. In the ankle group, preoperative mean LDTA measured 77.8° (range 60°–98°). Mean age at time of surgery was 10.3 years (range 6.5–13 years). Seven ankles (70 %) finished correction. Mean duration of correction was 28.6 months (range 8–38.6 months). Mean LDTA after correction was 90.5° (range 82°–102°). No complications were seen.
*Conclusion*: The deformity correction trends in patients with sick physes were found to be very variable. Inherent pathology of the physis often called for technique adjustments and probably contributed to the unpredictability of final results.

## EP115

### Clinical and radiological long term follow-up of free vascularized fibula after tumoral resection in 7 children

Manon Bachy[1], Caroline Dana[1], Arielle Salon[1], Eric Mascard[1], Christophe Glorion[1]

[1]Hôpital Necker, Paris, France

**LEVEL-IV/Tumours**

*Purpose*: Vascularized epiphyseal transfer, including the diaphysis and the growth plate, is an option to treat epiphyseal defects after tumour resection in pediatric patients that: the procedure aims to replace the bone defect and restore longitudinal growth. This study analyzes the surgical technique and report long-term clinical and radiological results.
*Methods*: This retrospective analysis included 7 patients aged 2–8 years (mean age 5.7 years) treated between 1997 and 2008, with resection of a malignant tumour and free vascularized epiphyseal fibular transfer reconstruction. Preoperatively, the pathological diagnosis was Ewing tumor (n = 5), osteosarcoma (n = 2). The locations of the sarcomas were proximal femur (3), proximal humerus (3) and distal radius (1). The mean length of the vascularized fibular transfer was 12.7 ± 2.9 cm. Vascularization of the transplant was based on a single pedicle in 5 cases (fibular artery 3, anterior tibial artery 3) and on 2 pedicles in 2 cases. Internal osteosynthesis was always used: intramedullary wire in 6 cases and a plate in one case. At the donor-site, a distal tibiofibular arthrodesis was performed in 6 cases. All patients were given chemotherapy preoperatively and postoperatively. One patient received adjuvant radiotherapy.
*Results*: Mean follow-up was 8.4 years (range 2.8–11.5 years). All patients were alive and no recurrence has occurred. Functional outcome was good in 85 % of cases. Serial radiographs revealed significant transplant hypertrophy, proving bone integration in all cases. Growth was a reality and physis remained opened in 4 cases. Complications were: one infection, two nonunion, two fractures, one malalignment required surgical correction, and two common fibular nerve dysfuntions. One valgus of the ankle appeared in the patient where preventive syndesmodesis was not performed.
*Conclusion*: In small children, free vascularized fibular transfer with its associated epiphyseal segment can treat the bone loss, providing joint mobility and allowing bone growth of resected segment. In our experience, growing prostheses have not given good enough results in small children, and arthrodesis does not solve the growth problem.
*Significance*: Although there is a high complication rate, this difficult surgical technique of free vascularized epiphyseal fibula transfer is an interesting option when epiphyseal resection is needed in small children, providing potential for growth and function preservation.

## EP116

### Incidence of humeral lateral condyle fractures nonunion in children

Lior Shabtai[1], Alexandre Arkader[1], Alexander Broom[1], Ted Sousa[1], J. Lee Pace[1], Rachel Y. Goldstein[2]

[1]Childrens Hospital Los Angeles, Los Angeles, USA
[2]Children's Hospital Los Angeles, Los Angeles, USA

**LEVEL-IV/Trauma/Upper extremity**

*Purpose*: Nonunion following lateral condyle fracture is a known complication; however the specific incidence is yet to be documented. The purpose of this study is to analyze the incidence of nonunion following lateral condyle fractures, as well potential significant contributing factors to a non-union.
*Methods*: Retrospective chart review of children under the age of 15 that were diagnosed with a lateral condyle fracture between 2001 and 2014 at one level-1 tertiary pediatric center. Information collected included demographic data, past medical history, lateral condyle classification, time from injury to surgery, operative versus non operative management, open versus closed reduction and type of fixation. Nonunion was defined as no fracture callus or bony consolidation seen on X-ray after 8–12 weeks following definitive management. Patients with <4 weeks of follow up and potentially complicating medical history were excluded from this study.
*Results*: 500 children were available for review. There were 315 (63 %) boys and 185 (37 %) girls. The average age at injury was 5 years (range—0.6–14.7). The average follow up was 10 weeks (range 4–110 weeks). 182 (36.4 %) were Type 1 fractures, 140 (28 %), Type 2 and 178 (35.6 %) Type 3. 168 (33.6 %) were treated in a cast without reduction, 121 (24 %) by closed reduction and percutaneous fixation (CRPP), and 211 (42 %) by open reduction and fixation (ORPP). In the ORPP group, 10/211 (4.7 %) were fixed with a screw instead of K-wires. The incidence of nonunion was 2 % (10/500), of which 2/140 (1.4 %) were type 1 fractures, treated in a cast; 1/178 (0.55 %) type 2 fracture, treated by ORPP: and 7/168 (4.2 %) type 3 fractures, of which two were treated by CRPP and 5 by ORPP. Type 3 fractures were associated with nonunion ($p = 0.05$). There was no significant correlation between type of fixation and nonunion ($p = 0.5$), gender and nonunion ($p = 0.57$), age and nonunion ($p = 0.37$) and time from injury to surgery and nonunion ($p = 0.69$).
*Conclusion*: Nonunion following lateral condyle fracture is a known complication but fortunately appears to be rare with a rate of only 2 %. Type 3 fractures are significantly associated with nonunion.
*Significance*: This is the first description of the incidence and risk factors of non-union after lateral condyle fracture in the literature.

## EP117

### 3D gait analysis of the foot after calcaneo stop procedure

Tamas Terebessy[1], Laszlo Kiss[1], Gabor Grosz[1], Gyula Domos[1], Nikoletta Horvath[1], Sandor Kiss[1], George Szoke[1]

[1]Semmelweis University, Budapest, Hungary

**LEVEL-IV/Foot and ankle**

*Purpose*: Determinig the border between pathological pes calcaneovalgus and physiological flatfoot in children is difficult. In addition performing the Calcaneo-stop surgery for paediatric calcaneo-valgus feet is more contradictive. In a previous study the authors showed biomechanical alterations in the paediatric flatfoot during gait. Increased medial load on the sole, certainly increased calcaneo-valgus and forefoot abduction during the whole gait cycle. It decreased plantarflexion in terminal stance and increased forefoot supination relative to the hindfoot. The aims of the present study was to evaluate the biomechanical effects of Calcaneo-stop surgery and to assess wether the gait after the Calcaneo-stop procedure is physiological.
*Methods*: 12 patients aged 15.55 ± 2.98 years who underwent the Calcaneo-stop procedure were measured with a six camera and one force plate 3D gait analysis system (Vicon Oxford Metrics). Follow up time was more than 1 year in all cases. Valgus tilt of the calcaneus, abduction/adduction, pronation/supination of the forefoot, and dorsal/plantar flexion of the ankle were measured with the help of the Oxford Foot Model. The valgus/varus moments in the hip, knee and ankle were also assessed. Data of 14 healthy volunteers served as control.
*Results*: The valgus tilt of the calcaneus and forefoot supination in relation to the hindfoot turned into physiological values −3.53° ± 5.054° versus −3.49° ± 2.91° and 6.77° ± 7.83° versus 3.32° ± 3.55° respectively. However the forefoot abduction and the dorsal/plantar flexion in terminal stance showed no significant changes. Valgus moments in the ankle and in the knee decreased but were still not physiological (0.19 ± 0.05 vs. 0.59 ± 0.14 and 0.001 ± 0.002 vs. 0.17 ± 0.11 Nm). No changes in the moments around the hip were found.
*Conclusion*: Moderate deformities can be observed in paediatric calcaneo-valgus foot after Calcaneo-stop surgery. Kinematics and kinetics of the experienced correction could be modeled with 3D gait analysis. The biomechanics of the feet, which underwent Calcaneo-stop procedure, were, however, still not completely physiological.

## EP118

### Treatment of dynamic internal rotation of the lower limbs with soft tissue procedure in cerebral palsy children

Dohin Bruno[1], Hassan Al Khoury Salem[1], Elie Haddad[1]

[1]Jean Monnet University CHU, Saint Etienne, France

**LEVEL-III/Cerebral Palsy**

Dynamic internal rotation (DIR) during gait is one of the functional disorders presented in cerebral palsy (CP) walking children. Bone procedures as rotational osteotomy of femur (FRO) (and/or tibia) have been proposed in order to correct this trouble. Multilevel surgery (SEMLS) including bone procedure, implicates more difficult and longer rehabilitation for these patients. When bone rotation defect is moderate, the authors hypothesized that it could be tolerated and bone procedure avoided to the benefit of the patient, allowing faster rehabilitation. The authors developed a soft tissues procedure to improve DIR of the lower limbs in these patients. The aim of the study was assessment of the results of the procedure.
*Methods*: CP Patients, in whom internal rotation of the lower limbs was mostly in relation with dynamic disorders, underwent the soft tissue procedure instead of FRO as a part of the SEMLS. Femoral anteversion up to 40° was treated with bone osteotomy and patients were excluded of the study. When possible, the authors tested botulinum toxin treatment in the targeted muscles, previously to settle on the procedures of SEMLS. The soft tissues procedure included lengthening and transfer of hamstring muscles associated with tenotomy of the gracilis and gluteus minimus muscles. Reviewed patients were selected among all the adolescents treated with the procedure in the department, when patient complete data (clinical, kinematics and kinetics) were available. Data (foot intoeing, dynamic hip internal rotation, hip rotation moment) were collected before and after procedure. Moreover, feet deformities and their treatments have been analyzed. Follow-up was at least 1 year after SEMLS.
*Results*: Most of the 20 patients were improved and satisfactory functional result obtained. Foot intoeing was normalized and hip internal rotation significantly improved. No patients required subsequent FRO, nevertheless one patient required late tibia rotational osteotomy.
*Discussion*: DIR of the lower limbs is frequent in CP. Bone deformity as femoral anteversion in CP often is not as severe as supposed and the etiology of the dynamic trouble is then in relation with muscles contractures or spasticity. Dynamic part of lower limbs internal rotation has been recently discussed in the literature. The authors reported an efficient soft tissues procedure to improve significantly the dynamic trouble and advocated to reduce indication for bone procedure in internal rotation of lower limbs in order to make easier rehabilitation after SEMLS. L.O.E III

## EP119

### Iliac apophyseal graft application in osteochondritis dissecans: a novel treatment method

Christina Steiger[1], Dimitri Ceroni[1]

[1]Orthopédie pédiatrique, HUG, Genève, Switzerland

**LEVEL-IV/Other/varia**

*Purpose*: The management of osteochondritis dissecans stage 3 and 4 is challenging. Several treatment methods have been developed in the last decades but functional results are often unsatisfactory. For big lesions, bone grafting is the only solution but the ideal graft is difficult to find. The most important properties of such an osteochondral graft are its availability in an adequate size, low donor site morbidity and easy harvesting. In adults this type of graft does not exist. Children however have an unossified skeleton making it possible to consider the use of the iliac apophysis as a graft.
*Methods*: Between 2013 and 2014 we have treated four patients for osteochondritis dissecans stage 4 with an iliac apophyseal graft. Lesions were located once in the talus, once in the medial condyle of the femur and twice in the capitulum of the humerus and varied in size between $0.8 \times 1.5$ cm and $1 \times 2.5$ cm with a depth of >0.8 mm. Patients' age at the time of surgery was between 7 and 14 years.
*Surgical Technique*: The chondral lesion was debrided and loose cartilage and fibrotic tissue were removed. A rectangular space was created with chisels and curettes to facilitate press fit implantation of the apophyseal graft. In case of cyst formation a bone reamer was used to clean out the defect. An iliac apophyseal graft of the appropriate length was obtained just proximal of the anterior superior iliac spine. The apophyseal cartilage measured between 4 and 8 mm. The bone graft was then trimmed to the exact size of the defect with a depth of about 2 mm greater than the depth of the lesion and inserted in press fit technique. To achieve a smooth cartilage surface the protruding cartilage of the graft was shaped with a scalpel blade.
*Results*: MRI results at 3 or 6 months show full integration of the bone graft without transformation of the cartilage cap. Clinically patients report a reduction of pain and 3 of the 4 patients have already started light sportive activities.
*Discussion*: Our preliminary clinical and radiological results using an iliac apophyseal graft in the treatment of osteochondritis dissecans are very promising but long-term results are not yet available. Nevertheless, in our opinion this surgical technique is suitable in the treatment of osteochondral lesions in children and young adolescence.

## EP120

### Single event multilevel surgery and selective fascicular partial neurotomies: report of the first 25 cases

Bruno Dohin[1], Hassan Al Khoury Salem[2]

[1]Jean Monnet University CHU, Saint Etienne, France
[2]Jean Monnet University CHU, Saint Etienne, France

**LEVEL-III/Cerebral Palsy**

Single event multilevel surgery (SEMLS) is well known to be an efficient procedure to improve gait in children with cerebral palsy (CP). Most of the surgical treatments used in SEMLS involved soft tissues or bones in order to enhance the mechanical system of the lower limbs. Orthopedics disorders in CP are mostly the result of spasticity and muscle weakness. Some treatments of spasticity demonstrated efficiency to improve functional results if associated or followed by orthopedic surgery (i.e. SDR). Selective fascicular partial neurotomy (SFPN), which is successfully used in post-stroke spasticity in adults, has been reported with good results in CP. Since 10 years, the authors have performed SFPN simultaneously with SEMLS in order to treat spasticity in walking CP patients. The aim of the study was assessing results of the procedure.
*Methods*: In CP patients with gait abnormalities, the authors made distinction between orthopedic and spastic disorders. When gait troubles were mainly in relation with spasticity without any risk for muscle weakness (checked with botulinum toxin test) SFPN were performed. We reviewed charts and gait analysis (GA) data of the patients treated with the procedure during the last 5 years. Patients without complete charts or GA were excluded. Results of SEMLS and SFPN were assessed with clinical data (i.e. ROM, spasticity, IDE index), kinematic and kinetics data from GA (i.e. angular velocity, peak timing), and QOL questionnaire. Patients were assessed before and at least 1 year after surgical procedure.
*Results*: 10 diplegic and 15 hemiplegic patients were included. Spasticity was significantly improved. Enhancement of IDE index and power testified of mechanical improvement. Focusing on spasticity, curve slope was systematically improved in kinematics data. All patients were improved in kinematics and kinetics. None patient presented functional alteration
*Discussion*: In order to durably improved CP patients after SEMLS, the authors used SFPN to treat simultaneously spasticity. To date, in our knowledge, this is the first report of SEMLS and SFPN simultaneous procedures in CP patients lower limbs. The final aim of the procedure will be reached when long term follow-up will confirm stable and satisfactory results especially on spasticity and no recurrence of muscles contracture. Nevertheless these satisfactory first results encourage the authors to continue in this way. L.O.E III.

## EP121

### Multilevel surgery in cerebral palsy: focusing hip power?

Bruno Dohin[1], Hassan Al Khoury Salem[2]

[1]Jean Monnet University CHU, Saint Etienne, France;
[2]Jean Monnet University CHU, Saint Etienne, France

**LEVEL-III/Cerebral Palsy**

*Arguments*: Normal gait is initiated by forward imbalance and walking is maintained by bringing in the mechanical system a bit of energy at each gait cycle. Gait analysis shows muscles energy production in the kinetics data (power curves). In normal gait, power is produced on ankle and hip joints. It is widely known that cerebral palsy (CP) induces muscle weakness. Weakness, as others symptoms of CP as spasticity, in most of cases is allocated from distal to proximal with a decreasing gradient of severity. The aim of single event multilevel surgery (SEMLS) in CP is restoring gait as normal as possible, so restoring hip and ankle power should be a good index for evaluation.
The aim of the study was to examine evolution of hip and ankle power in gait analysis in CP patients before and after SEMLS.
*Methods*: In the population of cerebral palsy patients we treated with multilevel surgery, we selected the last 20 patients who fulfilled inclusions criteria: diplegic (DCP) or hemiplegic cerebral palsy (HCP) children treated at the end of growth, with complete gait analysis data before and, at least, 1 year after SEMLS. We studied hip and ankle power before surgery and at final follow-up.
*Results*: Analysis confirmed that ankle power was in most of cases severely reduce in pathologic limbs. Hip is the main joint which gives some energy in gait in CP patients. Multilevel surgery failed in restoring ankle power in most of cases; nevertheless hip power could be improved or preserved in all cases.
*Discussion and Clinical Relevance*: SEML is indicated in cerebral palsy with the aim of restoring gait as normal as possible. This aim cannot be reach without maintaining propulsion or improve it. Discussion about surgical programs in SEMLS must include not only anatomic disorders but also dynamic disorders and take into account muscle weakness and spasticity. Hip and ankle power must be analyzed in order to avoid failure of SEMLS and sometimes improve result of SEMLS by focusing treatment: i.e. procedure with dynamic improvement on hip in which power could be improved and procedure with anatomic improvement on distal joints in which power cannot be obviously improved.
Authors suggest paying attention to hip and ankle power when SEMLS is discussed in walking CP patients. Propulsion is a part of efficient gait and therefore of a satisfactory results of SEMLS. Level of evidence III.

## EP122

### Range of motion of the upper extremity in a healthy pediatric population: introduction to normative data

Kai Ziebarth[1], Stefanie Nunes da Paz[1], Steffen Berger[1]

[1]Childrens University Hospital Bern, Bern, Switzerland

**LEVEL-II/Basic science**

*Purpose*: Traumatic injuries of the upper extremity are common in the pediatric population. Depending on the trauma, casting and immobilization are necessary and may lead to a loss of range of motion. In these cases as well as in pediatric congenital diseases it has to be decided if the mobility of the joint is within a normal range or if there is an indication for further therapeutic management. So far there is little data to be found defining a normal ROM of the upper extremity of the pediatric population. This study gives an introduction to normative data and demonstrates the influence of age on the range of motion of the upper extremity of healthy adolescents.
*Methods*: We performed an Institutional Review Board approved prospective study of healthy girls and boys aged from 2 to 16 years without any medical history of an upper extremity fracture or an underlying musculoskeletal disease. We investigated the active range of motion of the following joints: elbow, wrist, metacarpophalangeal and interphalangeal joints. Furthermore age, handedness, weight and height were recorded. 171 adolescents with a mean age of 10.6 years were included. 86 (50.3 %) participants were females and 85 (49.7 %) were males. To assess the effect of the age, the probands were separated into four cohorts: 2–5, 6–10, 11–13 and 14–16 years.
*Results*: Significant differences were found mainly in the 11–13 year age group. In this cohort the elbow flexion ($P = 0.01$), forearm pronation ($P = 0.01$), flexion of the interphalangeal joint of the thumb ($P = 0.0004285$) as well as the flexion of the metacarpophalangeal joints of digitus II–V ($P = 1.084e{-}05$, $P = 2.608e{-}05$, $P = 8.709e{-}05$, $P = 0.00045$) showed statistically significant differences. Furthermore a significant difference in the same joints excluding the elbow flexion was represented between the genders independent of the age groups.
*Conclusion*: This study contributes normative data for upper extremity range of motion in a healthy pediatric population that can be used for clinical reference.

## EP123

### Prevalence of specific gait abnormalities in children with cerebral palsy: influence of age, gender and GMFCS level

Gideon Blumstein[1], Tishya Wren[1], Susan Rethlefsen[1], Rachel Goldstein[1], Robert Kay[1]

[1]Childrens Hospital Los Angeles, Los Angeles, USA

**LEVEL-IV/Cerebral Palsy**

*Purpose*: Cerebral palsy (CP) is the most common motor disability of childhood. Gait abnormalities are common in children with CP, and the prevalence of specific gait deviations may be impacted by various factors. The purpose of this study was to determine the prevalence of 22 specific gait abnormalities and the effect of patient age, gender and Gross Motor Function Classification System (GMFCS) level on these gait abnormalities.
*Methods*: We retrospectively reviewed a consecutive series of 1,013 patients with CP [594 female, 419 male; mean age 9.8 ± 4.4 years, range 3–25; GMFCS distribution: 28 % (284/1013) GMFCS I, 32 % (324/1013) GMFCS II, 29 % (289/1013) GMFCS III, 11 % (116/1013) GMFCS IV] who underwent computerized motion analysis between January 1993 and May 2014. The prevalence of 22 specific gait abnormalities was determined for each GMFCS level, and multivariate logistic regression analysis was carried out to determine the influence of GMFCS level, patient age and gender on the prevalence of each gait abnormality.
*Results*: The most common gait problems in the entire cohort were in-toeing (71 %), crouch (66 %), stiff knee (64 %), hip flexion (58 %), hip internal rotation (57 %) and equinus (50 %). Females were significantly more likely to experience anterior pelvic tilt (OR 1.58, CI 1.22–2.04, $p = 0.01$), but less likely to experience crouch (OR 0.76, CI 0.59–0.99, $p = 0.05$) or posterior pelvic tilt (OR 0.51, CI 0.34–0.78, $p = 0.02$). The following gait abnormalities were significantly more prevalent in older patients: calcaneus, pes valgus, crouch, out-toeing, hip flexion, external tibial torsion and rotational mal-alignment. Other gait abnormalities were significantly more prevalent in younger patients: equinus, scissoring, in-toeing and internal tibial torsion. The prevalence of 14/22 gait abnormalities examined varied significantly based on GMFCS level, with crouch gait and stiff knee becoming progressively most common at higher GMFCS levels.
*Conclusion*: This study demonstrates a significantly different pattern of gait abnormalities in pediatric patients with CP based on GMFCS level, age and gender. To our knowledge, a breakdown of specific gait abnormalities by GMFCS level has not been previously reported.
*Significance*: This information is clinically valuable to help guide care and monitor progression of mobility and gait abnormalities in patients with CP.

## EP124

### Percutaneous correction of persistent severe metatarsus adductus in children

Jorge Knorr[1], Francisco Soldado[1], Paula Diaz-Gallardo[1], Jerome Sales de Gauzy[2]

[1]Hospital Sant Joan de Deu, Barcelona, Spain
[2]Hospital Sant Joan de Deu, Toulouse, France

**LEVEL-II/Foot and ankle**

*Background*: Percutaneous techniques for the correction of foot deformities are gaining in popularity in the adult population, but remain poorly explored in children. Of the several surgical techniques described to treat persistent severe metatarsus adductus deformity in children, neither are percutaneous. The purpose of the study is to describe a percutaneous technique for metatarsus adductus correction in children, to report the outcomes and to discuss the advantages it offers.
*Methods*: We undertook a prospective study in 34 consecutive feet with metatarsus adductus deformity from 26 children undergoing percutaneous correction. All operated feet had severe, rigid MTA deformities, most of which were components of residual/recurrent clubfoot deformities. Mean age at surgery was 5.7 years, and mean follow-up was 55.2 months. For clinical evaluation, we employed the bisector method, first cuneo-metatarsal angle and metatarsal-metaphyseal angle measured in weight-bearing radiographs. The AOFASf score were determined pre- and postoperatively. In unilateral cases, we used the contralateral foot measurements as control. The operating time and the hospitalization time were also recorded.
The surgical technique consisted of performing the Cahuzac's procedure for MTA correction with a percutaneous approach.
*Results*: At final follow-up all feet presented a normal heel-bisector line. Radiologic parameters were normalized when compared with control feet. Mean surgical and hospitalization time was 14 min and 6 h respectively. Mean AOFAS score improved from 78 to 98.
*Conclusion*: A minimally invasive percutaneous technique allowed a successful correction of metatarsus adductus deformity in children and resulted in a substantive decrease in both surgical and hospitalization time with better cosmetic results.

## EP125

### A 10-year multi-center review of current trends in the treatment of pediatric femoral shaft fractures in high volume academic centers

John Roaten[1], Jeffrey Sawyer[1], Alice Moisan[2], Joseph Yellin[3], John M. Flynn[3], Sumeet Garg[4], Micaela Cyr[4], Lindsay M. Andras[5], Alexander M. Broom[5]

[1]University of TN-Campbell Clinic, Germantown, USA
[2]Le Bonheur Children's Hospital, Memphis, USA
[3]CHOP, Philadelphia, USA; [4]Children's Hospital Colorado, Denver, USA; [5]CHLA, Los Angeles, USA

**LEVEL-III/Trauma/Lower extremity**

*Purpose*: Age-based guidelines have been proposed for the treatment of pediatric femoral shaft fractures. Due to the lack of a nationwide surgical database, the purpose of this study is to determine the current trend in treatments and whether or not age-based guidelines are being strictly followed at high-volume, geographically separated, academic pediatric trauma centers and to evaluate variability between centers.
*Methods*: A retrospective review of the data for the treatment of femoral shaft fractures from 4 high-volume geographically distant Level I pediatric trauma centers was performed between 2004 and 2014 using CPT codes linked to the treatment of femoral shaft fractures. CPT codes for each center and as a whole were grouped by age to evaluate age-based treatment. Age and center-based treatment patterns, including year-by-year analysis, were reviewed.
*Results*: A total of 2,483 patients were identified for the time period from all centers combined. Combined data show a total of 1,427(58 %) patients with closed treatment, the majority of which were treated with casting (1,171 patients, mean age 2.7 years). The next largest group, intramedullary nail (IMN) fixation totaled 760 patients (31 %), of which 474 were flexible nails and 279 were interlocked nails. The mean age of the combined flexible nail group was 7.7 years (range 1.5–17.1) and interlocked nail group was 13.3 (range 6.9–18), respectively. Furthermore, the IMN group showed 29 % of the interlocked nails were placed in patients <12 years of age, and 18 % of all flexible nailing patients were in patients <5 years of age. Locked nailing in patients <12 years had the largest increase in patients outlying the age based approach by 5 %. Open plating technique was used in 8 % of patients and increased from 14 to 30 %, with an average age of 9.2 years. Of the patients treated with open plating, 74 % were performed at a single center. External fixation was used in 47 patients (2 %). Skeletal traction as a definitive treatment was used in 0.6 % of all patients.
*Conclusion*: Considerable deviation from age based guidelines exists, with increasing trends in surgical treatment in patients <5 and expansion of age indications for flexible and locked nailing. In addition, some treatment methods are institution-dependent, even among high-volume academic pediatric trauma centers.
*Significance*: Further analysis of the patients that were treated outside of the guidelines as well as inter-center variability may lead to improved methods and guidelines for the treatment of pediatric femoral shaft fractures.

## EP126

### UK department of health recommendation on vitamin D supplementation for children: are we failing our children?

Rosalyn Drury[1], Simran Johal[1], Robert Nadler[1], Andreas Rehm[1]

[1]Cambridge University Hospitals, Cambridge, UK

**LEVEL-IV/Metabolic**

*Purpose*: There is increasing concern that vitamin D deficiency poses a major health problem for children. Deficiency can cause hypocalcemic seizures, tetany, growth disturbances and rickets and may influence diabetes, cardiovascular dysfunction and autoimmune diseases. Vitamin D deficiency was found to be higher in children with attention deficit hyperactivity disorder. Davies et a reported that 40 % out of 187 children and Foley et al. that 88 % out of 115 children presenting to a paediatric orthopaedic service who had their vitamin D levels measured had reduced vitamin D levels.

In the UK recommendations on vitamin D supplementation are set by the Department of Health (DoH) and the National Institute of Health and Clinical Excellence (NICE). These state that all children aged 6 months to 5 years ought to receive 7–8.5 µg of vitamin D daily.
*Methods*: We carried out a survey of 203 parents attending our paediatric and paediatric orthopaedic and fracture clinics to evaluate parental awareness of the DoH recommendations on vitamin D supplementation for children and to assess the extent to which children were receiving vitamin D supplements.
*Results*: Our study demonstrated that 85.71 % of parents were not aware of the recommendations. Only 14.29 % stated that they were aware of the benefits of vitamin D for their children and just 17.73 % (n = 36) of children were receiving vitamin D supplementation; 17 via formula milk and 18 via drops or some alternate multi-vitamin formulation.
*Conclusion*: We conclude that parents are generally not aware about Vitamin D supplementation because of a lack of dissemination of information. The high rates of reported sub-optimal vitamin D levels amongst children are not being addressed resulting in increased health risks for our children. Major improvements are needed in the implementation of supplementation at all points of contact between parents and health care professionals.

## EP127

## Innovative technique to obtain correct alignment of joint orientation angle with the anatomical axis of long bone when correcting long bone deformites in adolescents with locked intramedullary nails

Farhan Ali[1], Arif Razak[1], Sudheer Akkena[1], Emmanuel Morakis[1], Mubashshar Ahmad[1]

[1]RMCH, Manchester, UK

**LEVEL-IV/Trauma/Upper extremity**

*Puropose*: Acute deformity correction with the help of osteotomies and stabilization with an intramedullary (IM) nail system is a recognized surgical technique. Frequently, in order to obtain accurate correction, an external fixator is utilized to align the bone segments, which are then stabilized with the locked IM nail. This can be both time consuming and costly. This study describes an innovative technique that aids accurate intraoperative insertion of the initial guide pin in a direction that will lead to a correct joint orientation angle alignment of the long bone after osteotomy and insertion of IM nail. We also present our case series of long bone deformity correction in adolescent patients using this technique.
*Methods*: A retrospective review of casenotes and radiographs was performed of five patients (7 femurs and 4 tibias) who underwent long bone deformity correction using this technique. Average age of patients was 15.2 years (13–16.5 years). The underlying diagnoses were: hypophosphataemic rickets (3), growth arrests (1) and juvenile osteoporosis (1).
*Technique*: On preoperative radiographs, the distance of the joint surface from the point of intersection of the joint orientation angle line with the convex cortex and also its distance from apex of deformity were noted.
These distances were reproduced intraoperatively by using a standard metal ruler and two spinal needles. Under fluoroscopy, a guidepin was inserted towards the premeasured point on the convex cortex, which was then reamed. An osteotomy was performed and an appropriately sized locked IM nail inserted with the help of temporary blocking screws, resulting in the correction of deformities.
*Results*: Length of followup was from 6 to 24 months. All osteotomies had healed at an average of 46 days. Of the eleven bone segments, nine had normal alignment (within 2.5° of normal or contralateral side). In two bone segments the alignment was between 2.5° and 5° from normal. Blocking screws were not used in these two cases. There were some complications: one patient developed tarsal tunnel syndrome and clawing of toes. There was complete recovery after tarsal tunnel release and lengthening of long flexor tendons. One patient required reoperation for removal of prominent locking screws. All patients are now skeletally mature with no signs of growth disturbances.
*Conclusions*: This is a simple technique of deformity correction. The key to improving the accuracy of correction are the intraoperative blocking screws and the use of oblique osteotomies to accommodate translation. We advocate prophylactic compartment and appropriate nerve releases for all cases of acute tibial deformity corrections.

## EP128

## The clinical observation on the acetabulum of DDH by MRI

Xinhong Pei[1], Yueqiang Mo[1], Ruixue Ma[2]

[1]Children's Hospital of Fudan U, Shanghai, China
[2]Children's Hospital of Fudan U, Shanghai, The Netherlands

**LEVEL-II/DDH**

*Purpose*: Infants with developmental dislocation of the hip (DDH) were assessed by magnetic resonance imaging (MRI) before and after harness treatment. The purpose of this study was to observe the imaging feature in different types of DDH based on age pre- and post-treatment. This may provide valuable information regarding prognosis and adequacy of management.
*Methods*: We observed a consecutive series of patients with DDH treated at our hospital by closed reduction (CR), preoperative and immediate postoperatively using MRI. 36 patients (49 hips) were involved in this study. 12 hips were type I, 24 hips of type II, and 11 hips of type III. We compared the position of labrum before and after operation in the different types of DDH. The rate of close reduction was grouped by age and DDH type. The improvement of AI and absorption of intra-acetabulum soft tissue also were assessed.
*Result*: The success rate of closed reduction was 95.91 % (47/49). With age, the labrum became thickened gradually, intra-articular soft tissues increased, the greater the joint spacing after reduction in DDH. All labrums were inverted in Type I DDH, almost half of them (5/12) were still there after close reduction as type II, while labrums return the right position mostly in type III. The improvement of AI was highly significantly correlated with age. Soft tissue uptake tended to stop after 9 months in all cases.
*Conclusion*: Indices on the MRI scan suggest the importance of early reduction of the hip based on the atrophy of the labrum with age. It might be more prone to residual acetabulum dysplasia, because of more labrum inverted in type I DDH. MRI scan can be measured reliably, assessing acetabulum geometry, and labrum status after reduction.

## EP129

### Relationship between pelvic incidence and osteoarthritis of the hip

Jeremy Gebhart[1], Michael Bohl[2], Jonathan Streit[1], Raymond Liu[1]

[1]Rainbow Babies and Children's, Cleveland, USA
[2]Brown University, Providence, USA

**LEVEL-III/Hip/Lower extremity**

*Introduction*: Hip osteoarthritis (HOA) is a major cause of pain and disability that results in considerable social and medical costs. Mechanics such as posture, alignment and orientation of the hip and spinal column, and the relationship between these factors have been implicated in the development of both hip and spine pathologies. We investigated whether pelvic incidence (PI), a position-independent anatomic parameter that regulates lumbar lordosis and pelvic orientation, is associated with accelerated development of HOA.
*Methods*: We studied 400 well-preserved cadaveric skeletons ranging from 50 to 79 years of age at death. Specimens with obvious abnormalities were excluded. For each skeleton, degenerative disease of the acetabulum, proximal femur, distal femur, and proximal tibia were each graded from 0 to 3. Each specimen's left acetabulum, left proximal femur, right acetabulum and right proximal femur grades were combined to allow for scores ranging from 0 to 12 for "HOA." Distal femur and proximal tibia grades were combined in a similar fashion to determine "knee OA" scores. PI was measured from standardized lateral photographs of reconstructed pelvi. Multiple regression analysis was then performed to determine the relationship between age and PI and degenerative disease of the hips and knees. Two authors independently measured PI angles and graded OA, to account for inter-observer reliability.
*Results*: Average age was 60.2 ± 8.1 years, and average PI was 46.7° ± 10.7° (range 11.5°–78.1°). Multiple regression analysis did not demonstrate any correlation between PI and knee OA or PI less than our cohort mean (46.7°) and HOA, with Pearson correlations ranging from 0.02 to 0.05, and unstandardized Beta confidence ranges all containing zero. In contrast, results demonstrated a significant correlation between PI greater than our sample mean and hip OA (0.04), with a Pearson correlation of 0.14 and unstandardized Beta confidence range of 0.004–0.116. There was also a clear correlation between age and OA at both the hip and knee, with Pearson correlations ranging from 0.34 and 0.43, and no unstandardized Beta confidence ranges including zero.
*Discussion/Conclusions*: Our findings suggest that higher PI in the younger individual may contribute to the development of HOA in later life. This is the first study to demonstrate that the biomechanical adaptations of the pelvis and hip joints resulting from a larger PI may affect hip development and potentially accelerate the progression of HOA. Further investigation will be expected to analyse the role spino-pelvic alignment plays in the development of HOA.

## EP130

### A Systematic Review of Alternative Splinting Versus Complete Plaster Casts for the Management of Childhood Buckle Fractures of the Wrist

Christopher Hill[1]

[1]Uni Hospital Coventry/Warwick, Coventry, United Kingdom

**LEVEL-I/Trauma/Upper extremity**

Paediatric buckle fractures of the wrist are a common type of distal radius fracture that are inherently stable. Traditionally they have been managed by immobilisation in a complete plaster cast necessitating follow-up visits, time off school and work, inconvenience, and hospital treatment costs. Therefore treatment with alternative splints that negate the need for further hospital visits, saving both time and money has been proposed. However concerns regarding complications, primarily pain, have been raised, hence the topic remains controversial.

A systematic review of the relevant literature was performed and eight randomised control trials were identified, critiqued and analysed accordingly. Overall, alternative-splinting was found to be superior to casting in the treatment of paediatric wrist buckle fractures in terms of function, cost and convenience but crucially with no significantly worse pain or fracture complication levels.

The evidence is sufficiently robust to endorse the use of alternative-splinting over casting in paediatric wrist buckle fractures as a safe treatment method with significant benefits to both individuals and the health care service.

Open Access

This article is distributed under the terms of the Creative Commons Attribution License which permits any use, distribution, and reproduction in any medium, provided the original author(s) and the source are credited.

